# Site‐Selective Modification of Proteins with Oxetanes

**DOI:** 10.1002/chem.201700745

**Published:** 2017-03-29

**Authors:** Omar Boutureira, Nuria Martínez‐Sáez, Kevin M. Brindle, André A. Neves, Francisco Corzana, Gonçalo J. L. Bernardes

**Affiliations:** ^1^ Department of ChemistryUniversity of CambridgeLensfield RoadCB2 1EWCambridgeUK; ^2^ Instituto de Medicina MolecularFaculdade de MedicinaUniversidade de LisboaAvenida Professor Egas Moniz1649-028LisboaPortugal; ^3^ Li Ka Shing CentreCancer Research (UK) Cambridge InstituteRobinson WayCB2 0RECambridgeUK; ^4^ Department of BiochemistryUniversity of CambridgeTennis Court RoadCB2 1GACambridgeUK; ^5^ Departamento de QuímicaCentro de Investigación en Síntesis QuímicaUniversidad de La Rioja26006LogroñoSpain; ^6^ Current address: Departament de Química Analítica i Química OrgànicaFacultat de QuímicaUniversitat Rovira i VirgiliC/ Marcel⋅lí Domingo 143007TarragonaSpain

**Keywords:** antibodies, oxetanes, protein modifications, small oxygen heterocycles, sulfur

## Abstract

Oxetanes are four‐membered ring oxygen heterocycles that are advantageously used in medicinal chemistry as modulators of physicochemical properties of small molecules. Herein, we present a simple method for the incorporation of oxetanes into proteins through chemoselective alkylation of cysteine. We demonstrate a broad substrate scope by reacting proteins used as apoptotic markers and in drug formulation, and a therapeutic antibody with a series of 3‐oxetane bromides, enabling the identification of novel handles (S‐to‐S/N rigid, non‐aromatic, and soluble linker) and reactivity modes (temporary cysteine protecting group), while maintaining their intrinsic activity. The possibility to conjugate oxetane motifs into full‐length proteins has potential to identify novel drug candidates as the next‐generation of peptide/protein therapeutics with improved physicochemical and biological properties.

Oxetanes are heterocyclic 1,3‐propylene oxide moieties^1^ that constitute the core structure of many biologically active natural products (e.g. the antibiotic oxetin) and synthetic compounds (e.g. carbonyl bioisosters, agrochemicals, and peptidomimetics, among others).^2^ They have emerged as important motifs in drug discovery due to the modulation of the physicochemical properties of the molecule to which they are attached.^3^ Typically, oxetanes increase water solubility, metabolic stability, and reduce lipophilicity while maintaining their parent selectivity without altering activity (Figure 1). Despite the many examples reported for the introduction of such motifs in small molecules, their application to modify complex biomolecules, such as proteins/antibodies (beyond peptidomimetics),^4^ is largely unprecedented.

**Figure 1:**
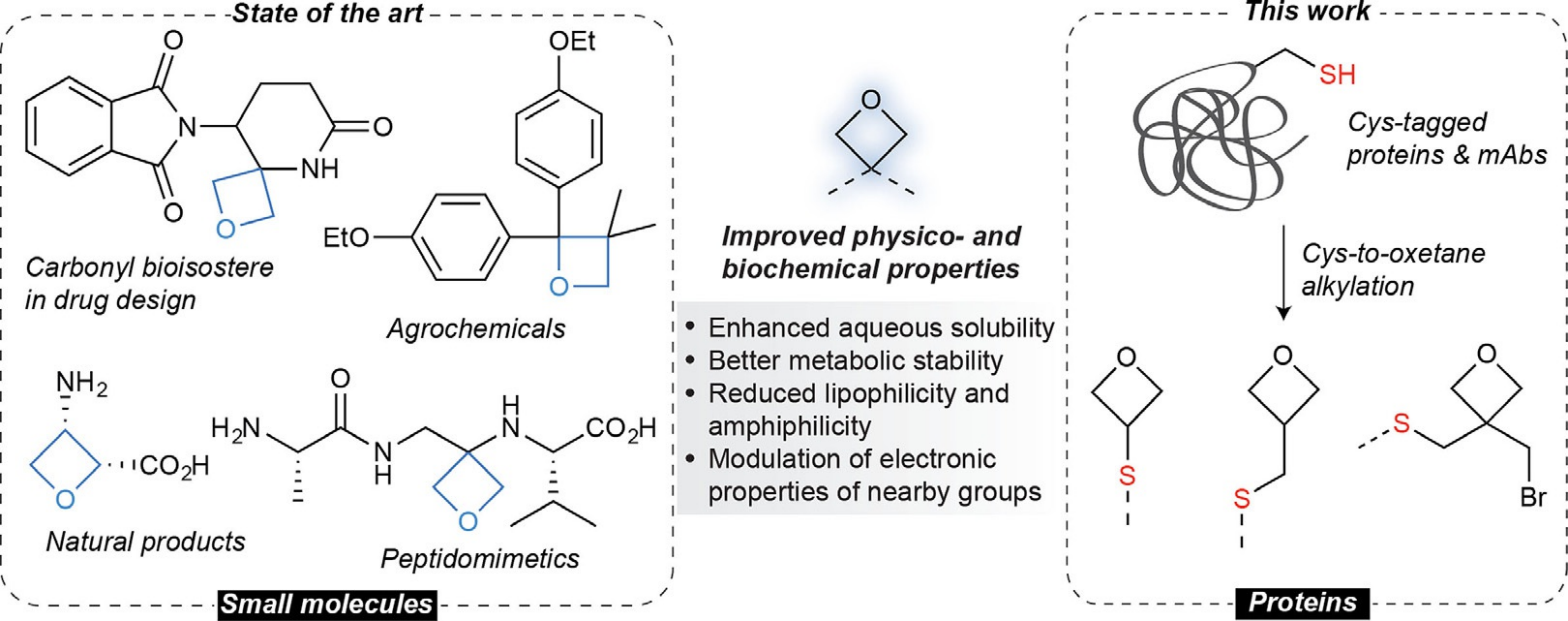
The oxetane motif present in small molecules (state of the art) and its application in site‐selective chemical protein modification (this work).

The aim of the present proof‐of‐principle study is to explore methods for the chemoselective introduction of oxetane moieties into proteins and antibodies by alkylation of the sulfhydryl group of the side chain of cysteine (Cys) with a series of structurally similar 3‐oxetane bromides. We reasoned that investigation of oxetane reagents (e.g. secondary vs. primary halides and 3‐mono vs. 3,3‐disubstituted) and their reactivity towards a number of different protein scaffolds would test the generality of this site‐selective protein modification method for the preparation of homogeneous proteins.^5^ We envisaged our method would allow access to a new family of protein conjugates with potential therapeutic applications,^6^ for example, by enhancing ligand–protein binding since the oxetane ring serves as a hydrogen‐bond acceptor.^7^

We began our investigation by exploring the site‐selective incorporation of the 3‐*S*‐oxetane motif into proteins by reacting inexpensive, commercially available 3‐bromooxetane **1** with single‐Cys mutants of the C2A domain of Synaptotagmin‐I Cys95 (C2Am) and Annexin‐V Cys315 (AnxV) as representative examples of specific apoptotic protein markers (Figures 2 a and 2b).^8^ Incubation of C2Am with **1** in 50 mm sodium phosphate buffer (NaP_i_) at pH 11 with up to 20 % DMF, to ensure oxetane's solubility, afforded expected Cys‐to‐oxetane alkylation product **2** in >95 % conversion (via an S_N_2 reaction) as determined by liquid chromatography electrospray ionization mass spectrometry (LC‐ESI‐MS) after 30 h at 37 °C. Remarkably, and unlike most of the alkylating electrophiles commonly used for “on protein” Cys alkylation (typically primary C(sp^3^) halides), the present transformation using a secondary C(sp^3^) bromide represents a stereoelectronically challenging, yet successful example of a constrained small oxygen heterocycle alkylation, which may account for the “harsh” conditions employed. The modified protein was identified by LC‐ESI‐MS, which showed a single peak (16 279 Da) with a mass shift corresponding to the addition of a single 3‐oxetane unit (+57 Da). Negative Ellman's test and peptide mapping/MS^2^ analysis revealed the alkylation proceed with exquisite chemoselectivity at Cys95 (Figure 2 c and the Supporting Information, Figures S1–S5). Experiments to further evaluate putative lysine (Lys) cross‐reactivity were conducted with single Lys‐peptide **S1** and 3‐bromooxetane **1** using conditions similar to those employed for “on protein” Cys alkylation (50 mm NaP_i_ at pH 11 with 20 % DMF). No reactivity was noticed after 36 h at 37 °C as monitored by ^1^H NMR (Supporting Information, Figure S77).

**Figure 2:**
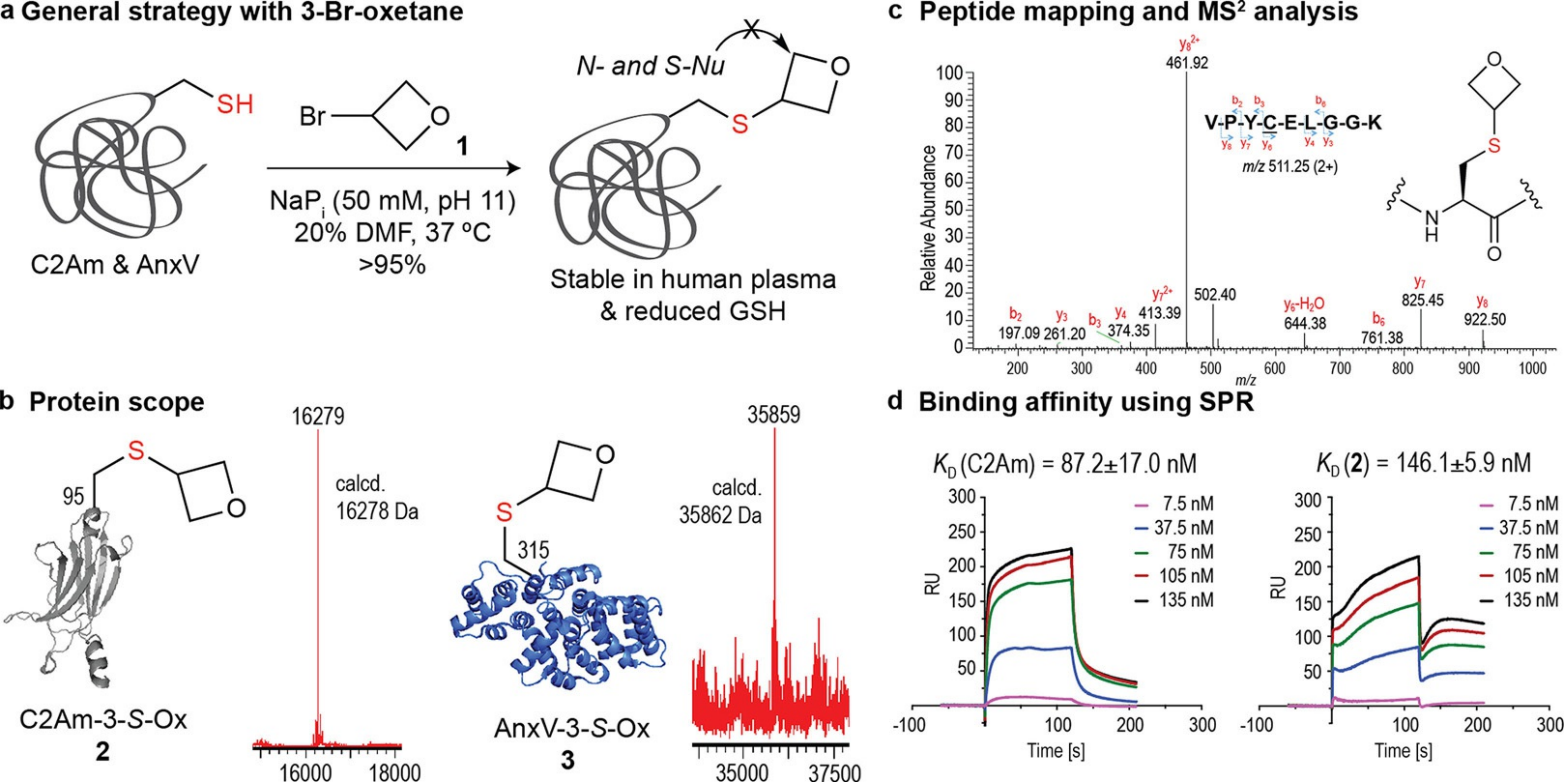
Selective incorporation of the 3‐*S*‐oxetane motif into proteins. a) General strategy with **1**. b) Protein scope with C2Am and AnxV. c) MS^2^ spectrum of the *m*/*z* 511.25 doubly charged ion of the tryptic peptide VPYCELGGK, containing the 3‐*S*‐oxetane modification at the original Cys95 residue in **2**; the fragment ions generated are consistent with the mass of the modification. d) Biacore SPR analysis of C2Am and **2** against PS; the data represent mean±SD obtained from two independent experiments performed in duplicate.

Importantly, the 3‐*S*‐oxetane motif proved stable upon incubation with human plasma (37 °C, 24 h) and in the presence of endogenous thiols such as reduced glutathione (GSH) (20 mm, 37 °C, 24 h), which are prerequisite conditions for in vivo applications. Identical stability was also noticed upon incubation with other S‐ or N‐nucleophiles (β‐mercaptoethanol (BME), PhSH, βGalSNa, and BnNH_2_) under forcing conditions (50 mm NaP_i_ at pH 4.5–11 with/without 20 % CH_3_CN, 37 °C, up to 24 h). Additionally, additives known to promote oxetane ring‐opening polymerization^9^ were also tested without success. These included oxophilic metallic complexes (Y(OTf)_3_ and MgCl_2_⋅6 H_2_O) and organocatalytic promoters (urea and thiourea) (Supporting Information, Figures S6–S21). The inertness of the oxetane ring towards nucleophiles is in line with previous studies that described their lack of geno/cytotoxicity and mutagenicity, unlike that of epoxides and β‐lactones, because they do not act as alkylating agents at physiological pH.^10^ Likewise, other proteins such as AnxV reacted similarly at pH 11 with 10 % DMF and expected product **3** was obtained in >95 % conversion, thus demonstrating the robustness of this alkylation protocol to modify this widely used apoptotic protein marker (Figure 2 b). Finally, the impact of such a modification and reaction conditions in protein's structure and function was evaluated using circular dichroism (CD) and surface plasmon resonance (SPR), respectively.^8^ The chemoselective incorporation of the 3‐*S*‐oxetane motif in **2** is mild as determined by CD analysis, which showed no structure alteration between native and modified proteins (Supporting Information, Figure S80). Additionally, despite the SPR functional assay for C2Am vs. C2Am‐3‐*S*‐Ox **2** shows that its intrinsic binding activity against phosphatidylserine (PS), an internal membrane lipid externalized during apoptosis, is only partially eroded (60 % activity is maintained), differences in its binding mechanism is noticed from the analysis of the binding and dissociation curves (Figure 2 d).

Having established conditions for efficient site‐selective incorporation of the 3‐oxetane motif into Cys‐tagged proteins, we explored the extension of this approach to other oxetanes. We continued by evaluating the homobifunctional electrophile 3,3‐bis(bromomethyl)oxetane **4** (Figure 3). We anticipate the facile alkylation of Cys due to the a priori more accessible, reactive primary C(sp^3^) bromide will enable efficient desymmetrization of **4**, thus providing a platform to chemoselectively incorporate an electrophilic handle (BrCH_2_‐*S*‐oxetane) into a predefined site of proteins. This handle is amenable to a second round of chemical modification (via an S_N_2 reaction) after incubation with an appropriate nucleophile (e.g. post‐translational modifications, cytotoxics, and spectroscopic tags, among others) (Figure 3 a). Indeed, this is a difficult task and very few chemical methods allow for the precise installation of electrophilic reacting points on a single residue,^11^ which is typically achieved by genetic encoding protocols.^12^ Thus, reaction of **4** with C2Am in NaP_i_ (50 mm, pH 8) with 10 % DMF afforded **5** in >95 % conversion as determined by LC‐ESI‐MS after 5 h at 37 °C. Fine‐tuning of the reaction conditions and using pH 11 reduced the reaction time to only 2 h, while maintaining chemoselectivity as confirmed by negative Ellman's test and ^1^H NMR experiments with Lys‐peptide **S1** (Supporting Information, Figures S22–S26 and S78). The protein scope was further expanded to AnxV that reacted similarly at pH 11 with 10 % DMF to obtain **6** in >95 % conversion (Figure 3 b and Supporting Information, Figures S54 and S56). We next set up to evaluate the incorporation of several S‐ and N‐nucleophiles taking advantage of the flexible introduction of the privileged 3‐bromomethyl‐3‐*S*‐oxetane handle. This represents a unique example of spiro, heterobifunctional S‐to‐S/N linker^13^ that enables the diversity‐oriented introduction of several nucleophiles from a single Cys mutant.^14^ To this end, **5** was incubated with the S‐nucleophiles βGalSNa, BME, and PhSH as representative examples of protein glycosylation, aliphatic, and aromatic moieties, respectively as well as BnNH_2_ as an N‐nucleophile present in several drug fragments to afford **7 a**–**d** in >95 % conversion (Figure 3 c). Extending the reaction time once the alkylation is completed (e.g. up to 15 h with PhSH) did not show any noticeable degradation (Supporting Information, Figures S27–S33). Moreover, similar to that observed with the 3‐*S*‐oxetane motif, S‐to‐S/N oxetane linker in **7 c** also proved stable upon incubation with human plasma (37 °C, 24 h) and GSH (20 mm, 37 °C, 24 h) (Supporting Information, Figures S34 and S35), which reinforces its application as a novel linker for stable, covalent protein modification (e.g. including disulfide stapling).^5^

**Figure 3:**
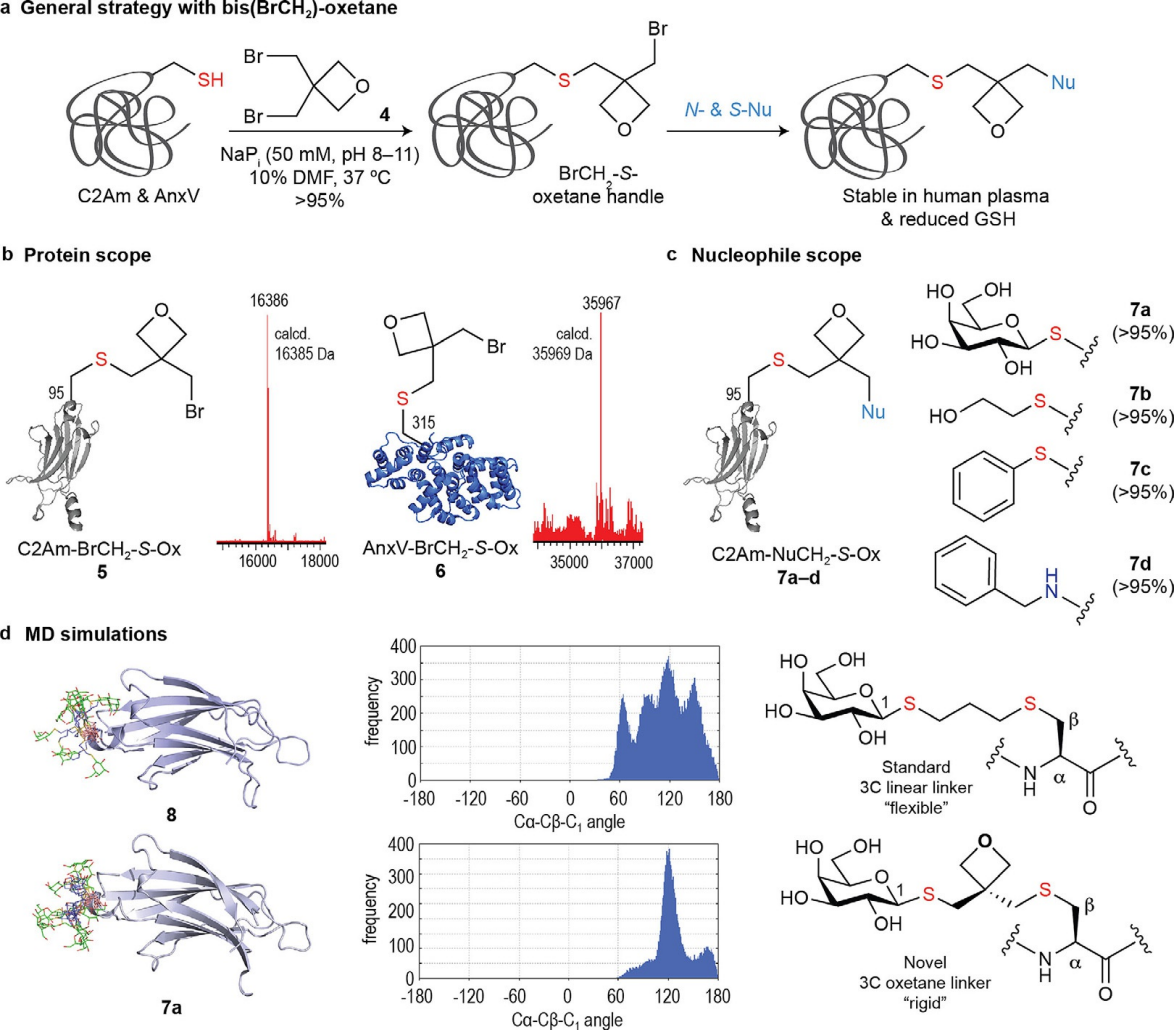
Selective incorporation of an S‐to‐S/N oxetane linker on proteins. a) General strategy with **4**. b) Protein scope with C2Am and AnxV. c) Nucleophile scope with **5**. d) Analysis of the flexibility of S‐to‐S/N oxetane linker in **7 a** (lower panel) vs. a standard 3C‐linear linker in **8** (upper panel) obtained from 100 ns MD simulations. The data presented in this Figure corresponds to the average structure of both linkers through the simulations.

The display of βGalS as a representative example of carbohydrate epitope was further studied by molecular dynamics (MD) simulations and the result compared to a general aliphatic linker with the same number of carbons, obtained by thiol‐ene chemistry at *S*‐allyl cysteine (Sac) (Figure 3 d).^15^ Interestingly, the oxetane linker in **7 a** proved to be a more rigid scaffold than that in **8** for the presentation of βGalS as determined by the analysis of the angle between Cα‐Cβ‐C_1_ where a major population is observed. Taken together, we believe this rigid, non‐aromatic, and soluble linker will be advantageous, for example, in the development of homogeneous carbohydrate‐based vaccine conjugates, hopefully, with reduced *anti*‐linker response.^16^

Encouraged by these results, we next evaluated the use of 3‐(bromomethyl)oxetane **9** (Figure 4). Experiments with a series of proteins (C2Am, AnxV, and recombinant human serum albumin (rHSA)) and one antibody (Trastuzumab) revealed the application of this moiety as a novel temporary protecting (PG) group for Cys‐containing biomolecules^17^ (via the following full sequence Cys‐protection→SCH_2_‐Ox→Cys‐deprotection) (Figures 4 a and 4b). Unlike previous reactions with 3‐oxetane bromides **1** and **4** that necessitated up to pH 11 or prolonged reaction times to reach completion, the alkylation with **9** proceed smoothly at pH 8 (for C2Am) and pH 9 (for AnxV), respectively to afford **10** and **11** in uniformly complete conversions with a mass difference corresponding to the incorporation of a single CH_2_‐oxetane moiety (+71 Da). Negative Ellman's test confirmed the chemoselectivity at Cys95 in C2Am and Cys315 in AnxV (Supporting Information, Figures S36, S37, S57, and S58). rHSA (Albumedix Recombumin),^18^ an approved ingredient for the manufacture of human therapeutics that possess multiple Cys residues, 17 structurally relevant disulfides and a single Cys at position 34, gave the expected monoalkylation product **12** (25 % conv.) upon incubation with 50 equiv of **9** in NaP_i_ (50 mm, pH 8) with 10 % DMF as determined by LC‐ESI‐MS after 2 h at room temperature. However, the addition of several 3‐methyloxetane units (+71 Da) was observed after prolonged reaction times by increasing the equivalents of **9** (>50 equiv) and warming the reaction up to 37 °C. We reasoned this might be attributed to the reaction of **9** with partially reduced disulfides (Supporting Information, Figures S64–S67).^19^ Similar results are also obtained with the monoclonal antibody (mAb) Trastuzumab containing several structural disulfides (Figure 5 a).^20^

**Figure 4:**
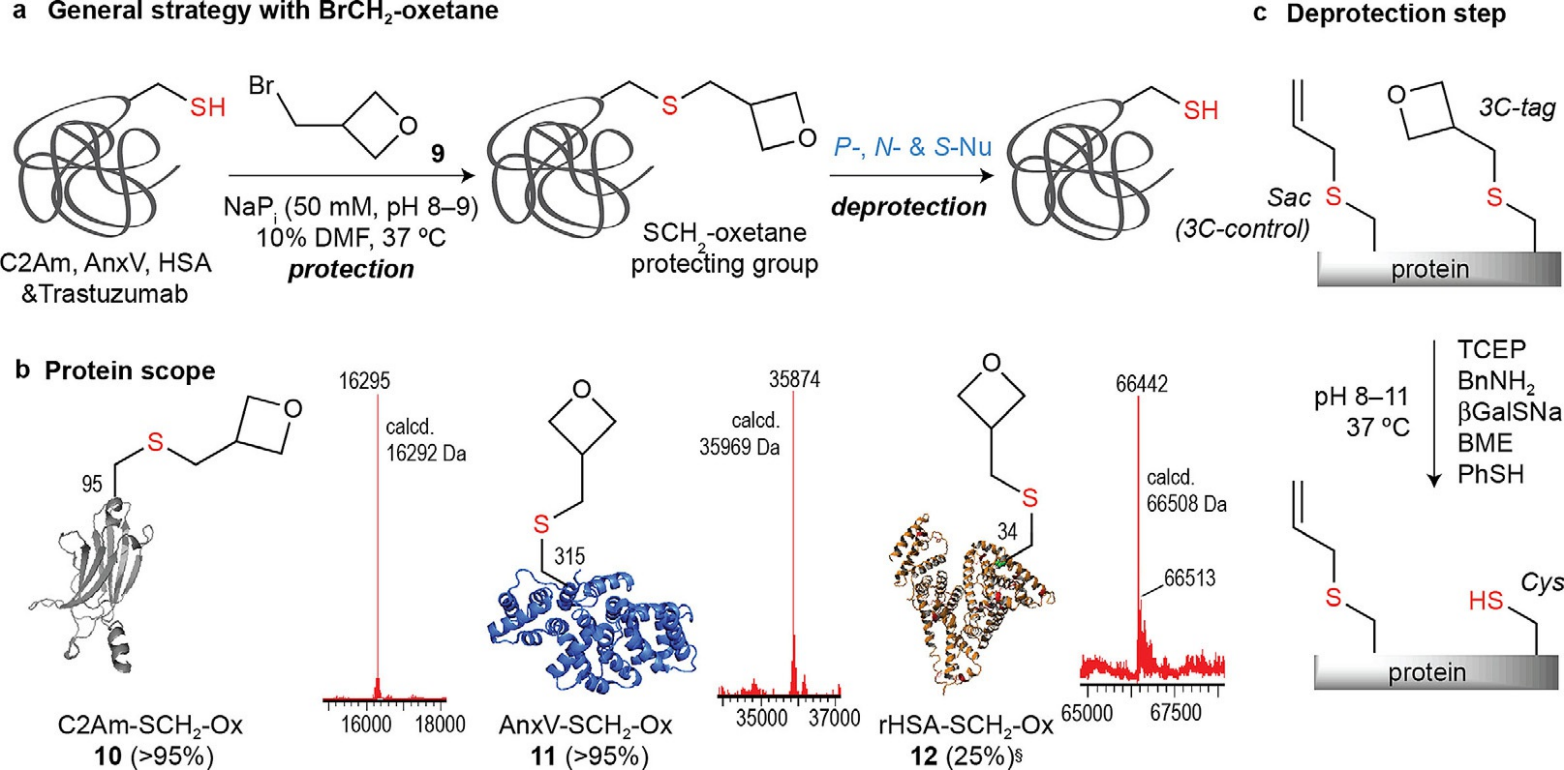
Selective incorporation of the SCH_2_‐3‐oxetane motif into proteins as a Cys temporary protecting group. a) General strategy with **9**. b) Protein scope with C2Am, AnxV, and rHSA. c) Schematic representation of the deprotection step (SCH_2_‐Ox→Cys) with several P‐, N‐, and S‐nucleophiles and unreactive controls using Sac (see the Supporting Information for details). ^§^ Multiple additions observed upon incubation with >50 equiv of **9**.

**Figure 5:**
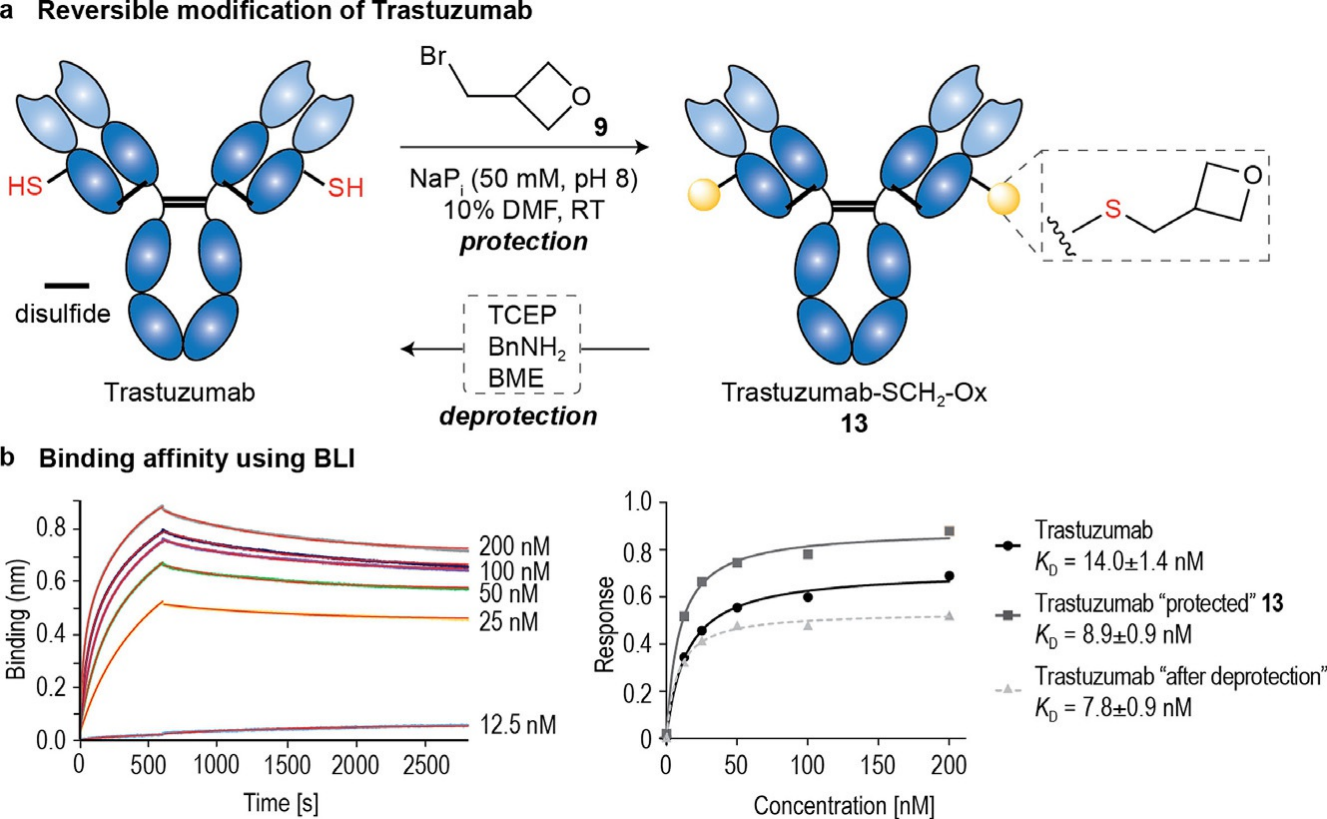
Reversible modification of Trastuzumab. a) General strategy with **9**. b) BLI analysis of Trastuzumab (unmodified and recovered) and **13** against HER2 receptor; the data represent mean±SD obtained from two independent experiments performed in triplicate.

We then examined the stability of 3‐*S*‐monomethyl oxetane proteins. Incubation of **10** with 100 equiv of BnNH_2_ in NaP_i_ (50 mm, pH 8) unexpectedly resulted in the full recovery of the original Cys moiety after 2 h at 37 °C (Figure 4 c). Interestingly, a conceptually similar N‐dealkylation reaction by oxidative cleavage of the CH_2_ bridge was found to be the main metabolization product in 3‐monomethyl oxetanes.^21^ This study also suggests, as we found in our work with the 3,3‐disubstituted analogue, the introduction of bulky *gem*‐dimethyl substituents increases the stability of the oxetane. A putative S‐dealkylation scenario in which the nucleophile attacks the alpha carbon (Cα) of the oxetane, releasing Cys as a leaving group perhaps with the participation/anchimeric assistance of the non‐bonding electron pair of the oxygen might account for the reactivity observed, although alternative mechanisms (e.g. oxidative cleavage with atmospheric O_2_)^22^ cannot be discarded. With these preliminary conditions in hand, the scope of the reaction was evaluated with a range of S‐, N‐, and P‐nucleophiles (BME, PhSH, βGalSNa, BnNH_2_, and tris(2‐carboxyethyl)phosphine (TCEP)) and proteins (**10** and **11**). Despite starting Cys‐proteins (C2Am and AnxV) were recovered in up to >95 % conversion, the rate of this transformation seems to be dependent on the combination of protein/nucleophile/conditions (Supporting Information, Figures S38–S47 and S59–S61). For example, deprotection of **10** was slower with both TCEP (9 h) and PhSH (22 h), necessitating a large excess of nucleophile (10,000 equiv) at 37 °C to reach completion, whereas βGalSNa required only 5 h (at pH 8) or 2 h (at pH 11). Similarly, AnxV was obtained from **11** after incubation with 100 equivalents βGalSNa in NaP_i_ (50 mm, pH 9) after only 1 h at 37 °C. Controls to demonstrate how oxetanes modulates the electrophilic character of the Cα enabling the S‐dealkylation when treated with excess S‐, N‐, and P‐nucleophiles were carried out using the Sac handle^23^ as a stable 3C‐surrogate of the S‐methyloxetane unit (SCH_2_‐Ox). Sac‐containing proteins C2Am **S2** and AnxV **S3** were subjected to the same S‐dealkylation conditions and starting materials were recovered unaltered. Thus, our preliminary findings suggest a role of the methyloxetane moiety in the nucleophile‐induced deprotection step, at least on full‐length proteins (Figure 4 c and Supporting Information, Figures S48, S49, S62, and S63).^24^

Finally, the utility of our protocol was extended to the reversible modification of Trastuzumab; a mAb used in the clinic for the treatment of HER2+ metastatic breast and gastric cancers. We found the Cys‐engineered Trastuzumab analog (Thiomab 4D5 LC‐V205C)^20^ reacted with **9** in NaP_i_ (50 mm, pH 8) with 10 % DMF to afford modified **13** containing several (3‐methyloxetane)_*n*_ units (+71_n_ Da) in the light chain as determined by LC‐ESI‐MS after 5 h at room temperature (Figure 5 a and SI, Figures S68 and S69).^25^ Similarly to 3‐oxetane bromides **1** and **4**, ^1^H NMR experiments with **9** and single Lys‐ and single Cys‐peptides **S1** and **S4**, respectively demonstrated the introduction of these multiple 3‐methyloxetane units is due to alkylation of the free engineered Cys together with those resulting from inefficient re‐oxidation of structural disulfides^20^ rather than for Lys cross‐reactivity (Supporting Information, Figure S79).

The (SCH_2_‐Ox)‐to‐Cys deprotection in **13** was triggered by TCEP, BnNH_2_, or BME and represents a successful metal/light‐free example of a protection–deprotection sequence on an intact IgG mAb (Supporting Information, Figures S70–S73). We then investigated the influence of incorporation/removal of SCH_2_‐Ox on mAb's function by bio‐layer interferometry (BLI) analysis and compared its activity before and after modification. We found, the binding affinity is maintained along the entire protection‐deprotection cycle with their binding constants remaining within the same nanomolar range (Figure 5 b and Supporting Information, Figures S83 and S84). We expect this protocol will find use as a tool for transient, site‐selective manipulation of Cys on proteins and antibodies.^26^

In summary, we disclosed an operationally simple and advantageous method for the chemoselective introduction of oxetane moieties into proteins through alkylation of Cys residues. We validated this mild transformation on a series of proteins and antibodies, which maintained their inherent activities. Screening of oxetane variants enabled the discovery of novel handles (spiro S‐to‐S/N linker) and reactivity modes (temporary Cys‐PG). This work provides the basis for the development of novel oxetane reagents and complements current methods for site‐selective chemical protein/peptide modification.^5^ We anticipate the knowledge derived from this thorough proof‐of‐principle study will help in the selection of reaction conditions suitable for introducing other strained heterocyclic motifs opening new horizons in the field of protein engineering and biological therapeutics.^6^

## Conflict of interest

The authors declare no conflict of interest.

## Supporting information

As a service to our authors and readers, this journal provides supporting information supplied by the authors. Such materials are peer reviewed and may be re‐organized for online delivery, but are not copy‐edited or typeset. Technical support issues arising from supporting information (other than missing files) should be addressed to the authors.

Supplementary
