# Supplementary material for: Site‐Selective Modification of Proteins with Oxetanes
Source: Chemistry. 2017 Mar 29;23(27):6483–9. doi: 10.1002/chem.201700745 (PMC5434895; doi:10.1002/chem.201700745)
Supplement: Supplementary file 1 — Supplementary [file CHEM-23-6483-s001.pdf]

CHEMISTRY
A European Journal

Supporting Information

Site-Selective Modification of Proteins with Oxetanes

Omar Boutureira,*[a, f] Nuria Martínez-Sáez,[a] Kevin M. Brindle,[c, d] André A. Neves,[c] Francisco Corzana,[a, e] and Gonçalo J. L. Bernardes*[a, b]

chem_201700745_sm_miscellaneous_information.pdf

Table of Contents

1. General Remarks S2

2. Experimental Section S6

    2.1. Protein Modification S6

    2.2. LC–MS/MS Analysis S65

    2.3. SDS Polyacrylamide Gel Electrophoresis (SDS-PAGE) S66

    2.4. ^{1}H-NMR Experiments S67

    2.5. Circular Dichroism (CD) S70

    2.6. Molecular Dynamics (MD) Simulations S70

    2.7. Surface Plasmon Resonance (SPR) S71

    2.8. Bio-Layer Interferometry (BLI) S72

3. References S73

1. General Remarks

Proton (^{1}H-NMR) nuclear magnetic resonance spectra were recorded on a Bruker 400 MHz. Spectra were fully assigned using COSY, HSQC, HMBC and NOESY. All chemical shifts are quoted on the δ scale in ppm using the residual solvent as internal standard. Coupling constants (*J*) are reported in Hz with the following splitting abbreviations: s = singlet, d = doublet, t = triplet, q = quartet, quin = quintet and app = apparent. The results of these experiments were processed with MestreNova software. High-resolution mass spectra (HRMS) were recorded on a Waters ZQ LCMS using electrospray ionization (ESI) or on a Waters LCT Premier spectrometer. Nominal and exact *m/z* values are reported in Daltons. All solvents were used as supplied (Analytical or HPLC grade), without prior purification. Distilled water was used for chemical reactions and Milli–QR purified water for protein manipulations. All reagents were used as received from commercial suppliers. Solid-phase syntheses were carried out in peptide-synthesis reaction vessels (25 or 50 mL) with coarse porosity fritted glass support and Teflon stopcocks. Prep-scale reverse-phase chromatography was conducted with Agilent 1100 Series HPLC Value System (YMC-Pack Pro C18 column, 5 µm, 250 mm × 10 mm). The eluent was acetonitrile (HPLC grade) with 0.1% trifluoroacetic acid (TFA) and Millipore water with 0.1% TFA.

Protein Mass Spectrometry

Liquid chromatography-mass spectrometry (LC–MS) was performed on a Xevo G2-S TOF mass spectrometer coupled to an Acquity UPLC system (Acquity UPLC BEH300 C4 column, 1.7 µm, 2.1 mm × 50 mm). Water with 0.1% formic acid (solvent A) and 70% acetonitrile and 29% water with 0.075% formic acid (solvent B), were used as the mobile phase at a flow rate of 0.2 mL min^{-1}. The gradient was programmed as follows: From 72% A to 100% B for 25 min then 100% B for 2 min and 72% A for 18 min. The electrospray source was operated with a capillary voltage of 2.0 kV and a cone voltage of 40 V. Nitrogen was used as the desolvation gas at a total flow of 850 L h^{-1}. Total mass spectra were reconstructed from the ion series using the MaxEnt algorithm preinstalled on MassLynx software (v. 4.1 from Waters) according to the manufacturer's instructions.

Protein LC–MS/MS analysis

The protein sample was enzymatically digested by trypsin overnight. All LC–MS/MS experiments were performed using a nanoAcquity UPLC (Waters Corp., Milford, MA) system and an LTQ Orbitrap Velos hybrid ion trap mass spectrometer (Thermo Scientific, Waltham, MA). Separation of peptides was performed by reverse-phase chromatography using a Waters reverse-phase nano column (BEH C18, 1.7 μm, 75 μm x 250 mm) at flow rate of 300 nL min^{-1}. Peptides were initially loaded onto a pre-column (Waters UPLC Trap Symmetry C18, 5 μm, 180 μm x 20 mm) from the nanoAcquity sample manager with 0.1% formic acid for 3 min at a flow rate of 10 μL min^{-1}. After this period, the column valve was switched to allow the elution of peptides from the pre-column onto the analytical column. Solvent A was water + 0.1% formic acid and solvent B was acetonitrile + 0.1% formic acid. The linear gradient employed was 5–40% B in 60 min. The LC eluent was sprayed into the mass spectrometer by means of a New Objective nanospray source. All *m/z* values of eluting ions were measured in the Orbitrap Velos mass analyser, set at a resolution of 30000. Data dependent scans (Top 20) were employed to automatically isolate and generate fragment ions by collision-induced dissociation in the linear ion trap, resulting in the generation of MS/MS spectra. Ions with charge states of 2+ and above were selected for fragmentation. Post-run, the data was processed using Protein Discoverer (version 1.3., ThermoFisher Scientific). Briefly, all MS/MS data were converted to mgf files and these were submitted to the Mascot search algorithm (Matrix Science, London UK) and searched against a custom database containing the corresponding protein and applying variable modifications of oxidation (M), deamination (NQ) and a custom modification (C), using a peptide tolerance of 25 ppm (MS) and 0.8 Da (MS/MS). Peptide identifications were accepted if they could be established at greater than 95.0% probability.

SDS-PAGE gel electrophoresis

SDS-PAGE gel electrophoresis was carried out using an XCell SureLock™ Mini-Cell Electrophoresis System from ThermoFisher Scientific (NuPAGE® Bis-Tris Mini Gels, NuPAGE® MES SDS running buffer). Protein molecular weights were approximated by comparison to a protein marker (Precision Plus Protein Standards 10–250 kDa from Bio-Rad). Briefly, a 10 μL aliquot of the reaction mixture was desalted with a Zeba™ Spin desalting column (ThermoFisher Scientific) and 5 μL of

this solution was transferred to a 0.5 eppendorf tube. NuPAGE® LDS sample buffer (4x, 2.5 μL), NuPAGE® reducing agent (10x, 1 μL), and H_2O (1.5 μL) were added. The solution was denatured at 70 °C for 10 min, loaded onto a 4–12% polyacrylamide NuPAGE® Bis-Tris mini gel, and then subjected to electrophoresis (200 V, 35 min) with 1x NuPAGE® MES SDS running buffer + NuPAGE® Antioxidant. Gels were visualised by coomassie staining (Instant Blue from Expedeon).

Circular Dichroism (CD)

CD measurements were performed on an Aviv Model 410 spectrometer, which was routinely calibrated with (*1S*)-(+)-10-camphorsulfonic acid. Spectra were recorded at 298K with a 0.1 cm quartz cell over the wavelength range 250–189 nm at 50 nm min^{-1}, with a bandwidth of 1.0 nm, the response time of 1 s, resolution step width of 1 nm and sensitivity of 20–50 Mdeg. Each spectrum represents the average of 5 scans. CD spectra were converted from raw ellipticity (θ, mdeg) to mean molar ellipticity per residue ([θ], deg cm^2 dmol^{-1} residue^{-1}).

Molecular Dynamics (MD) Simulations

MD simulations were performed using Amber12.[1] Parameters for the unnatural residues were generated by the *antechamber* module of Amber. A combination of ff14SB,[2] GLYCAM-06[3] (for the βGalS unit), and the general Amber force field (GAFF)[4] parameters were used to properly reproduce the conformational behaviour of the molecules. Partial charges were set to fit the electrostatic potential generated with HF/6-31G(d) by RESP.[5] The charges were calculated according to the Merz-Singh-Kollman scheme using Gaussian 09.[6] Each glycoprotein was immersed in a box with a 10 Å buffer of TIP3P[7] water molecules and neutralized by adding explicit counter ions (Na^+). A two-stage geometry optimization approach was used. The first stage minimizes only the positions of solvent molecules and ions, and the second stage is an unrestrained minimization of all the atoms in the simulation cell. The systems were then gently heated by incrementing the temperature from 0 to 300 K under a constant pressure of 1 atm and periodic boundary conditions. Harmonic restraints of 30 kcal/mol were applied to the solute, and the Andersen temperature coupling scheme[8] was used to control and equalize the temperature. The time step was kept at 1 fs during the heating stages. Long-range electrostatic effects were modelled using the particle-Mesh-Ewald method.[9] An 8-Å cut-off was applied to

Lennard-Jones and electrostatic interactions. Each system was equilibrated for 2 ns with a 2-fs time step at a constant volume and temperature of 300 K. Production trajectories were then run for additional 100 ns under the same simulation conditions.

Surface Plasmon Resonance (SPR)

Kinetic data analysis was performed using Biacore T100 Evaluation 1.1.1 software. Concentration ranges [0–135] nM. Materials, methods, and analysis were described previously.[10]

Biotinylation of Antibodies

Starting, modified **13**, and recovered Trastuzumab were conjugated to a biotin linker using Biotin-$(PEG)_4$-NHS (ThermoFisher Scientific) in order to carry out BLI experiments using Streptavidin (SA) Biosensors. A solution of EZ-Link NHS-(PEG)4-Biotin (20 μL, 200 μM in PBS) was added to the corresponding protein (20 μL, 20 μM in PBS) and was left at room temperature for 30 min. The crude reaction mixture was buffer exchanged with PBS for 3 times to remove the excess of NHS-$(PEG)_4$-Biotin, obtaining a biotin-to-antibody ratio around 1.6.

Bio-Layer Interferometry (BLI)

Binding assays were performed on an Octet Red Instrument (fortéBIO). Ligand immobilization, binding reactions, regeneration and washes were conducted in wells of black polypropylene 96-well microplates. Starting, modified **13**, and recovered Trastuzumab (20 nM) were immobilized on Streptavidin (SA) Biosensors in PBS with 0.1% BSA and 0.02% tween at 30 ºC. Binding analysis were carried out at 25 ºC, 1000 rpm in PBS with 0.1% BSA and 0.02% tween, with a 600 s of association followed by a 2200 s of dissociation, using different concentrations of recombinant HER2 receptor to obtain the association curve [0–200 nM]. Glycine pH 2.0 was used as a regeneration buffer. Data were analyzed using Data Analysis (fortéBIO), with Savitzky-Golay filtering. Binding was fitted to a 2:1 Heterogeneous ligand model, steady state analysis were performed to obtain the binding kinetics constants (K_D).

2. Experimental Section

2.1. Protein Modification

C2A Domain of Synaptotagmin-I (C2Am)[10]

Sequence of C2Am S95C (modified residue highlighted)

GSPGISGGGGGILDSMVEKLGKLQYSLDYDFQNNQLLVGIIQAAELPALDMG

GTSDPYVKVFLLPDKKKKFETKVHRKTLNPVFNEQFTFKVPYCELGGKTLVM

AVYD FDRFSKHDII GEFKVPMNTV DFGHVTEEWR DLQSAEK

Calculated average isotopic mass = 16222.53 Da (*N*-terminal Met cleaved)

A typical analysis of a conjugation reaction by LC–ESI–MS is described below. The total ion chromatogram, combined ion series, and deconvoluted spectra are shown for C2Am S95C. Identical analyses were carried out for all the conjugation reactions performed in this work.

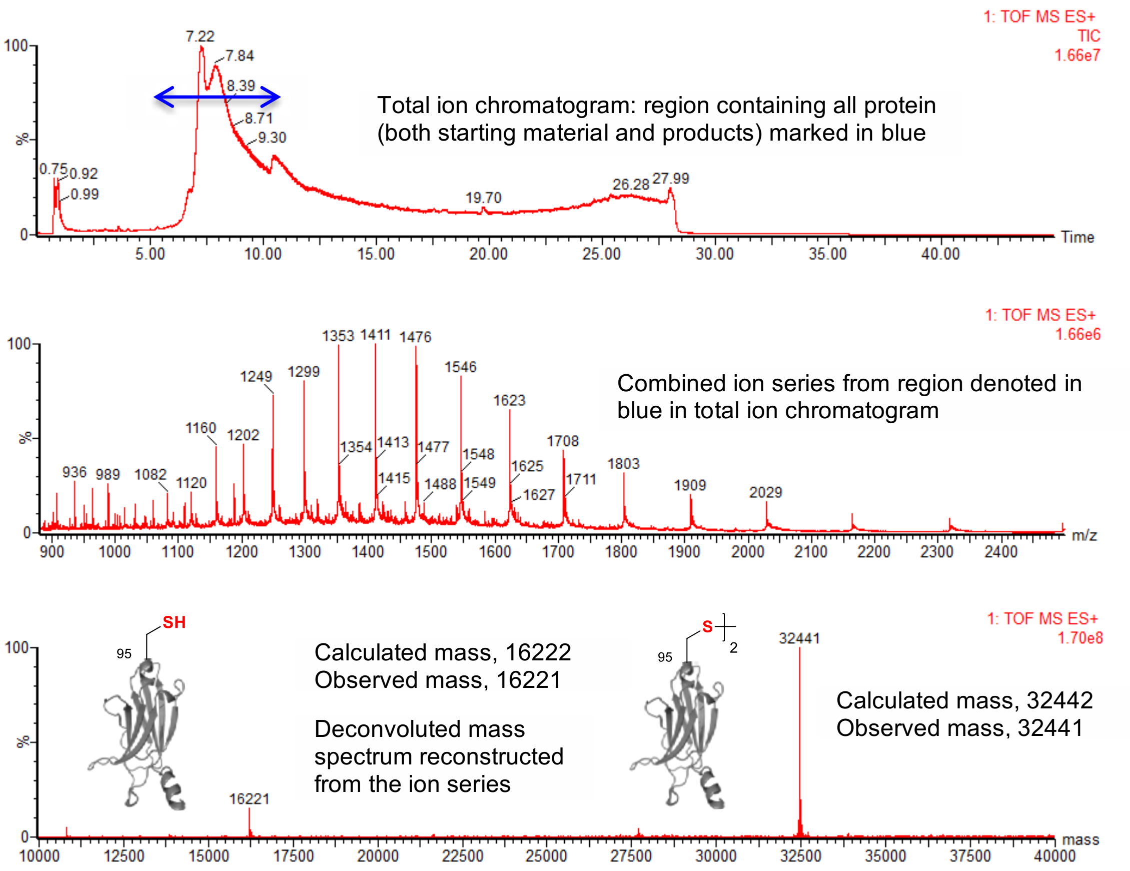

Figure S1. ESI–MS spectrum of non-reduced C2Am

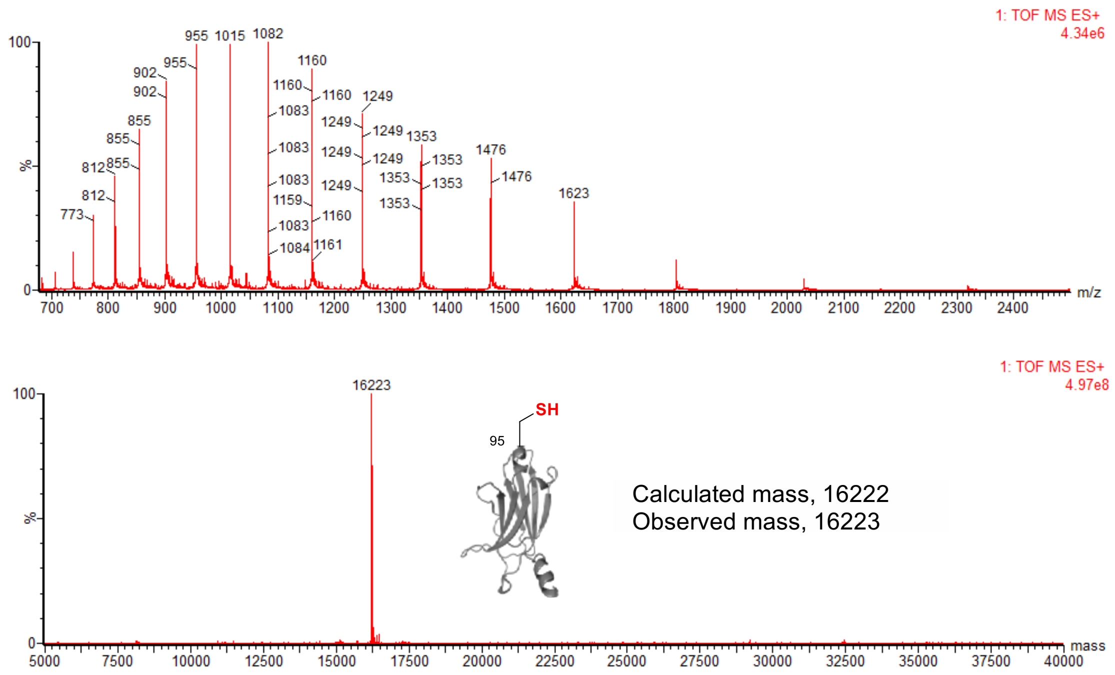

Figure S2. ESI–MS of reduced C2Am (with TCEP·HCl)

Control: reaction of C2Am with Ellman's reagent

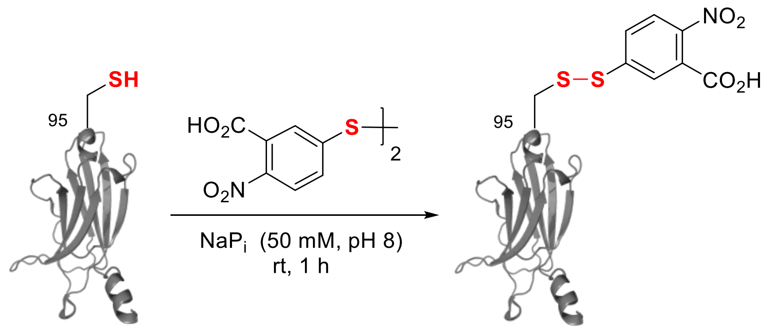

All manipulations were carried out at room temperature. Tris(2-carboxyethyl)phosphine hydrochloride (TCEP·HCl) (10 µL of a 1 mg/mL stock solution in H_2O, 308.2 nmol) was added to a solution of C2Am (50 µL, 1 mg/mL, 3.08 nmol) in 50 mM sodium phosphate buffer at pH 8 and the resulting mixture shaken for 2 h. Small molecules were removed from the reaction mixture by loading the sample onto a Zeba™ Spin desalting column (ThermoFisher Scientific) previously equilibrated with 50 mM sodium phosphate buffer at pH 8. The sample was eluted by centrifugation (2 min, 1500xg). A 10 µL aliquot of C2Am (*ca.* 1 mg/mL, 0.616 nmol) was transferred to a 0.5 mL eppendorf tube. Ellman's reagent (0.3 µL of a 20 mg/mL stock solution in H_2O, 15.14 nmol) was added and the resulting mixture vortexed for 30 seconds. After 1 h of additional shaking, a 3 µL aliquot was analyzed by LC–MS (3 µL aliquot diluted by 7 µL of 50 mM sodium

phosphate buffer at pH 8) and complete conversion to the expected Ellman's product (calculated mass, 16419; observed mass, 16419) was observed.

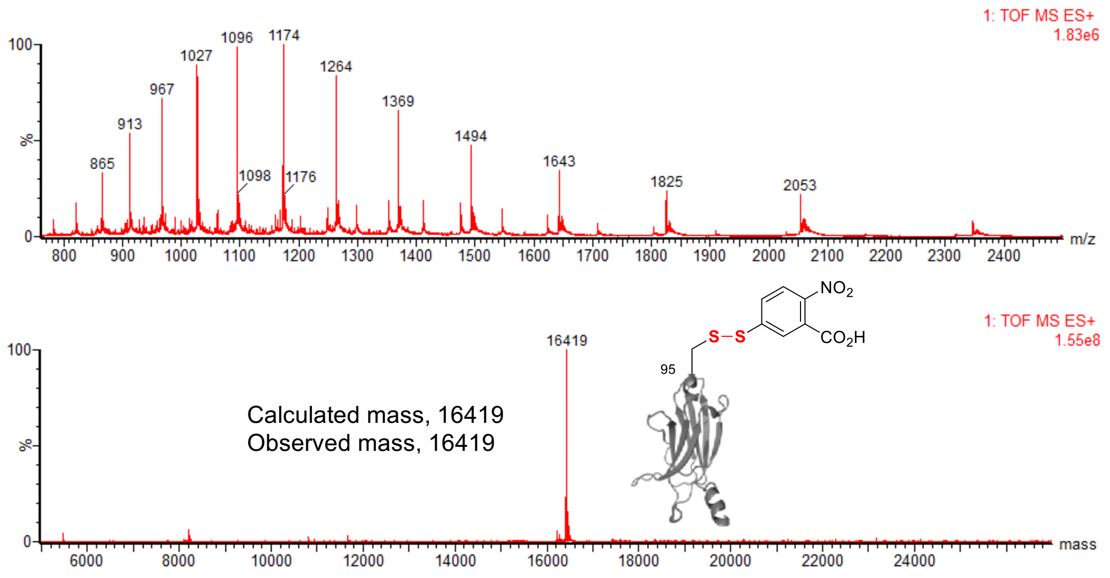

Figure S3. ESI–MS of the reaction of C2Am with Ellman's reagent

Reaction of C2Am with 3-bromooxetane (1)

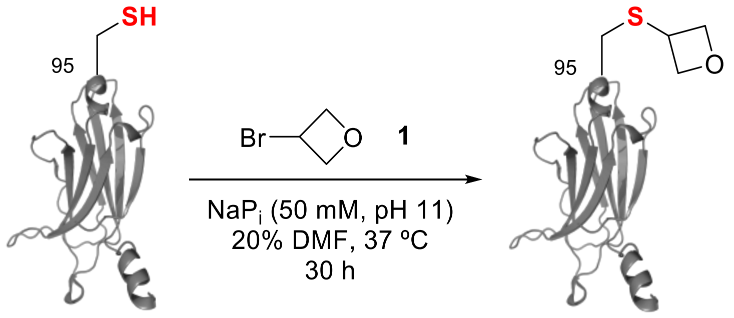

Tris(2-carboxyethyl)phosphine hydrochloride (TCEP·HCl) (0.12 mg, 416.1 nmol) was added to a solution of C2Am (540 µL, 0.25 mg/mL, 8.32 nmol) in 50 mM sodium phosphate buffer at pH 11 and the resulting mixture shaken at room temperature for 2 h. 3-Bromooxetane (**1**) (3.6 µL, 41.6 µmol) in DMF (135 µL) was added and the resulting mixture vortexed for 30 seconds. After 30 h of additional shaking at 37 ºC, a 3 µL aliquot was analyzed by LC–MS (3 µL aliquot diluted by 7 µL of 50 mM sodium phosphate buffer at pH 11) and complete conversion to C2Am-3-*S*-Ox (**2**) (calculated mass, 16278; observed mass, 16279) was observed. Small molecules were removed from the reaction mixture by loading the sample onto a Zeba™ Spin desalting column (ThermoFisher Scientific) previously equilibrated with PBS buffer at pH 7.6. The sample was eluted by centrifugation (2 min, 1500xg) and concentrated

on a Vivaspin™ membrane concentrator (10 kDa molecular weight cut off). The protein sample (2.77 mg/mL by Bradford assay) was flash frozen with liquid nitrogen and stored at –20 ºC.

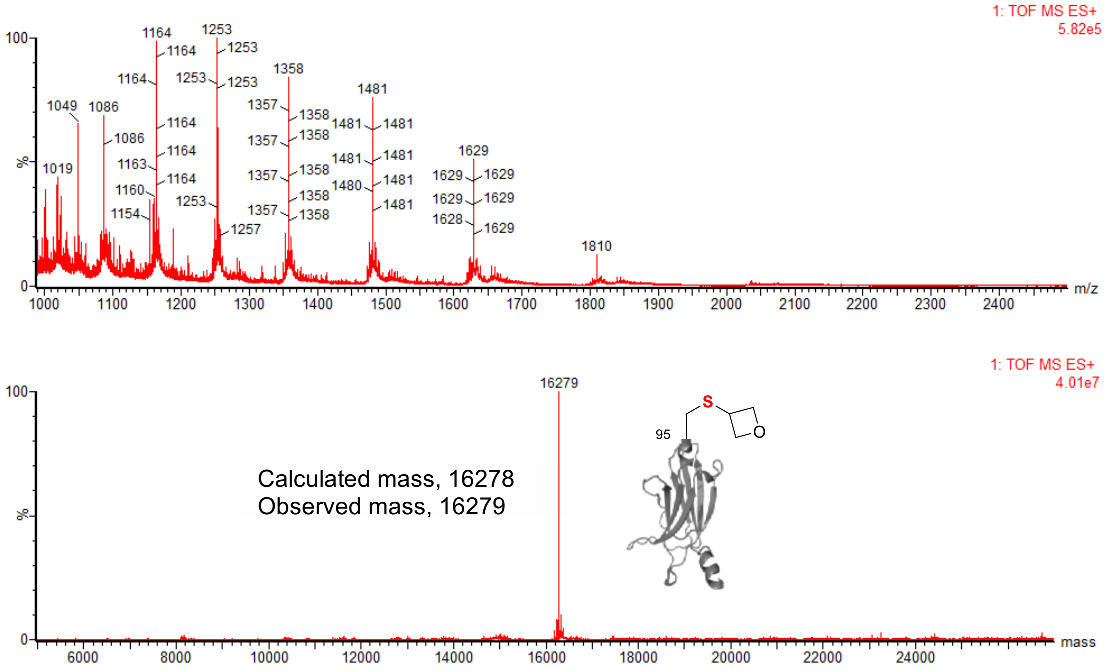

Figure S4. ESI–MS of the reaction of C2Am with 3-bromooxetane (**1**)

Control: incubation of C2Am-3-*S*-Ox (2) with Ellman's reagent

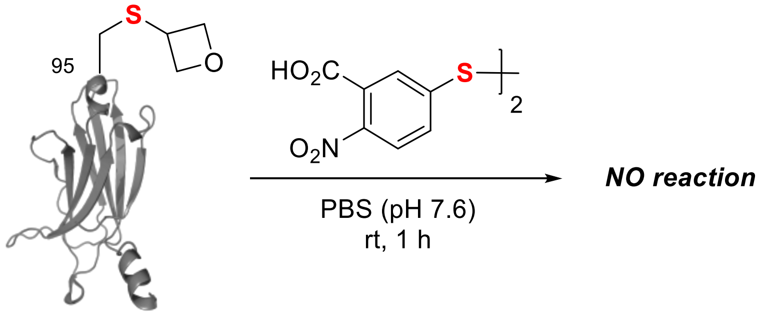

All manipulations were carried out at room temperature. A 10 µL aliquot of C2Am-3-*S*-Ox (**2**) (*ca.* 1 mg/mL, 0.614 nmol) in PBS buffer at pH 7.6 was transferred to a 0.5 mL eppendorf tube. Ellman's reagent (0.3 µL of a 20 mg/mL stock solution in H_2O, 15.14 nmol) was added and the resulting mixture vortexed for 30 seconds. After 1 h of additional shaking, a 3 µL aliquot was analyzed by LC–MS (3 µL aliquot diluted by 7 µL of PBS buffer at pH 7.6) and starting protein (**2**) (calculated mass, 16278; observed mass, 16279) was detected unaltered.

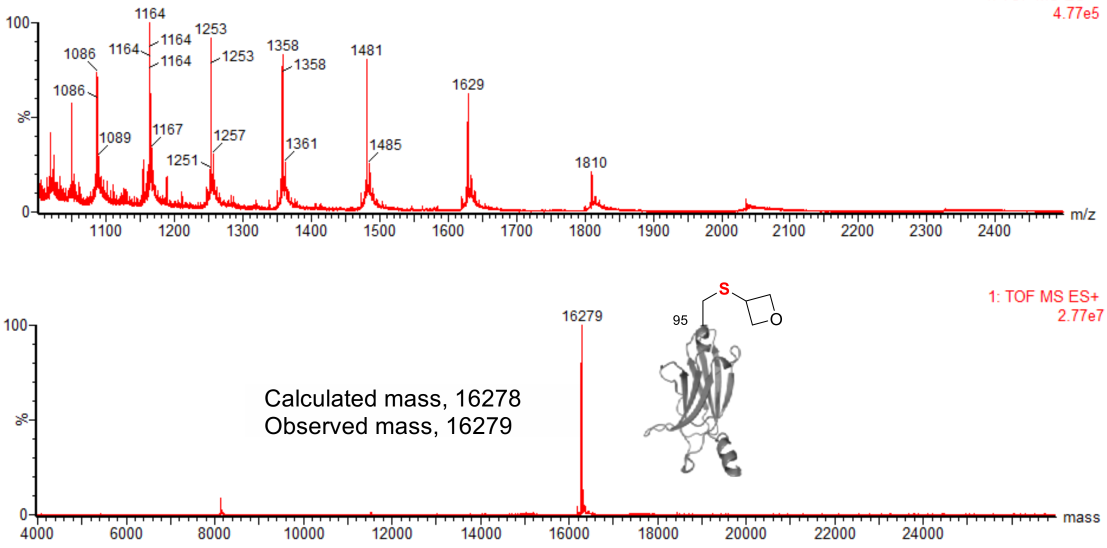

Figure S5. ESI–MS of the incubation of C2Am-3-*S*-Ox (**2**) with Ellman's reagent

Stability of C2Am-3-*S*-Ox (2) in human plasma

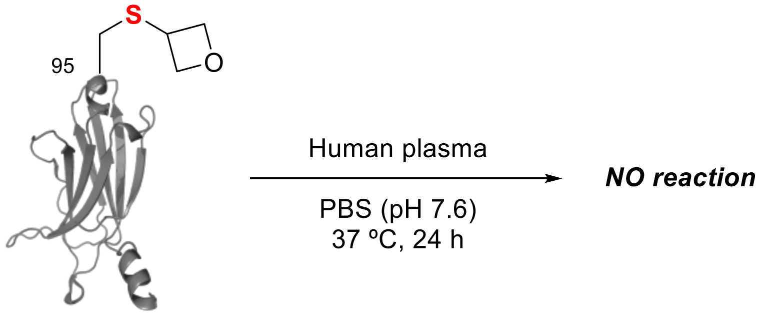

A 10 μL aliquot of C2Am-3-*S*-Ox (**2**) (*ca.* 1 mg/mL, 0.614 nmol) in PBS buffer at pH 7.6 was transferred to a 0.5 mL eppendorf tube. 1 μL of reconstituted human plasma (Sigma-Aldrich) was added at room temperature and the resulting mixture vortexed for 30 seconds. After 24 h of additional shaking at 37 ºC, a 2 μL aliquot was analyzed by LC–MS (2 μL aliquot diluted by 8 μL of PBS buffer at pH 7.6) and starting protein (**2**) (calculated mass, 16278; observed mass, 16279) was detected unaltered.

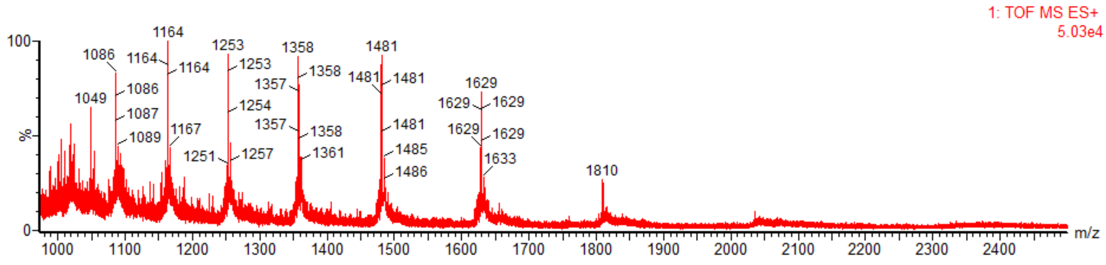

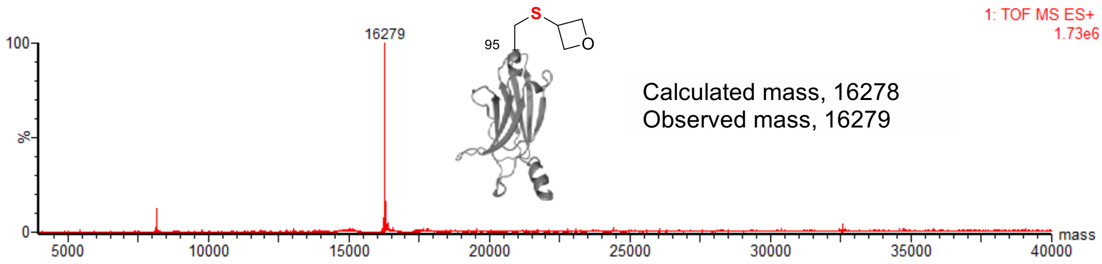

Figure S6. ESI–MS of the incubation of C2Am-3-*S*-Ox (**2**) with human plasma

Stability of C2Am-3-*S*-Ox (2) in the presence of reduced glutathione (GSH)

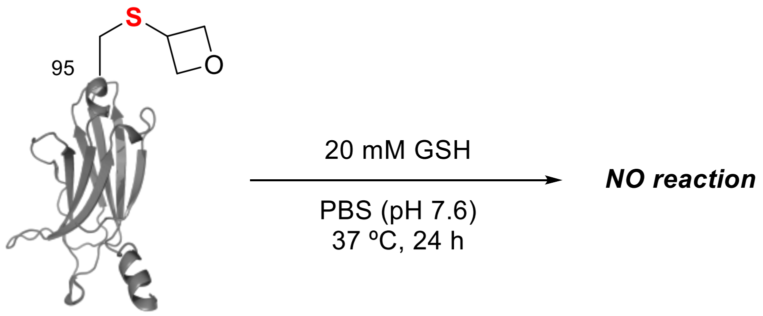

A 10 µL aliquot of C2Am-3-*S*-Ox (**2**) (*ca.* 1 mg/mL, 0.614 nmol) in PBS buffer at pH 7.6 was transferred to a 0.5 mL eppendorf tube. 1 µL of 20 mM GSH (6 mg in 1 mL of PBS buffer at pH 7.6) was added at room temperature and the resulting mixture vortexed for 30 seconds. After 24 h of additional shaking at 37 ºC, a 2 µL aliquot was analyzed by LC–MS (2 µL aliquot diluted by 8 µL of PBS buffer at pH 7.6) and starting protein (**2**) (calculated mass, 16278; observed mass, 16279) was detected unaltered.

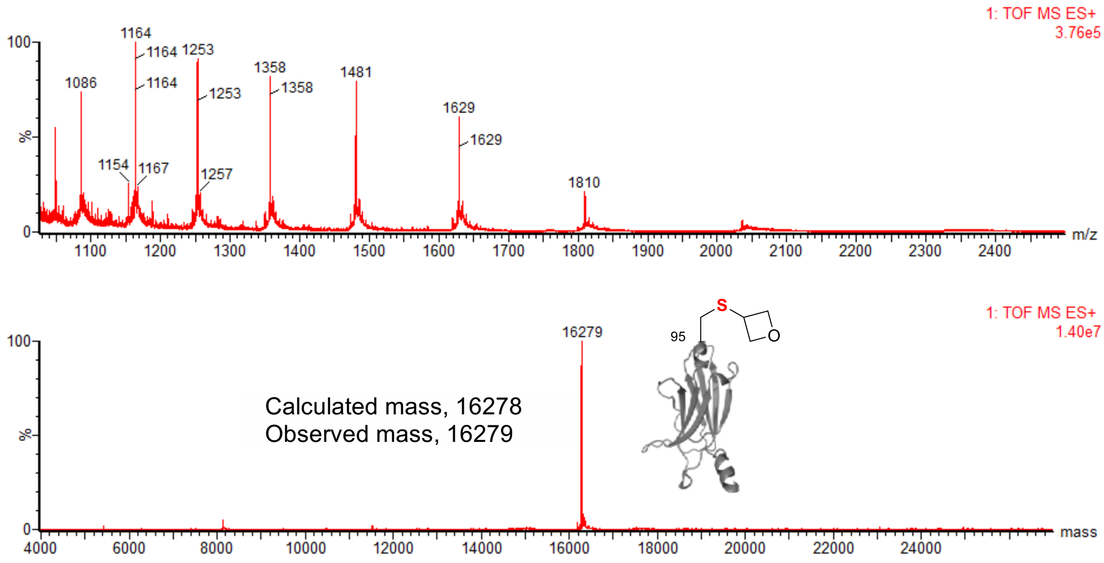

Figure S7. ESI–MS of the incubation of C2Am-3-*S*-Ox (**2**) with 20 mM GSH

Stability of C2Am-3-*S*-Ox (2) in the presence of β-mercaptoethanol (BME)

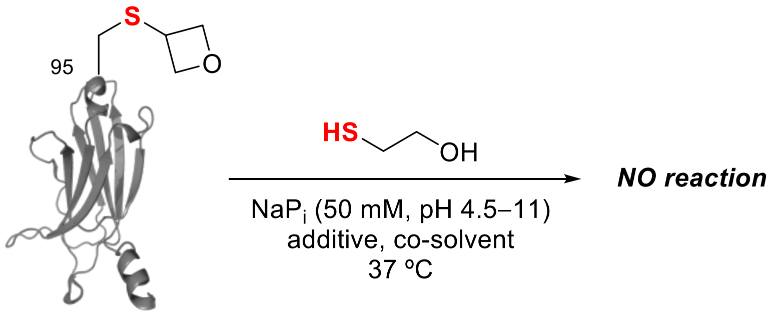

A 10 µL aliquot of C2Am-3-*S*-Ox (**2**) (*ca.* 0.25 mg/mL, 0.154 nmol) in 50 mM sodium phosphate buffer at pH 11 was transferred to a 0.5 mL eppendorf tube. BME (0.11 µL, 1535.8 nmol) was added at room temperature and the resulting mixture vortexed for 30 seconds. After 17 h of additional shaking at 37 ºC, a 2 µL aliquot was analyzed by LC–MS (2 µL aliquot diluted by 8 µL of 50 mM sodium phosphate buffer at pH 11) and starting protein (**2**) (calculated mass, 16278; observed mass, 16279) was detected unaltered.

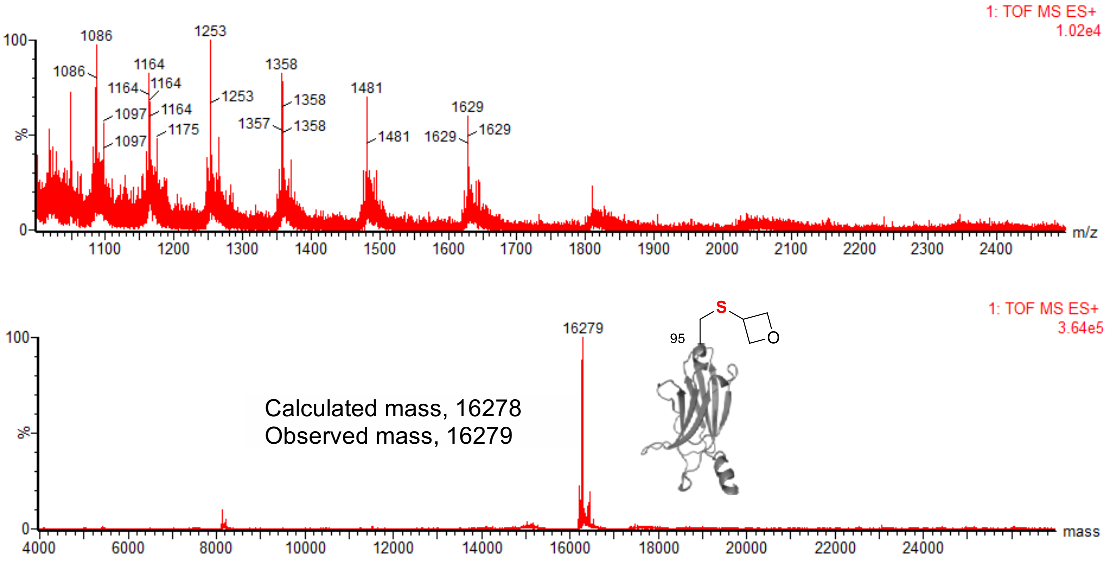

Figure S8. ESI–MS of the incubation of C2Am-3-*S*-Ox (**2**) with BME (pH 11)

A 10 µL aliquot of C2Am-3-*S*-Ox (**2**) (*ca.* 0.25 mg/mL, 0.154 nmol) in 50 mM sodium phosphate buffer at pH 11 was transferred to a 0.5 mL eppendorf tube. BME (0.11 µL, 1535.8 nmol) and $MgCl_2 \cdot 6H_2O$ (0.31 mg, 1535.8 nmol) were added at room temperature and the resulting mixture vortexed for 30 seconds. After 20 h of additional shaking at 37 ºC, a 2 µL aliquot was analyzed by LC–MS (2 µL aliquot

diluted by 8 μL of 50 mM sodium phosphate buffer at pH 11) and starting protein (**2**) (calculated mass, 16278; observed mass, 16279) was detected unaltered.

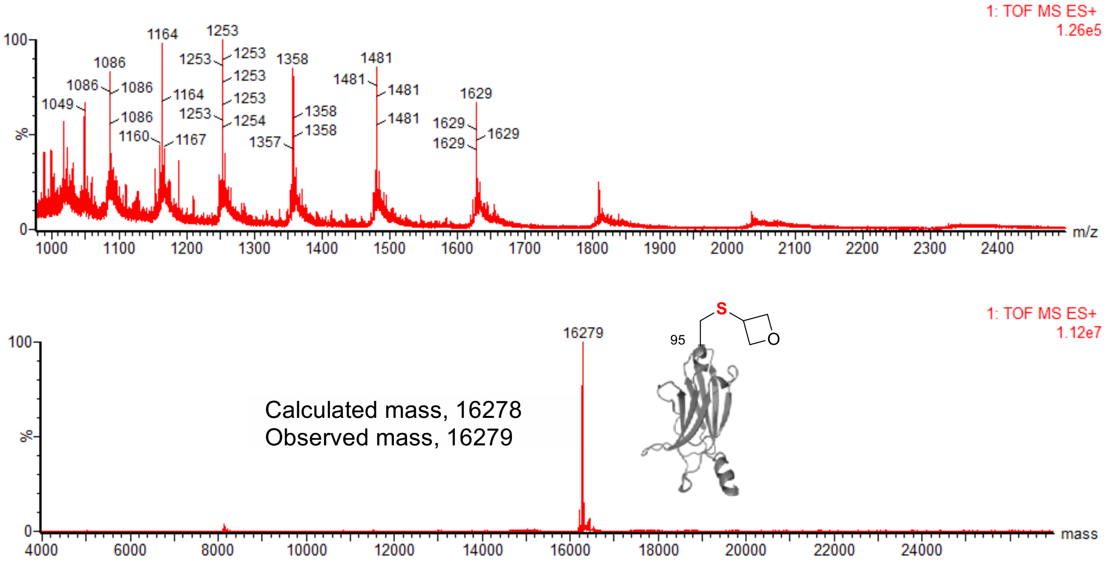

Figure S9. ESI–MS of the incubation of C2Am-3-*S*-Ox (**2**) with BME and $MgCl_2 \cdot 6H_2O$ (pH 11)

A 10 μL aliquot of C2Am-3-*S*-Ox (**2**) (*ca.* 0.25 mg/mL, 0.154 nmol) in 50 mM sodium phosphate buffer at pH 11 was transferred to a 0.5 mL eppendorf tube. BME (0.11 μL, 1535.8 nmol) and $Y(OTf)_3$ (0.95 mg, 1535.8 nmol) were added at room temperature and the resulting mixture vortexed for 30 seconds. After 20 h of additional shaking at 37 ºC, a 2 μL aliquot was analyzed by LC–MS (2 μL aliquot diluted by 8 μL of 50 mM sodium phosphate buffer at pH 11) and starting protein (**2**) (calculated mass, 16278; observed mass, 16279) was detected unaltered.

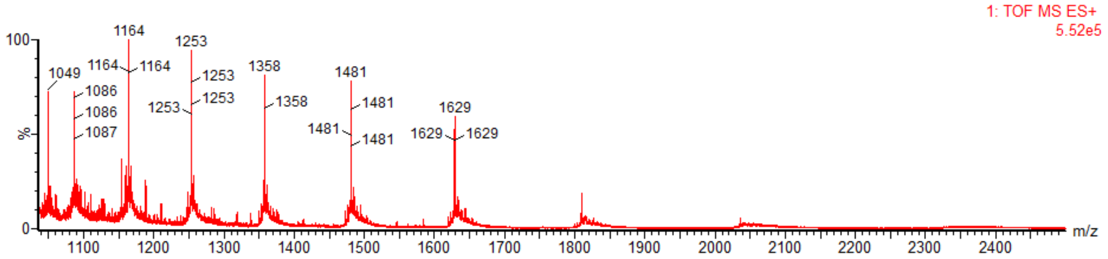

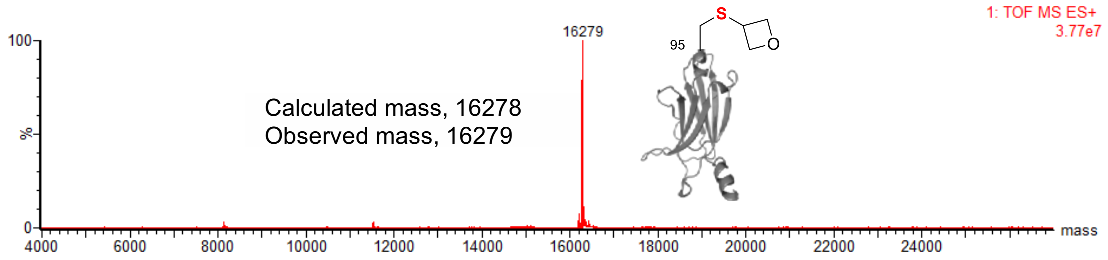

Figure S10. ESI–MS of the incubation of C2Am-3-*S*-Ox (**2**) with BME and $Y(OTf)_3$ (pH 11)

A 10 µL aliquot of C2Am-3-*S*-Ox (**2**) (*ca.* 0.25 mg/mL, 0.154 nmol) in 50 mM sodium phosphate buffer at pH 4.5 was transferred to a 0.5 mL eppendorf tube. BME (0.11 µL, 1535.8 nmol), $MgCl_2{\cdot}6H_2O$ (0.31 mg, 1535.8 nmol), and CH_3CN (2.5 µL) were added at room temperature and the resulting mixture vortexed for 30 seconds. After 24 h of additional shaking at 37 ºC, a 2 µL aliquot was analyzed by LC–MS (2 µL aliquot diluted by 8 µL of 50 mM sodium phosphate buffer at pH 4.5) and starting protein (**2**) (calculated mass, 16278; observed mass, 16279) was detected unaltered.

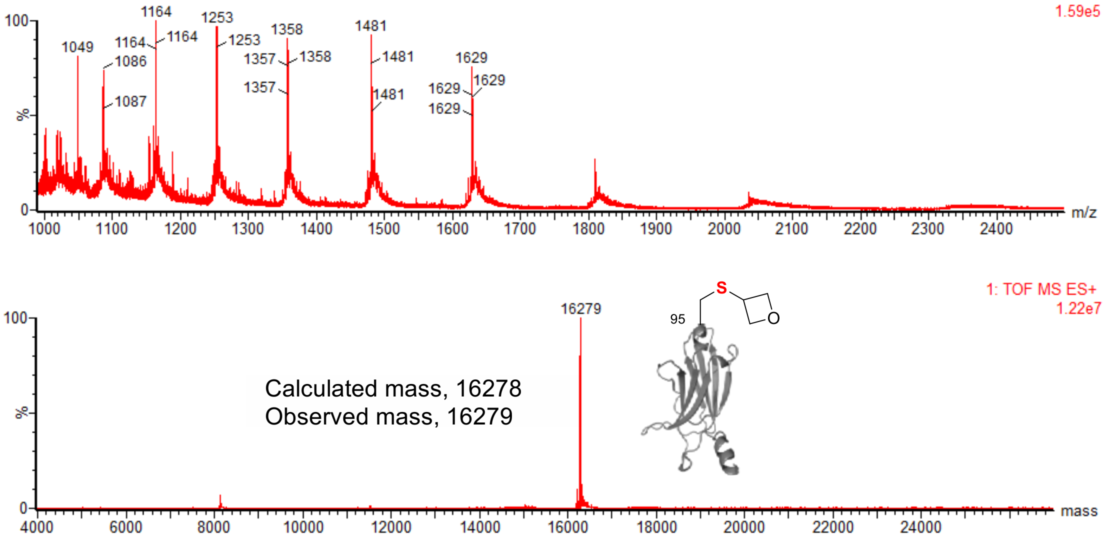

Figure S11. ESI–MS of the incubation of C2Am-3-*S*-Ox (**2**) with BME, $MgCl_2{\cdot}6H_2O$, and 20% CH_3CN (pH 4.5)

A 10 µL aliquot of C2Am-3-*S*-Ox (**2**) (*ca.* 0.25 mg/mL, 0.154 nmol) in 50 mM sodium phosphate buffer at pH 4.5 was transferred to a 0.5 mL eppendorf tube. BME (0.11 µL, 1535.8 nmol), $Y(OTf)_3$ (0.95 mg, 1535.8 nmol), and CH_3CN (2.5 µL) were added at room temperature and the resulting mixture vortexed for 30 seconds. After 24 h of additional shaking at 37 ºC, a 2 µL aliquot was analyzed by LC–MS (2 µL

aliquot diluted by 8 µL of 50 mM sodium phosphate buffer at pH 4.5) and starting protein (**2**) (calculated mass, 16278; observed mass, 16279) was detected unaltered.

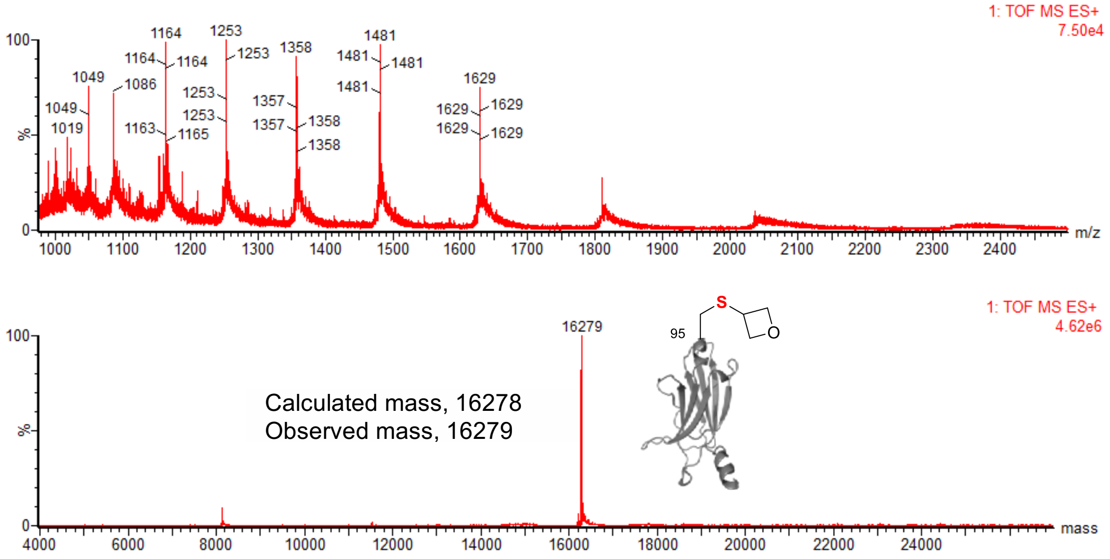

Figure S12. ESI–MS of the incubation of C2Am-3-*S*-Ox (**2**) with BME, $Y(OTf)_3$, and 20% CH_3CN (pH 4.5)

Stability of C2Am-3-*S*-Ox (2) in the presence of thiophenol (PhSH)

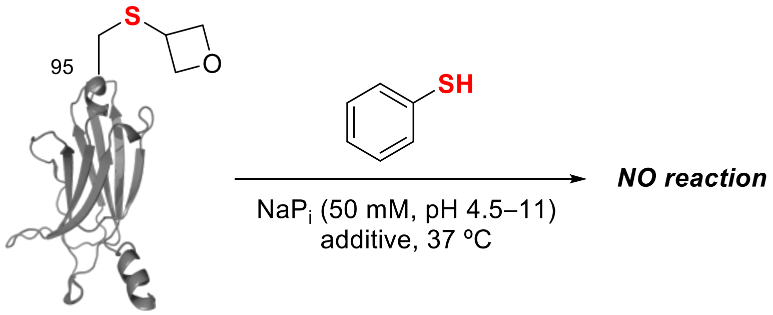

A 10 µL aliquot of C2Am-3-*S*-Ox (**2**) (*ca.* 0.25 mg/mL, 0.154 nmol) in 50 mM sodium phosphate buffer at pH 11 was transferred to a 0.5 mL eppendorf tube. PhSH (0.16 µL, 1535.8 nmol) was added at room temperature and the resulting mixture vortexed for 30 seconds. After 5 h of additional shaking at 37 ºC, a 2 µL aliquot was analyzed by LC–MS (2 µL aliquot diluted by 8 µL of 50 mM sodium phosphate buffer at pH 11) and starting protein (**2**) (calculated mass, 16278; observed mass, 16279) was detected unaltered.

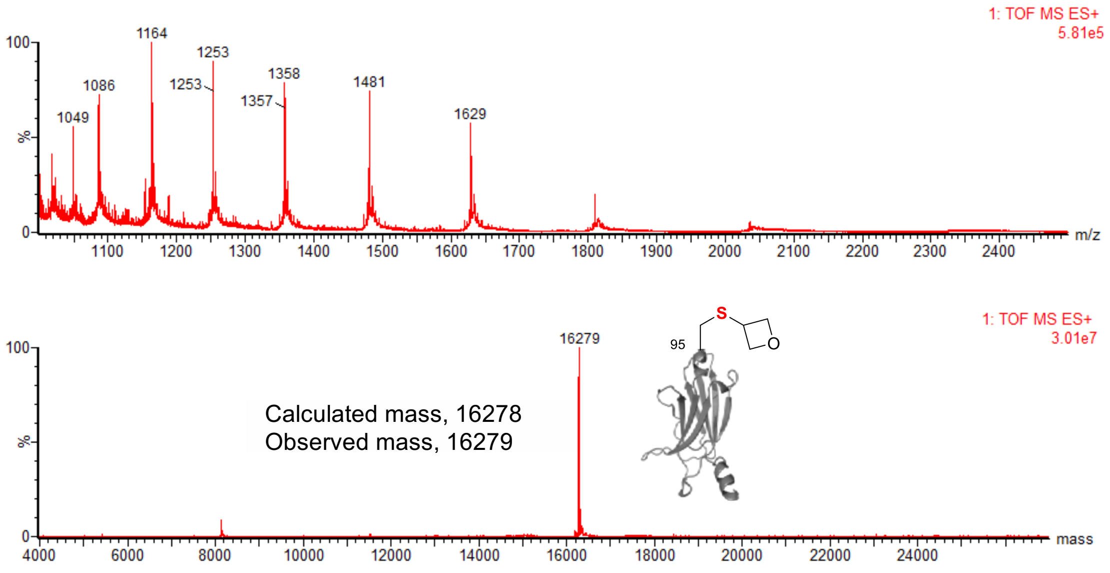

Figure S13. ESI–MS of the incubation of C2Am-3-*S*-Ox (**2**) with PhSH (pH 11)

A 10 μL aliquot of C2Am-3-*S*-Ox (**2**) (*ca.* 0.25 mg/mL, 0.154 nmol) in 50 mM sodium phosphate buffer at pH 11 was transferred to a 0.5 mL eppendorf tube. PhSH (0.16 μL, 1535.8 nmol) and $MgCl_2 \cdot 6H_2O$ (0.31 mg, 1535.8 nmol) were added at room temperature and the resulting mixture vortexed for 30 seconds. After 20 h of additional shaking at 37 ºC, a 2 μL aliquot was analyzed by LC–MS (2 μL aliquot diluted by 8 μL of 50 mM sodium phosphate buffer at pH 11) and starting protein (**2**) (calculated mass, 16278; observed mass, 16279) was detected unaltered.

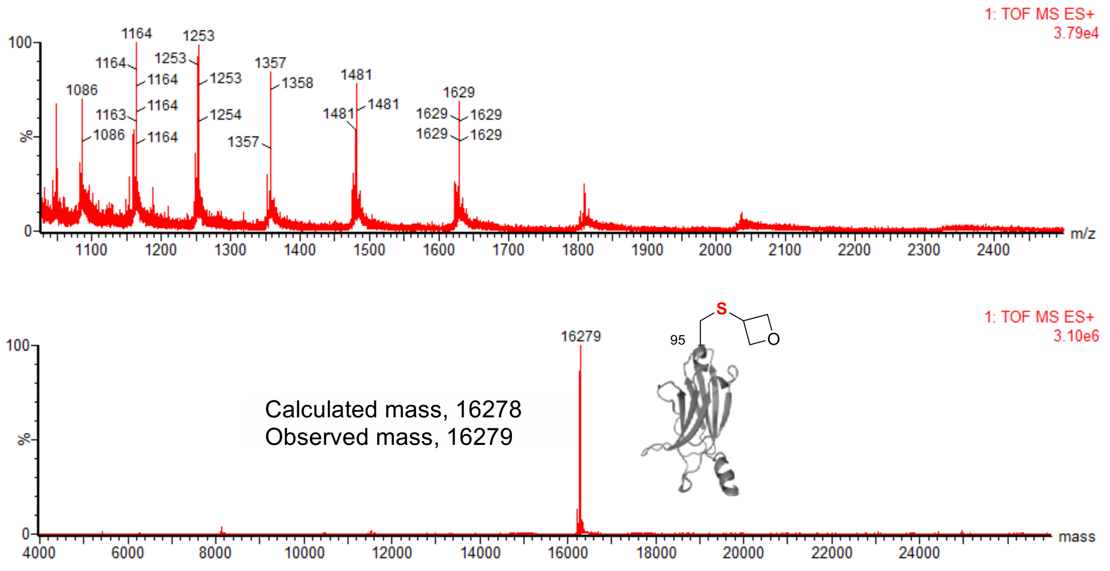

Figure S14. ESI–MS of the incubation of C2Am-3-*S*-Ox (**2**) with PhSH and $MgCl_2 \cdot 6H_2O$ (pH 11)

A 10 μL aliquot of C2Am-3-*S*-Ox (**2**) (*ca.* 0.25 mg/mL, 0.154 nmol) in 50 mM sodium phosphate buffer at pH 11 was transferred to a 0.5 mL eppendorf tube. PhSH (0.16 μL, 1535.8 nmol) and $Y(OTf)_3$ (0.95 mg, 1535.8 nmol) were added at room temperature and the resulting mixture vortexed for 30 seconds. After 20 h of additional shaking at 37 ºC, a 2 μL aliquot was analyzed by LC–MS (2 μL aliquot diluted by 8 μL of 50 mM sodium phosphate buffer at pH 11) and starting protein (**2**) (calculated mass, 16278; observed mass, 16279) was detected unaltered.

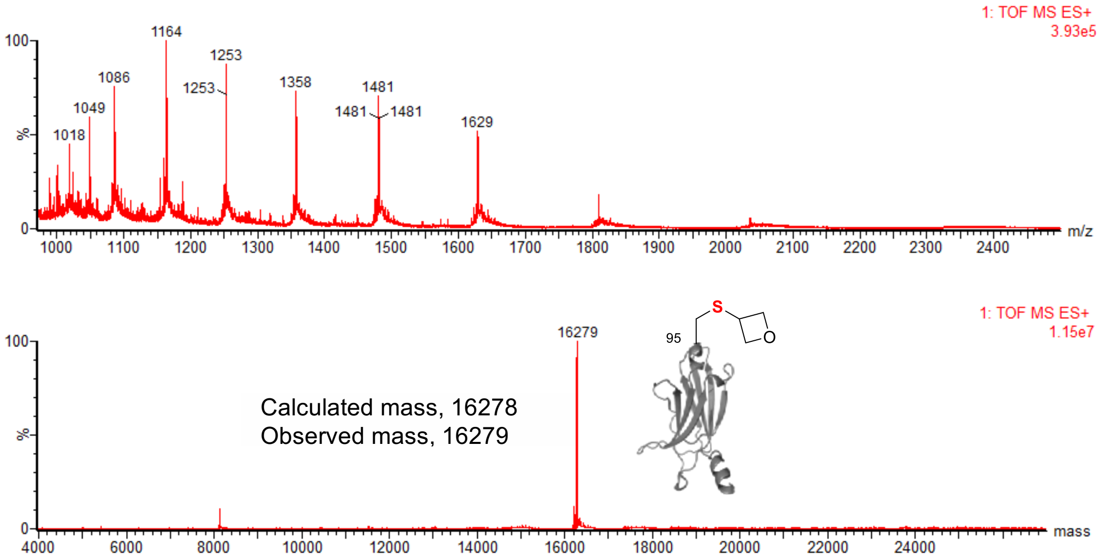

Figure S15. ESI–MS of the incubation of C2Am-3-*S*-Ox (**2**) with PhSH and $Y(OTf)_3$ (pH 11)

A 10 μL aliquot of C2Am-3-*S*-Ox (**2**) (*ca.* 0.25 mg/mL, 0.154 nmol) in 50 mM sodium phosphate buffer at pH 4.5 was transferred to a 0.5 mL eppendorf tube. PhSH (0.16 μL, 1535.8 nmol) and urea (0.18 mg, 3071.6 nmol) were added at room temperature and the resulting mixture vortexed for 30 seconds. After 22 h of additional shaking at 37 ºC, a 2 μL aliquot was analyzed by LC–MS (2 μL aliquot diluted by 8 μL of 50 mM sodium phosphate buffer at pH 4.5) and starting protein (**2**) (calculated mass, 16278; observed mass, 16279) was detected unaltered.

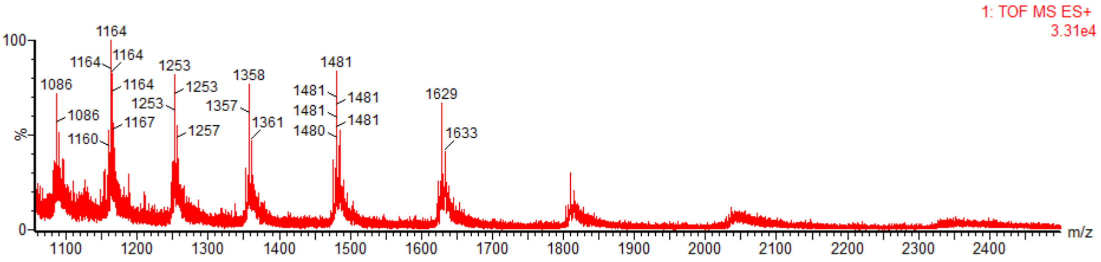

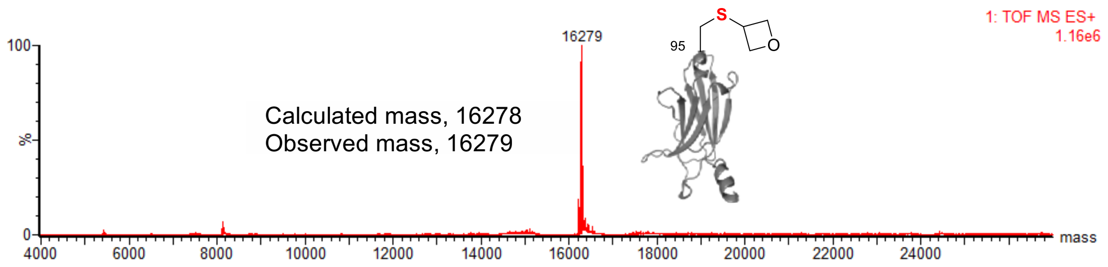

Figure S16. ESI–MS of the incubation of C2Am-3-*S*-Ox (**2**) with PhSH and urea (pH 4.5)

A 10 µL aliquot of C2Am-3-*S*-Ox (**2**) (*ca.* 0.25 mg/mL, 0.154 nmol) in 50 mM sodium phosphate buffer at pH 4.5 was transferred to a 0.5 mL eppendorf tube. PhSH (0.16 µL, 1535.8 nmol) and *thio*urea (0.12 mg, 3071.6 nmol) were added at room temperature and the resulting mixture vortexed for 30 seconds. After 22 h of additional shaking at 37 ºC, a 2 µL aliquot was analyzed by LC–MS (2 µL aliquot diluted by 8 µL of 50 mM sodium phosphate buffer at pH 4.5) and starting protein (**2**) (calculated mass, 16278; observed mass, 16279) was detected unaltered.

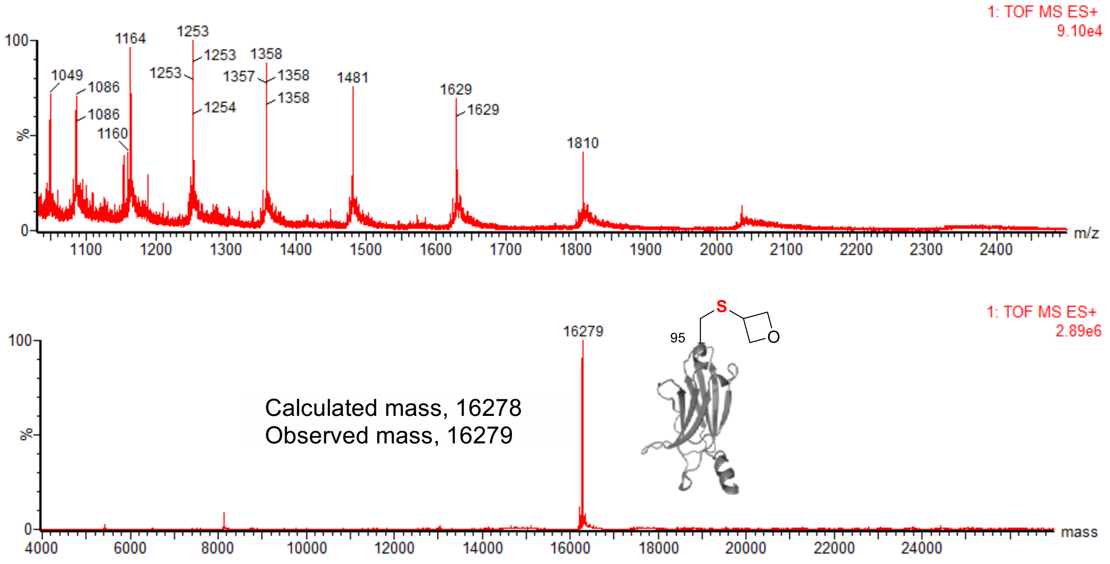

Figure S17. ESI–MS of the incubation of C2Am-3-*S*-Ox (**2**) with PhSH and *thio*urea (pH 4.5)

Stability of C2Am-3-*S*-Ox (2) in the presence of βGalSNa

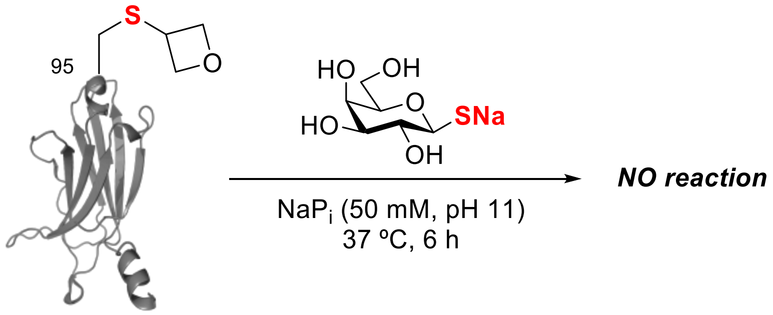

A 10 μL aliquot of C2Am-3-*S*-Ox (**2**) (*ca.* 0.25 mg/mL, 0.154 nmol) in 50 mM sodium phosphate buffer at pH 11 was transferred to a 0.5 mL eppendorf tube. βGalSNa (0.30 mg, 1535.8 nmol) was added at room temperature and the resulting mixture vortexed for 30 seconds. After 6 h of additional shaking at 37 ºC, a 2 μL aliquot was analyzed by LC–MS (2 μL aliquot diluted by 8 μL of 50 mM sodium phosphate buffer at pH 11) and starting protein (**2**) (calculated mass, 16278; observed mass, 16279) was detected unaltered.

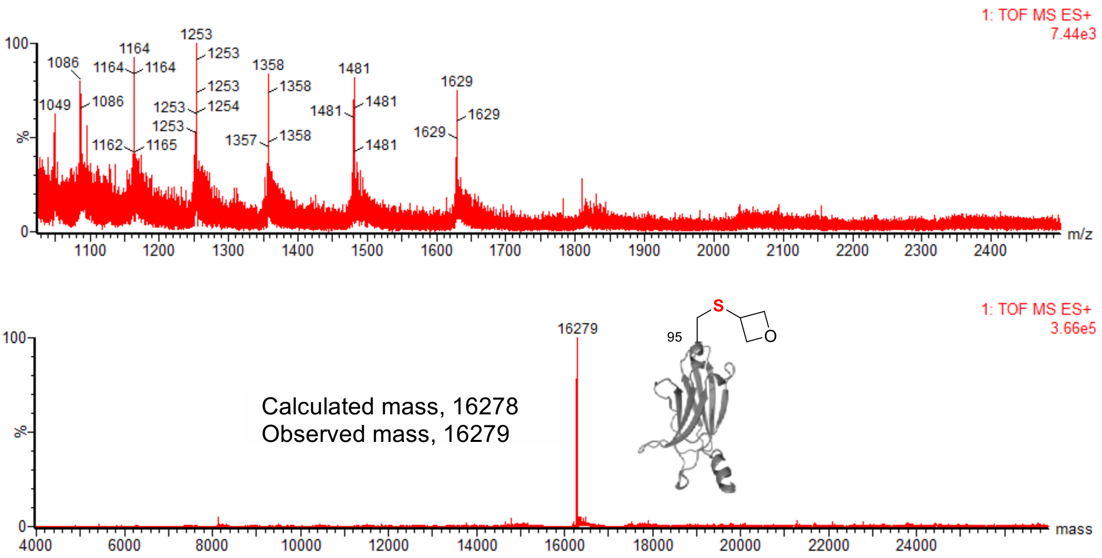

Figure S18. ESI–MS of the incubation of C2Am-3-*S*-Ox (**2**) with βGalSNa

Stability of C2Am-3-*S*-Ox (2) in the presence of benzylamine ($BnNH_2$)

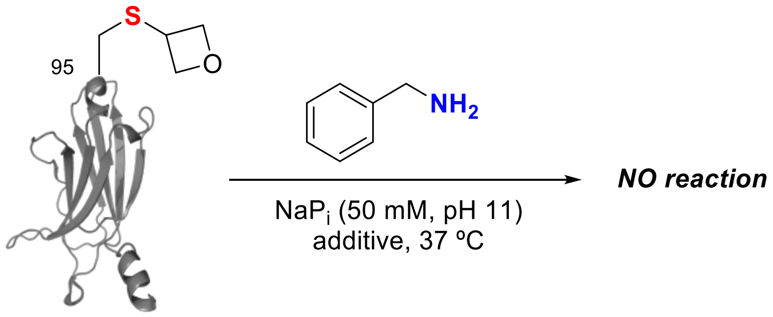

A 10 µL aliquot of C2Am-3-*S*-Ox (**2**) (*ca.* 0.25 mg/mL, 0.154 nmol) in 50 mM sodium phosphate buffer at pH 11 was transferred to a 0.5 mL eppendorf tube. $BnNH_2$ (0.17 µL, 1535.8 nmol) was added at room temperature and the resulting mixture vortexed for 30 seconds. After 16 h of additional shaking at 37 ºC, a 2 µL aliquot was analyzed by LC–MS (2 µL aliquot diluted by 8 µL of 50 mM sodium phosphate buffer at pH 11) and starting protein (**2**) (calculated mass, 16278; observed mass, 16279) was detected unaltered.

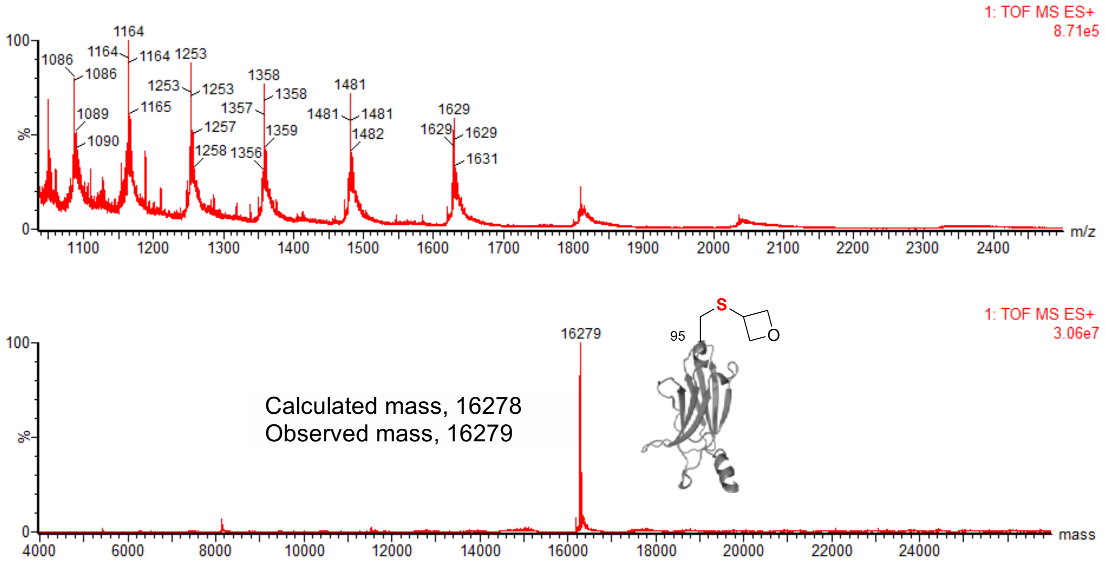

Figure S19. ESI–MS of the incubation of C2Am-3-*S*-Ox (**2**) with $BnNH_2$

A 10 µL aliquot of C2Am-3-*S*-Ox (**2**) (*ca.* 0.25 mg/mL, 0.154 nmol) in 50 mM sodium phosphate buffer at pH 11 was transferred to a 0.5 mL eppendorf tube. $BnNH_2$ (0.17 µL, 1535.8 nmol) and $MgCl_2 \cdot 6H_2O$ (0.31 mg, 1535.8 nmol) were added at room temperature and the resulting mixture vortexed for 30 seconds. After 3 h of additional shaking at 37 ºC, a 2 µL aliquot was analyzed by LC–MS (2 µL aliquot

diluted by 8 μL of 50 mM sodium phosphate buffer at pH 11) and starting protein (**2**) (calculated mass, 16278; observed mass, 16280) was detected unaltered.

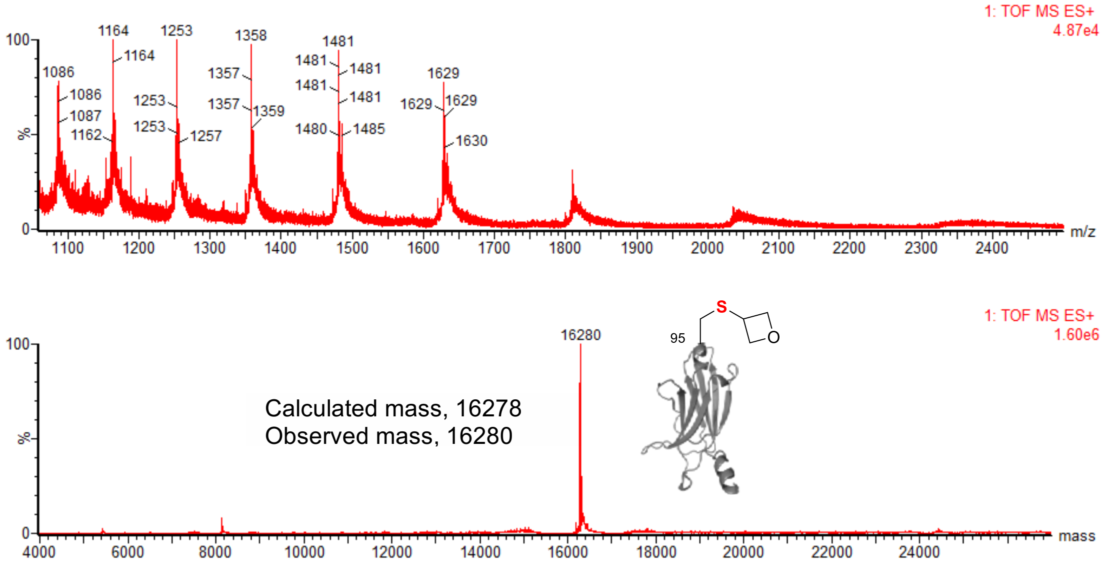

Figure S20. ESI–MS of the incubation of C2Am-3-*S*-Ox (**2**) with $BnNH_2$ and $MgCl_2{\cdot}6H_2O$

A 10 μL aliquot of C2Am-3-*S*-Ox (**2**) (*ca.* 0.25 mg/mL, 0.154 nmol) in 50 mM sodium phosphate buffer at pH 11 was transferred to a 0.5 mL eppendorf tube. $BnNH_2$ (0.17 μL, 1535.8 nmol) and $Y(OTf)_3$ (0.95 mg, 1535.8 nmol) were added at room temperature and the resulting mixture vortexed for 30 seconds. After 4 h of additional shaking at 37 ºC, a 2 μL aliquot was analyzed by LC–MS (2 μL aliquot diluted by 8 μL of 50 mM sodium phosphate buffer at pH 11) and starting protein (**2**) (calculated mass, 16278; observed mass, 16279) was detected unaltered.

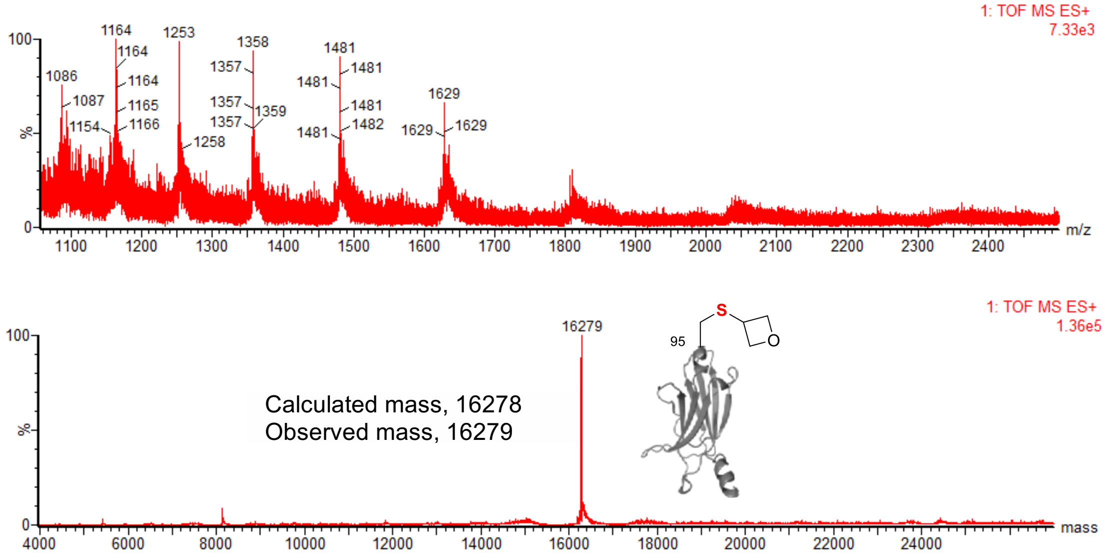

Figure S21. ESI–MS of the incubation of C2Am-3-*S*-Ox (**2**) with $BnNH_2$ and $Y(OTf)_3$

Reaction of C2Am with 3,3-bis(bromomethyl)oxetane (4)

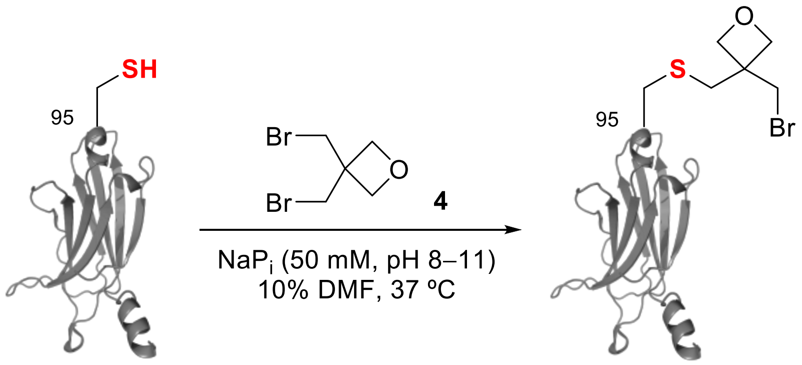

Tris(2-carboxyethyl)phosphine hydrochloride (TCEP·HCl) (2 μL of an 11 mg/mL stock solution in H_2O, 678.0 nmol) was added to a solution of C2Am (25 μL, 1 mg/mL, 1.54 nmol) in 50 mM sodium phosphate buffer at pH 8 and the resulting mixture shaken at room temperature for 30 min. 3,3-Bis(bromomethyl)oxetane (**4**) (0.59 mg, 2.31 μmol) in DMF (3 μL) was added and the resulting mixture vortexed for 30 seconds. After 5 h of additional shaking at 37 ºC, a 3 μL aliquot was analyzed by LC–MS (3 μL aliquot diluted by 7 μL of 50 mM sodium phosphate buffer at pH 8) and complete conversion to C2Am-BrCH_2-*S*-Ox (**5**) (calculated mass, 16385; observed mass, 16386) was observed.

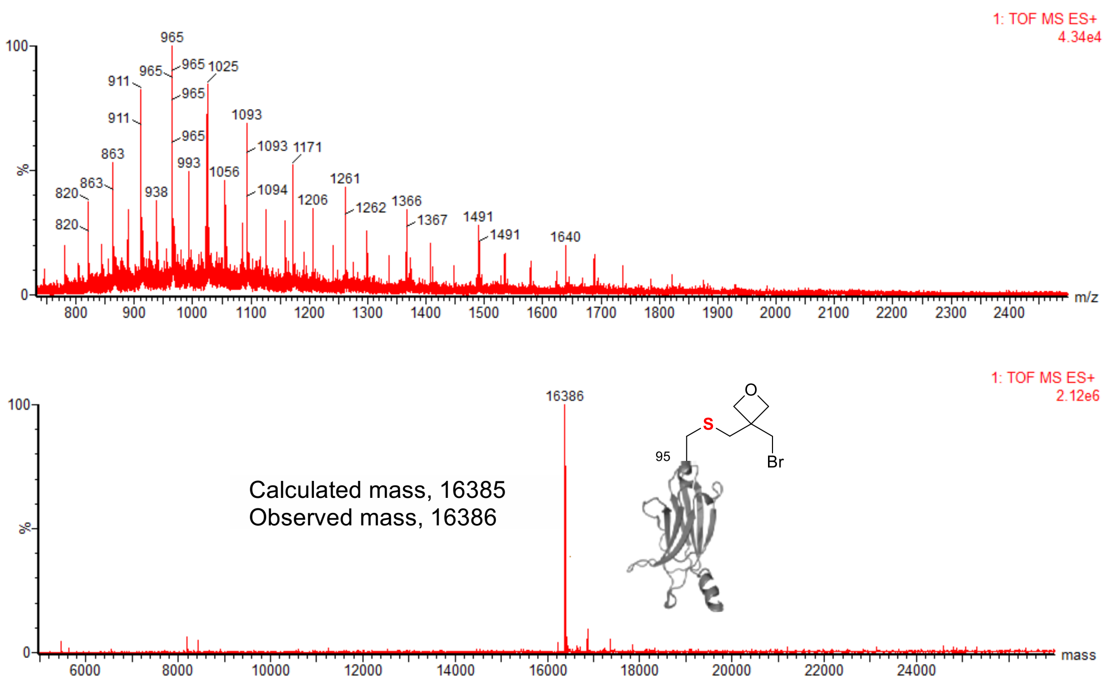

Figure S22. ESI–MS of the reaction of C2Am with 3,3-bis(bromomethyl)oxetane (**4**) (pH 8)

Tris(2-carboxyethyl)phosphine hydrochloride (TCEP·HCl) (2 µL of a 44 mg/mL stock solution in H_2O, 27.1 µmol) was added to a solution of C2Am (100 µL, 1 mg/mL, 6.16 nmol) in 50 mM sodium phosphate buffer at pH 11 and the resulting mixture shaken at room temperature for 45 min. 3,3-Bis(bromomethyl)oxetane (**4**) (2.4 mg, 9.25 µmol) in DMF (12.4 µL) was added and the resulting mixture vortexed for 30 seconds. After 2 h of additional shaking at 37 ºC, a 2 µL aliquot was analyzed by LC–MS (2 µL aliquot diluted by 8 µL of 50 mM sodium phosphate buffer at pH 11) and complete conversion to C2Am-BrCH$_2$-*S*-Ox (**5**) (calculated mass, 16385; observed mass, 16386) was observed. Small molecules were removed from the reaction mixture by loading the sample onto a Zeba™ Spin desalting column (ThermoFisher Scientific) previously equilibrated with PBS buffer at pH 7.6. The sample was eluted by centrifugation (2 min, 1500xg). The protein sample (1 mg/mL by Bradford assay) was flash frozen with liquid nitrogen and stored at –20 ºC.

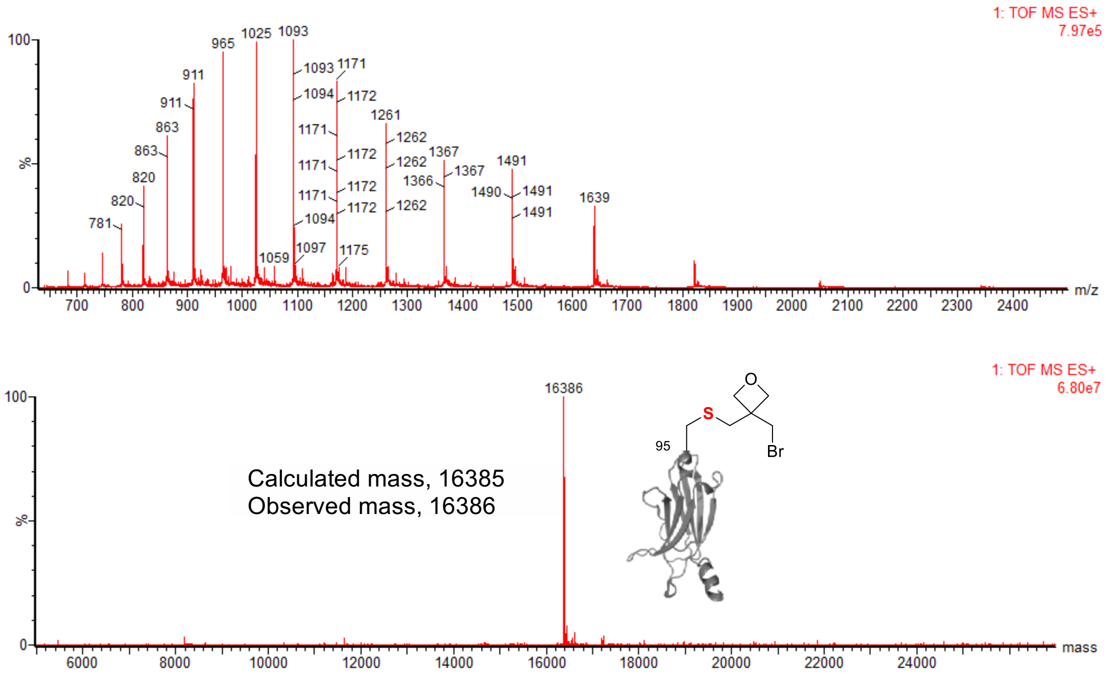

Figure S23. ESI–MS of the reaction of C2Am with 3,3-bis(bromomethyl)oxetane (**4**) (pH 11)

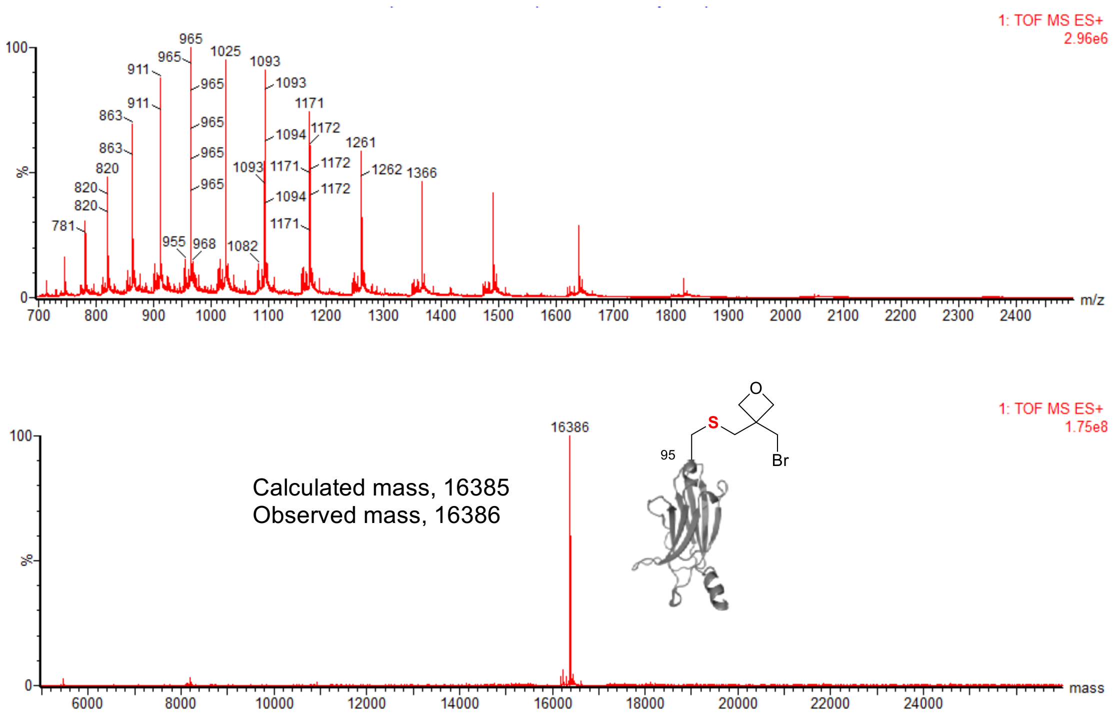

Figure S24. ESI–MS of C2Am-BrCH$_2$-*S*-Ox (**5**) after incubation at 37 ºC for 8 h (pH 11)

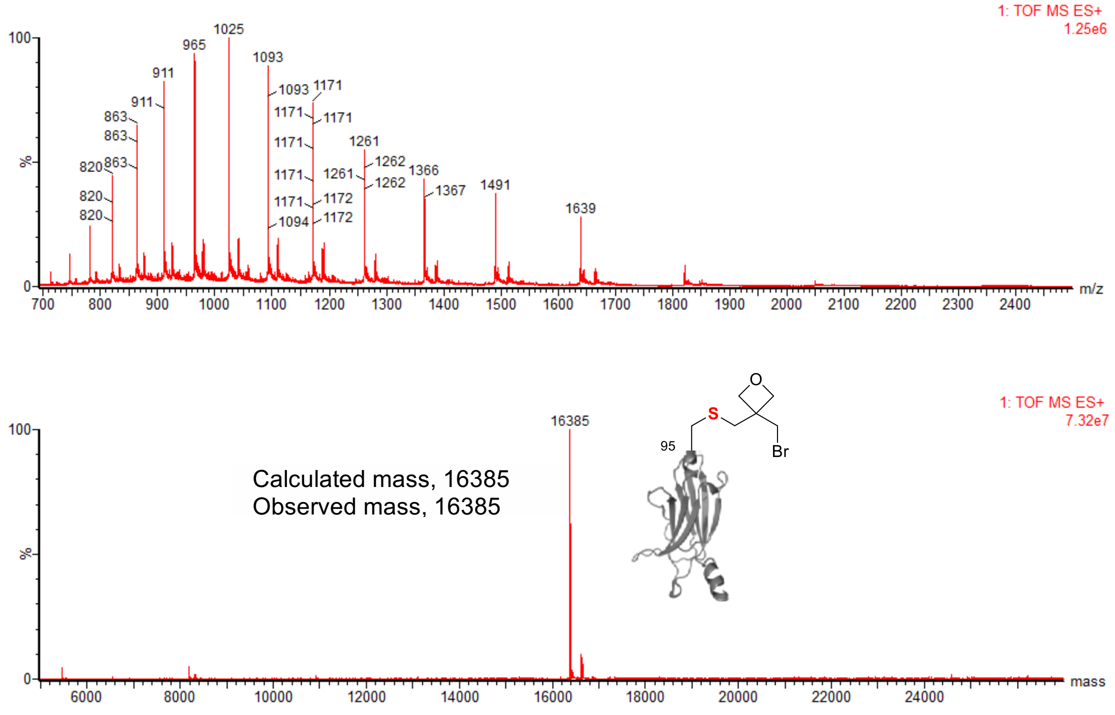

Figure S25. ESI–MS of C2Am-BrCH$_2$-*S*-Ox (**5**) after storage at –20 ºC for >9 months in PBS (pH 7.6)

Control: incubation of C2Am-BrCH$_2$-*S*-Ox (5) with Ellman's reagent

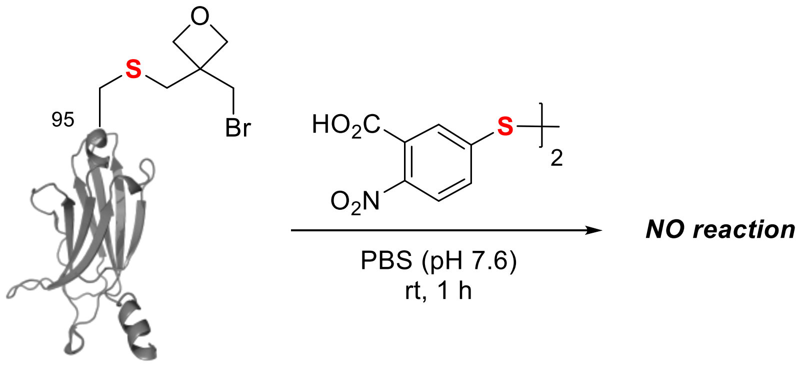

All manipulations were carried out at room temperature. A 10 µL aliquot of C2Am-BrCH$_2$-*S*-Ox (**5**) (*ca.* 1 mg/mL, 0.614 nmol) in PBS buffer at pH 7.6 was transferred to a 0.5 mL eppendorf tube. Ellman's reagent (0.3 µL of a 20 mg/mL stock solution in H_2O, 15.14 nmol) was added and the resulting mixture vortexed for 30 seconds. After 1 h of additional shaking, a 3 µL aliquot was analyzed by LC–MS (3 µL aliquot diluted by 7 µL of PBS buffer at pH 7.6) and starting protein (**2**) (calculated mass, 16278; observed mass, 16279) was detected unaltered.

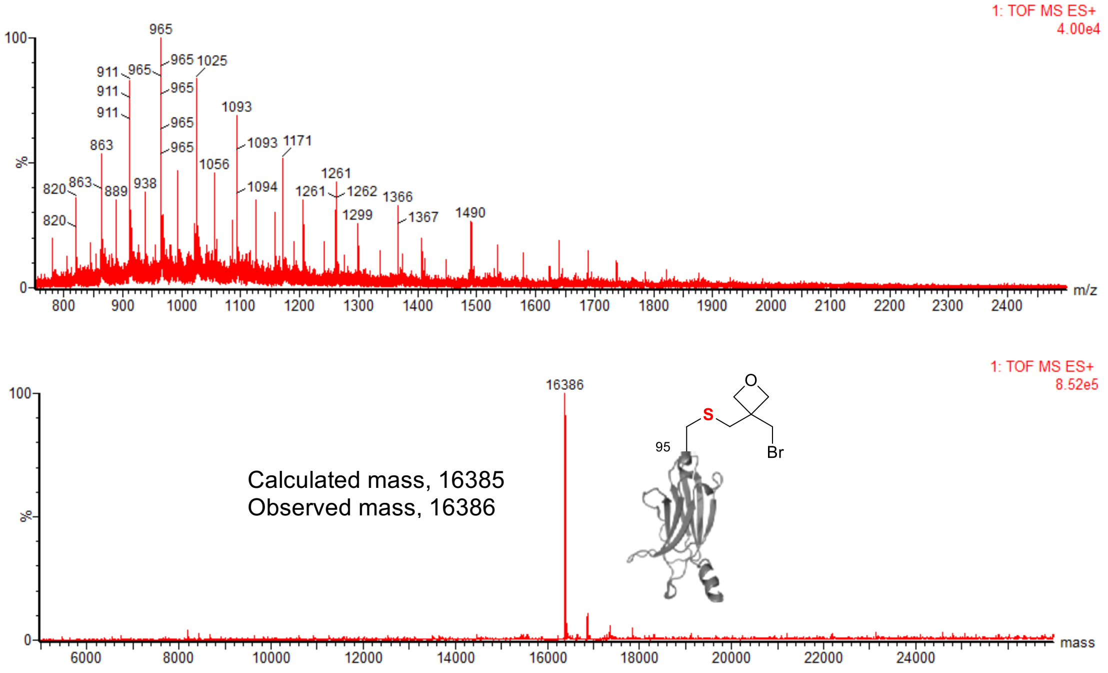

Figure S26. ESI–MS of the incubation of C2Am-BrCH$_2$-*S*-Ox (**5**) with Ellman's reagent

Reaction of C2Am-BrCH$_2$-*S*-Ox (5) with βGalSNa

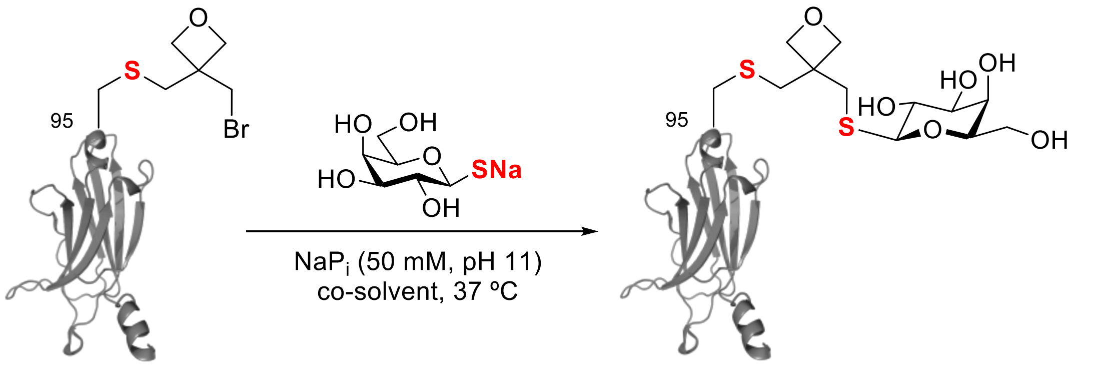

C2Am-BrCH$_2$-*S*-Ox (**5**) was prepared as a 0.34 mg/mL solution in 50 mM sodium phosphate buffer at pH 11 and 10 µL (0.208 nmol) were transferred to a 0.5 mL eppendorf tube. βGalSNa (0.45 mg, 2.08 µmol) was added at room temperature and the resulting mixture vortexed for 30 seconds. After 23 h of additional shaking at 37 ºC, a 2 µL aliquot was analyzed by LC–MS (2 µL aliquot diluted by 8 µL of 50 mM sodium phosphate buffer at pH 11) and complete conversion to C2Am-βGalSCH$_2$-*S*-Ox (**7a**) (calculated mass, 16500; observed mass, 16501) was observed.

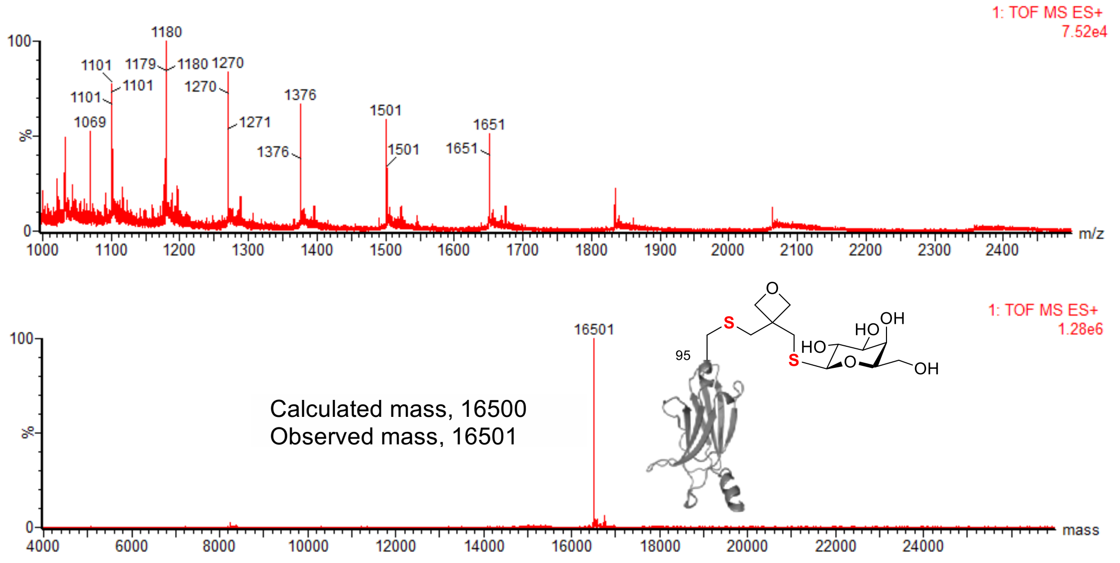

Figure S27. ESI–MS of the reaction of C2Am-BrCH$_2$-*S*-Ox (**5**) with βGalSNa

C2Am-BrCH$_2$-*S*-Ox (**5**) was prepared as a 0.34 mg/mL solution in 50 mM sodium phosphate buffer at pH 11 and 10 µL (0.208 nmol) were transferred to a 0.5 mL eppendorf tube. βGalSNa (1.2 µL of a 0.5 mg stock solution in 13 µL DMF, 208 nmol) was added at room temperature and the resulting mixture vortexed for 30 seconds. After 24 h of additional shaking at 37 ºC, a 2 µL aliquot was analyzed by

LC–MS (2 µL aliquot diluted by 8 µL of 50 mM sodium phosphate buffer at pH 11) and complete conversion to C2Am-βGalSCH$_2$-*S*-Ox (**7a**) (calculated mass, 16500; observed mass, 16501) was observed.

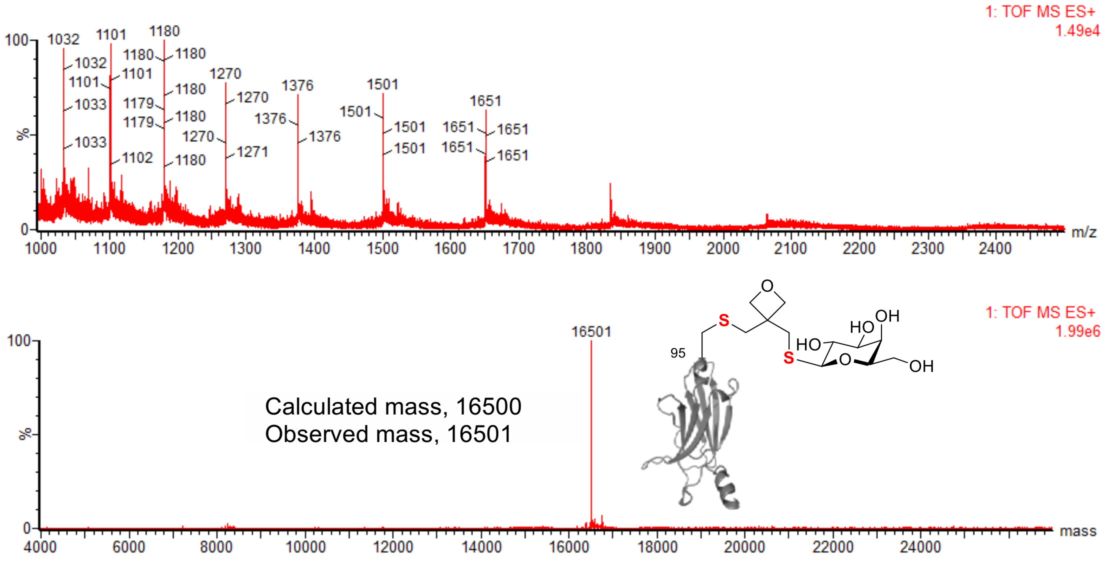

Figure S28. ESI–MS of the reaction of C2Am-BrCH$_2$-*S*-Ox (**5**) with βGalSNa and 10% DMF

Reaction of C2Am-BrCH$_2$-*S*-Ox (5) with BME

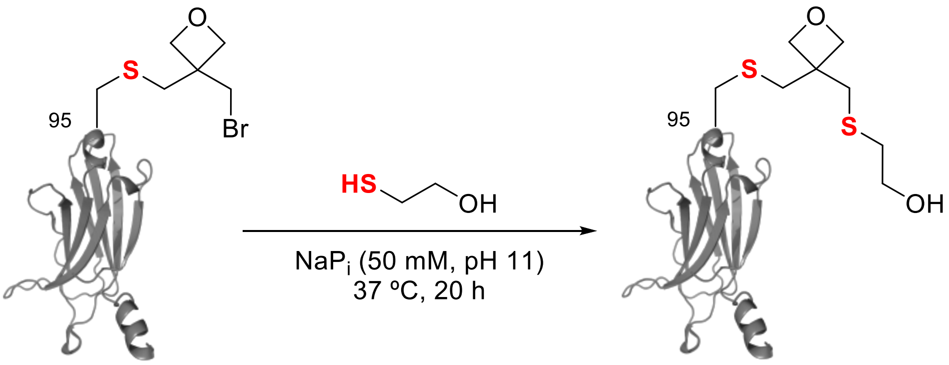

C2Am-BrCH$_2$-*S*-Ox (**5**) was prepared as a 0.34 mg/mL solution in 50 mM sodium phosphate buffer at pH 11 and 10 µL (0.208 nmol) were transferred to a 0.5 mL eppendorf tube. BME (0.15 µL mg, 2.08 µmol) was added at room temperature and the resulting mixture vortexed for 30 seconds. After 20 h of additional shaking at 37 ºC, a 2 µL aliquot was analyzed by LC–MS (2 µL aliquot diluted by 8 µL of 50 mM sodium phosphate buffer at pH 11) and complete conversion to C2Am-BMECH$_2$-*S*-Ox (**7b**) (calculated mass, 16382; observed mass, 16384) was observed.

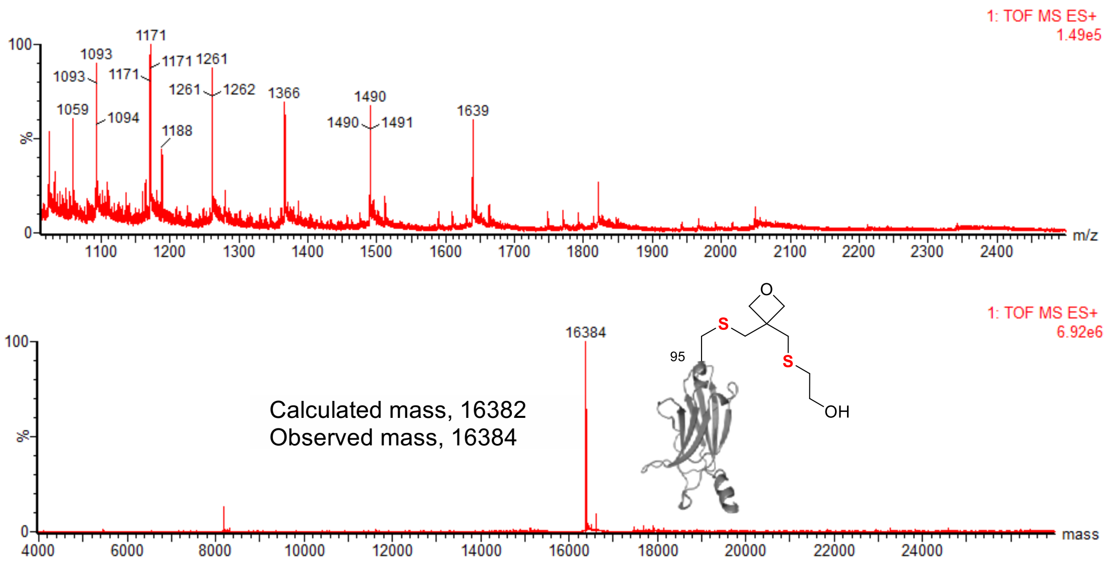

Figure S29. ESI–MS of the reaction of C2Am-BrCH_2-*S*-Ox (**5**) with BME

Control (starting material ***5*** *and product* ***7b*** *have similar masses):* to the above reaction mixture, PhSH (1.2 µL of a 0.5 mg stock solution in 13 µL DMF, 208 nmol) was added at room temperature and the resulting mixture vortexed for 30 seconds. After 15 h of additional shaking at 37 ºC, a 2 µL aliquot was analyzed by LC–MS (2 µL aliquot diluted by 8 µL of 50 mM sodium phosphate buffer at pH 11) and C2Am-BMECH_2-*S*-Ox (**7b**) (calculated mass, 16382; observed mass, 16383) was detected unaltered.

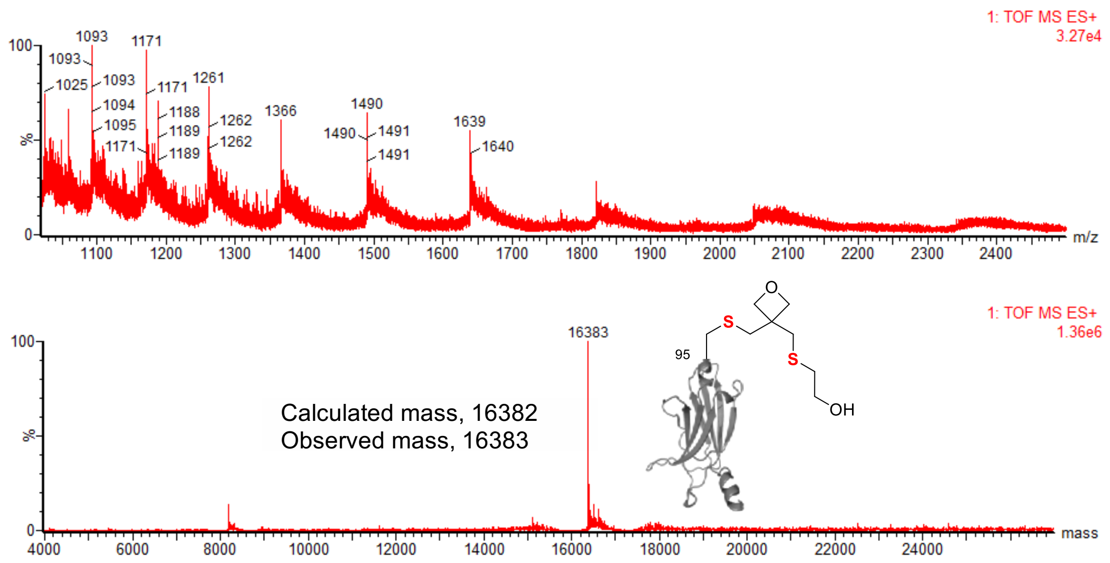

Figure S30. ESI–MS of the incubation of C2Am-BMECH_2-*S*-Ox (**7b**) with PhSH and 10% DMF

Reaction of C2Am-BrCH$_2$-*S*-Ox (5) with PhSH

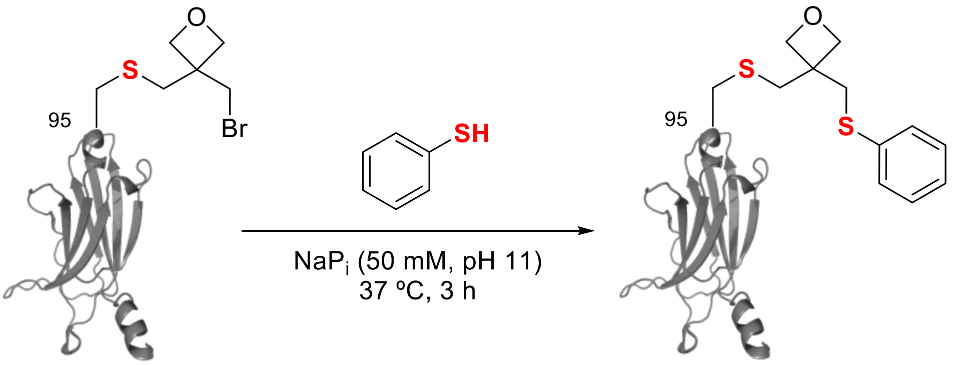

C2Am-BrCH$_2$-*S*-Ox (**5**) was prepared as a 0.34 mg/mL solution in 50 mM sodium phosphate buffer at pH 11 and 10 µL (0.208 nmol) were transferred to a 0.5 mL eppendorf tube. BME (0.25 µL mg, 2.08 µmol) was added at room temperature and the resulting mixture vortexed for 30 seconds. After 3 h of additional shaking at 37 ºC, a 2 µL aliquot was analyzed by LC–MS (2 µL aliquot diluted by 8 µL of 50 mM sodium phosphate buffer at pH 11) and complete conversion to C2Am-PhSCH$_2$-*S*-Ox (**7c**) (calculated mass, 16414; observed mass, 16415) was observed.

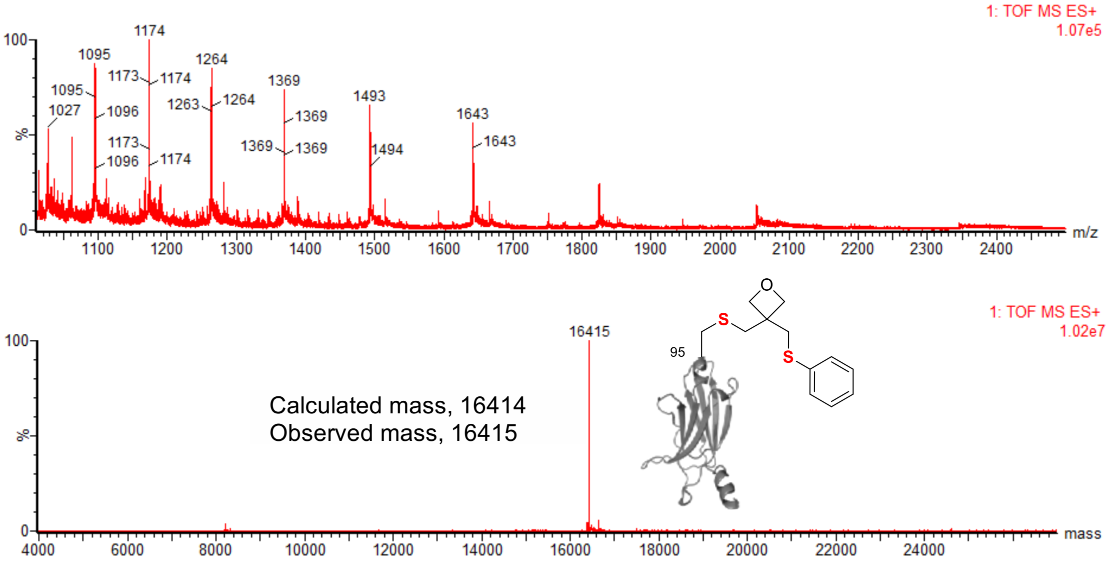

Figure S31. ESI–MS of the reaction of C2Am-BrCH$_2$-*S*-Ox (**5**) with PhSH

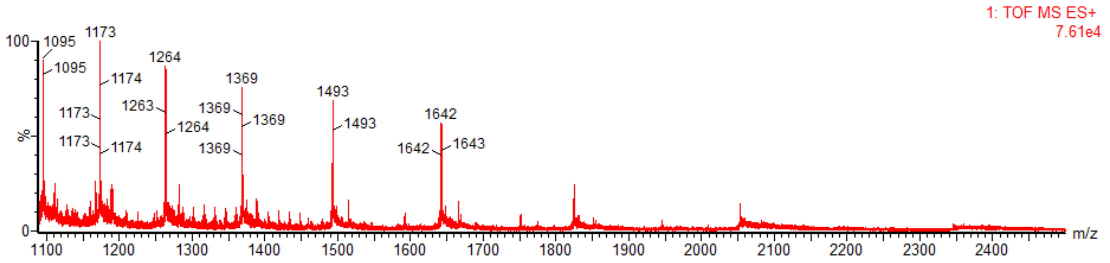

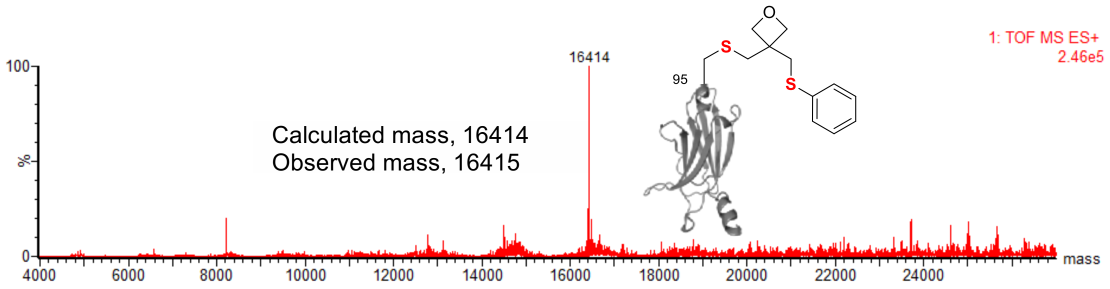

Figure S32. ESI–MS of the reaction of C2Am-BrCH$_2$-*S*-Ox (**5**) with PhSH; 15 h of extended reaction time once the alkylation is completed

Reaction of C2Am-BrCH$_2$-*S*-Ox (5) with BnNH$_2$

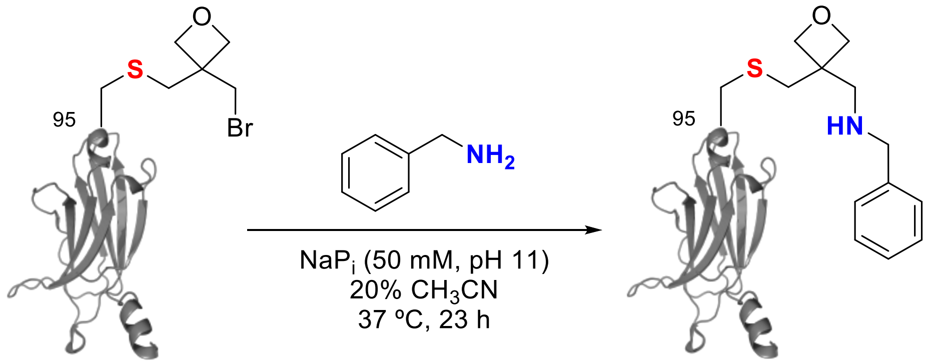

C2Am-BrCH$_2$-*S*-Ox (**5**) was prepared as a 0.34 mg/mL solution in 50 mM sodium phosphate buffer at pH 11 and 10 µL (0.208 nmol) were transferred to a 0.5 mL eppendorf tube. BnNH$_2$ (0.23 µL, 2.08 µmol) in CH_3CN (2.5 µL) was added at room temperature and the resulting mixture vortexed for 30 seconds. After 23 h of additional shaking at 37 ºC, a 2 µL aliquot was analyzed by LC–MS (2 µL aliquot diluted by 8 µL of 50 mM sodium phosphate buffer at pH 11) and complete conversion to C2Am-BnNHCH$_2$-*S*-Ox (**7d**) (calculated mass, 16411; observed mass, 16413) was observed.

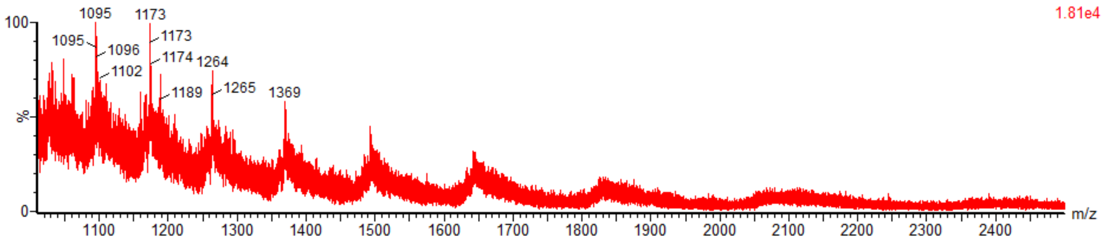

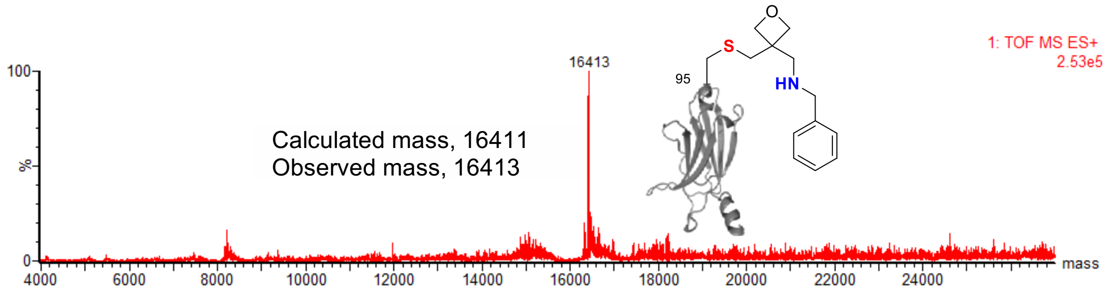

Figure S33. ESI–MS of the reaction of C2Am-BrCH$_2$-*S*-Ox (**5**) with BnNH$_2$

Stability of C2Am-PhSCH$_2$-*S*-Ox (7c) in human plasma

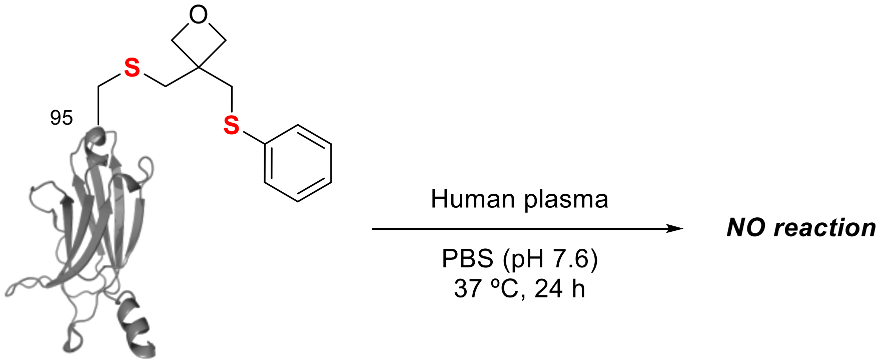

C2Am-PhSCH$_2$-*S*-Ox (**7c**) was prepared as a *ca.* 0.34 mg/mL solution in PBS buffer at pH 7.6 and 10 µL (0.208 nmol) were transferred to a 0.5 mL eppendorf tube. 1 µL of reconstituted human plasma (Sigma-Aldrich) was added at room temperature and the resulting mixture vortexed for 30 seconds. After 24 h of additional shaking at 37 ºC, a 2 µL aliquot was analyzed by LC–MS (2 µL aliquot diluted by 8 µL of PBS buffer at pH 7.6) and starting protein (**7c**) (calculated mass, 16414; observed mass, 16415) was detected unaltered.

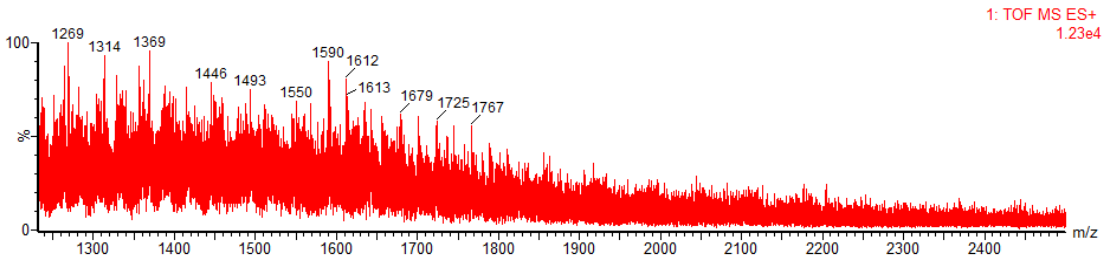

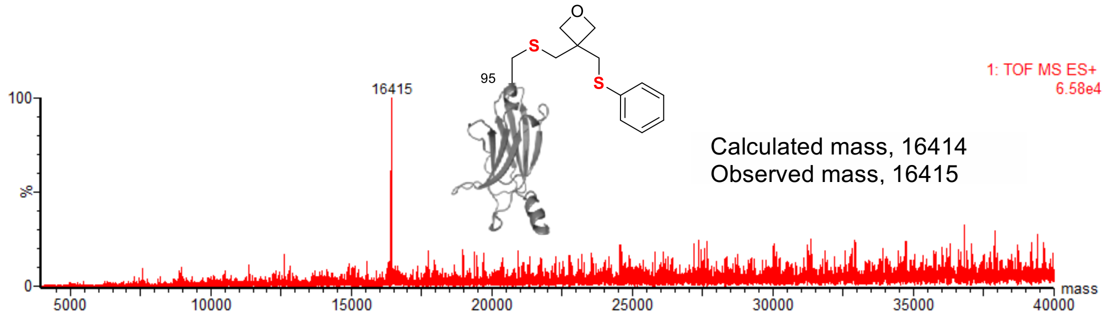

Figure S34. ESI–MS of the incubation of C2Am-PhSCH$_2$-*S*-Ox (**7c**) with human plasma

Stability of C2Am-PhSCH$_2$-*S*-Ox (7c) in the presence of GSH

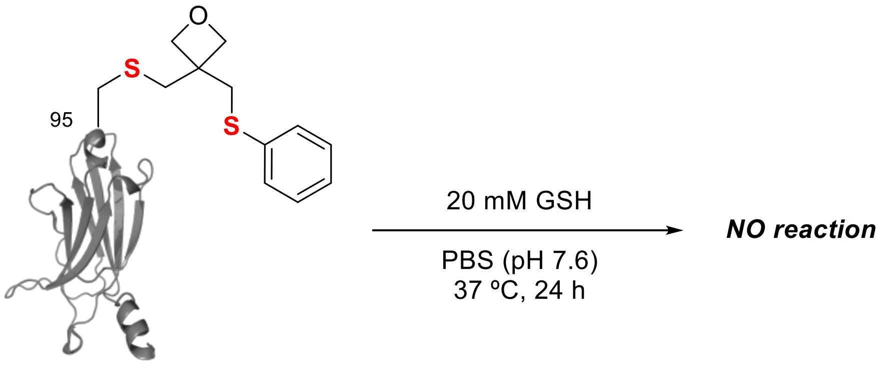

C2Am-PhSCH$_2$-*S*-Ox (**7c**) was prepared as a *ca.* 0.34 mg/mL solution in PBS buffer at pH 7.6 and 10 µL (0.208 nmol) were transferred to a 0.5 mL eppendorf tube. 1 µL of 20 mM GSH (6 mg in 1 mL of PBS buffer at pH 7.6) was added at room temperature and the resulting mixture vortexed for 30 seconds. After 24 h of additional shaking at 37 ºC, a 2 µL aliquot was analyzed by LC–MS (2 µL aliquot diluted by 8 µL of PBS buffer at pH 7.6) and starting protein (**7c**) (calculated mass, 16414; observed mass, 16415) was detected unaltered.

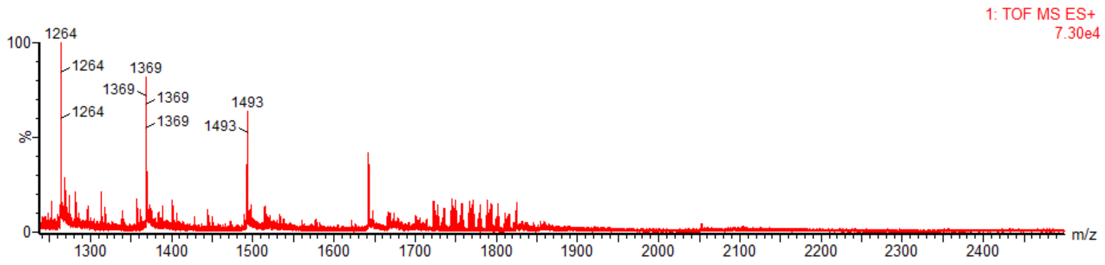

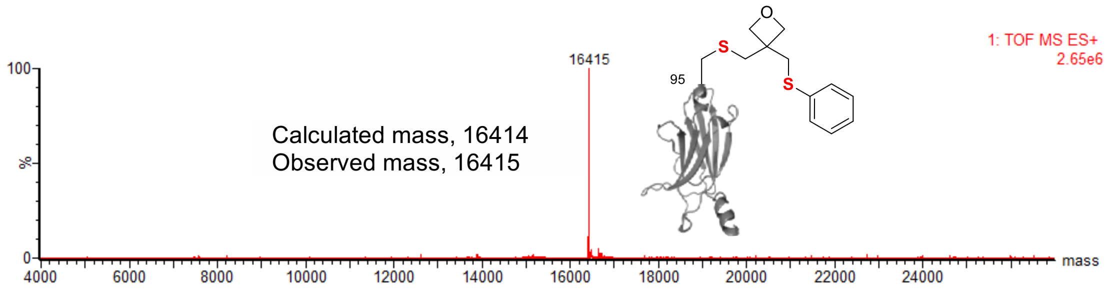

Figure S35. ESI–MS of the incubation of C2Am-PhSCH$_2$-*S*-Ox (**7c**) with 20 mM GSH

Reaction of C2Am with 3-(bromomethyl)oxetane (9)

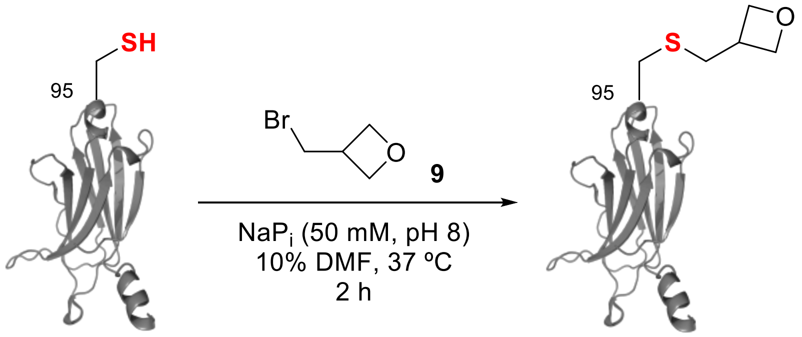

Tris(2-carboxyethyl)phosphine hydrochloride (TCEP·HCl) (5 µL of a 9 mg/mL stock solution in H_2O, 154.1 nmol) was added to a solution of C2Am (25 µL, 1 mg/mL, 1.54 nmol) in 50 mM sodium phosphate buffer at pH 8 and the resulting mixture shaken at room temperature for 2 h. C2Am was prepared as a *ca.* 0.25 mg/mL solution in 50 mM sodium phosphate buffer at pH 8 and 50 µL (0.77 nmol) were transferred to a 0.5 mL eppendorf tube. 3-(Bromomethyl)oxetane (**9**) (0.20 mg, 1.16 µmol) in DMF (6 µL) was added and the resulting mixture vortexed for 30 seconds. After 2 h of additional shaking at 37 ºC, a 3 µL aliquot was analyzed by LC–MS (3 µL aliquot diluted by 7 µL of 50 mM sodium phosphate buffer at pH 8) and complete conversion to C2Am-SCH$_2$-Ox (**10**) (calculated mass, 16292; observed mass, 16295) was observed. Small molecules were removed from the reaction mixture by loading the sample onto a Zeba™ Spin desalting column (ThermoFisher Scientific) previously equilibrated with PBS buffer at pH 7.6. The sample was eluted by centrifugation (2 min, 1500xg). The protein sample (0.25 mg/mL by Bradford assay) was flash frozen with liquid nitrogen and stored at –20 ºC.

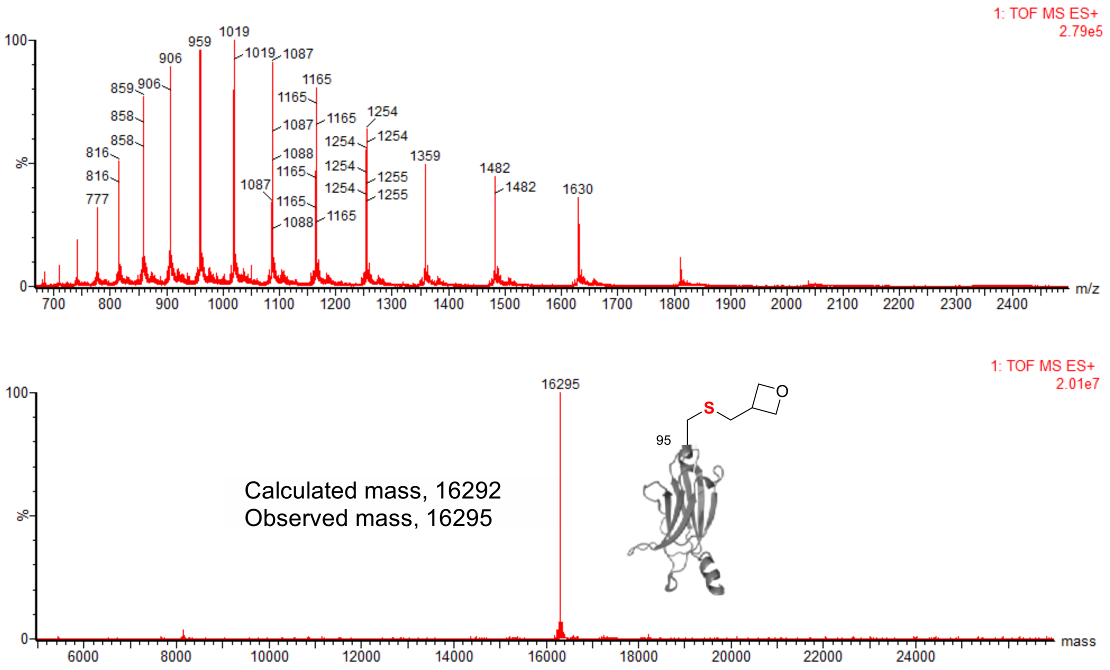

Figure S36. ESI–MS of the reaction of C2Am with 3-(bromomethyl)oxetane (**9**)

Control: incubation of C2Am-SCH$_2$-Ox (10) with Ellman's reagent

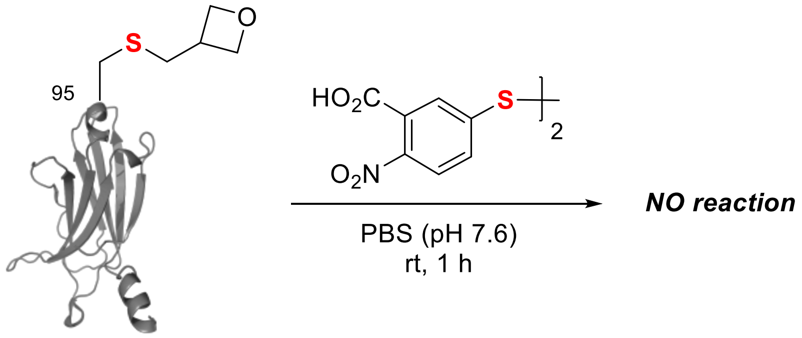

All manipulations were carried out at room temperature. A 10 µL aliquot of C2Am-SCH$_2$-Ox (**10**) (*ca.* 0.25 mg/mL, 0.153 nmol) in PBS buffer at pH 7.6 was transferred to a 0.5 mL eppendorf tube. Ellman's reagent (0.2 µL of a 20 mg/mL stock solution in H_2O, 10.09 nmol) was added and the resulting mixture vortexed for 30 seconds. After 1 h of additional shaking, a 3 µL aliquot was analyzed by LC–MS (3 µL aliquot diluted by 7 µL of PBS buffer at pH 7.6) and starting protein (**10**) (calculated mass, 16292; observed mass, 16295) was detected unaltered.

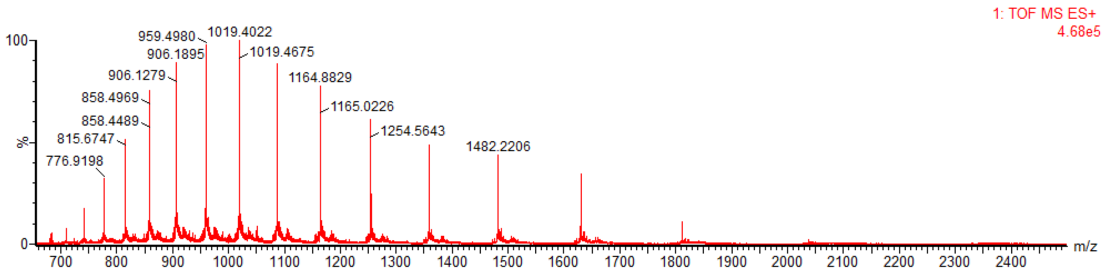

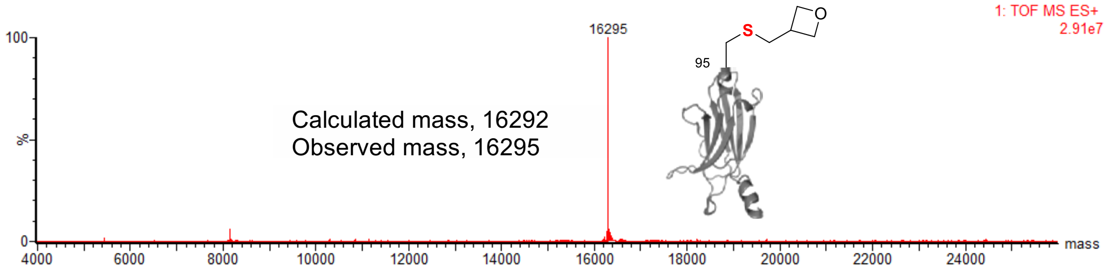

Figure S37. ESI–MS of the incubation of C2Am-SCH$_2$-Ox (**10**) with Ellman's reagent

Reaction of C2Am-SCH$_2$-Ox (10) with BnNH$_2$

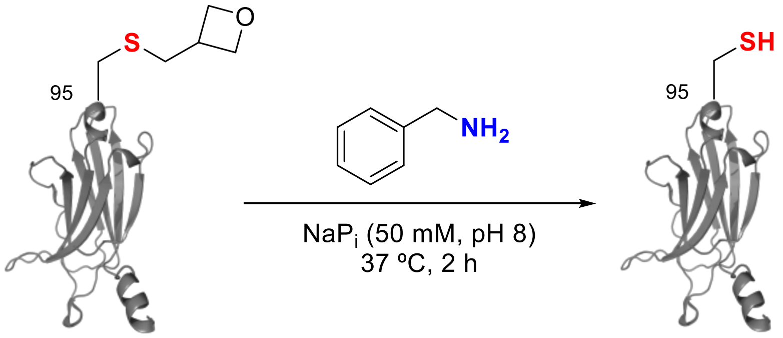

C2Am-SCH$_2$-Ox (**10**) was prepared as a 0.25 mg/mL solution in 50 mM sodium phosphate buffer at pH 8 and 10 µL (0.153 nmol) were transferred to a 0.5 mL eppendorf tube. BnNH$_2$ (2 µL of an 8.2 mg/mL stock solution in H_2O, 15.34 µmol) was added at room temperature and the resulting mixture vortexed for 30 seconds. After 2 h of additional shaking at 37 ºC, a 2 µL aliquot was analyzed by LC–MS (2 µL aliquot diluted by 8 µL of 50 mM sodium phosphate buffer at pH 8) and complete conversion to C2Am (calculated mass, 16222; observed mass, 16222) was observed.

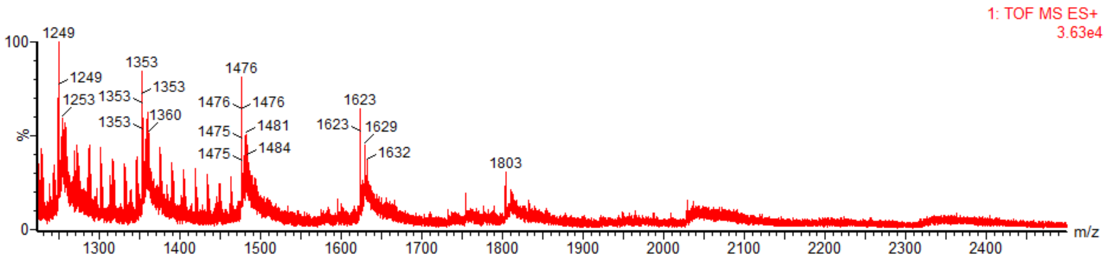

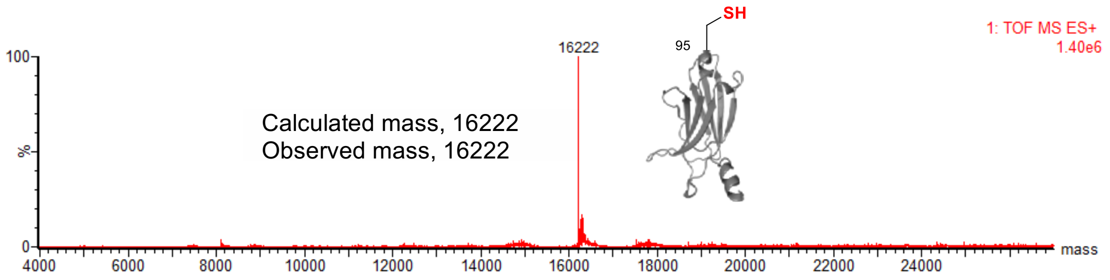

Figure S38. ESI–MS of the reaction of C2Am-SCH$_2$-Ox (**10**) with BnNH$_2$

Control (incubation with Ellman's reagent): to the above reaction mixture, Ellman's reagent (3 µL of a 20 mg/mL stock solution in H_2O, 151.35 nmol) was added at room temperature and the resulting mixture vortexed for 30 seconds. After 1 h of additional shaking at 37 ºC, a 2 µL aliquot was analyzed by LC–MS (2 µL aliquot diluted by 8 µL of 50 mM sodium phosphate buffer at pH 8) and complete conversion to the expected Ellman's product (calculated mass, 16419; observed mass, 16420) was observed.

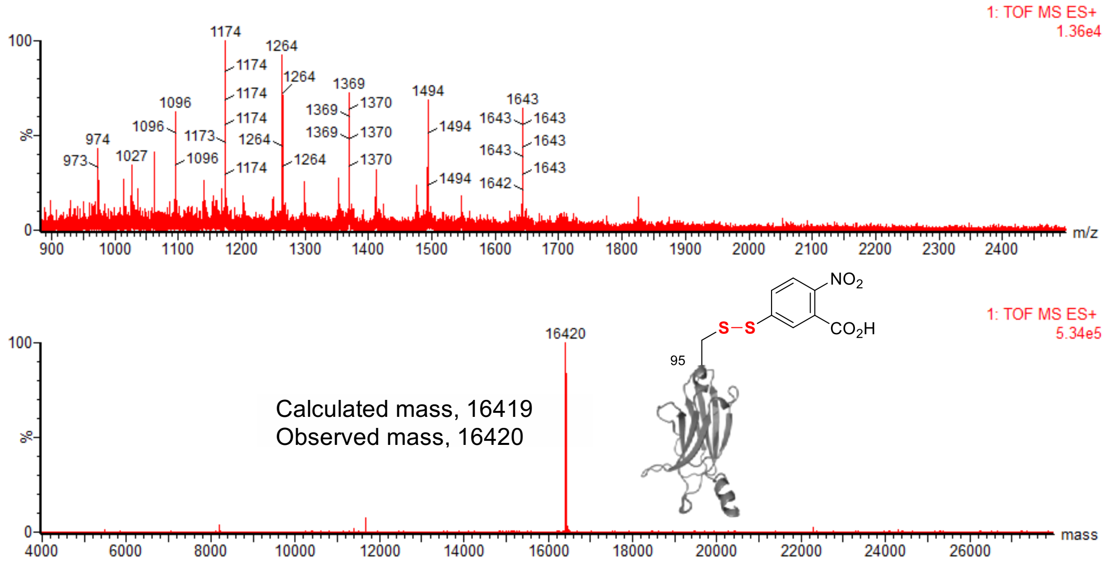

Figure S39. ESI–MS of the reaction of C2Am with Ellman's reagent

Reaction of C2Am-SCH$_2$-Ox (10) with BME

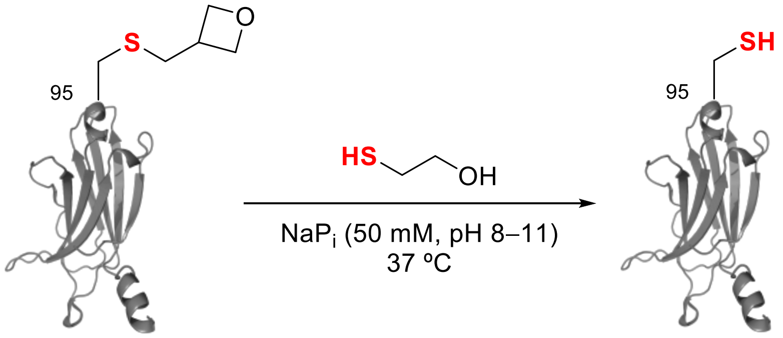

C2Am-SCH$_2$-Ox (**10**) was prepared as a 0.25 mg/mL solution in 50 mM sodium phosphate buffer at pH 8 and 10 µL (0.153 nmol) were transferred to a 0.5 mL eppendorf tube. BME (0.11 µL, 1.53 µmol) was added at room temperature and the resulting mixture vortexed for 30 seconds. After 22 h of additional shaking at 37 ºC, a 2 µL aliquot was analyzed by LC–MS (2 µL aliquot diluted by 8 µL of 50 mM sodium phosphate buffer at pH 8) and 50% conversion to C2Am (calculated mass, 16222; observed mass, 16222) together with 50% conversion to the corresponding mixed BME disulfide (calculated mass, 16298; observed mass, 16298) was observed.

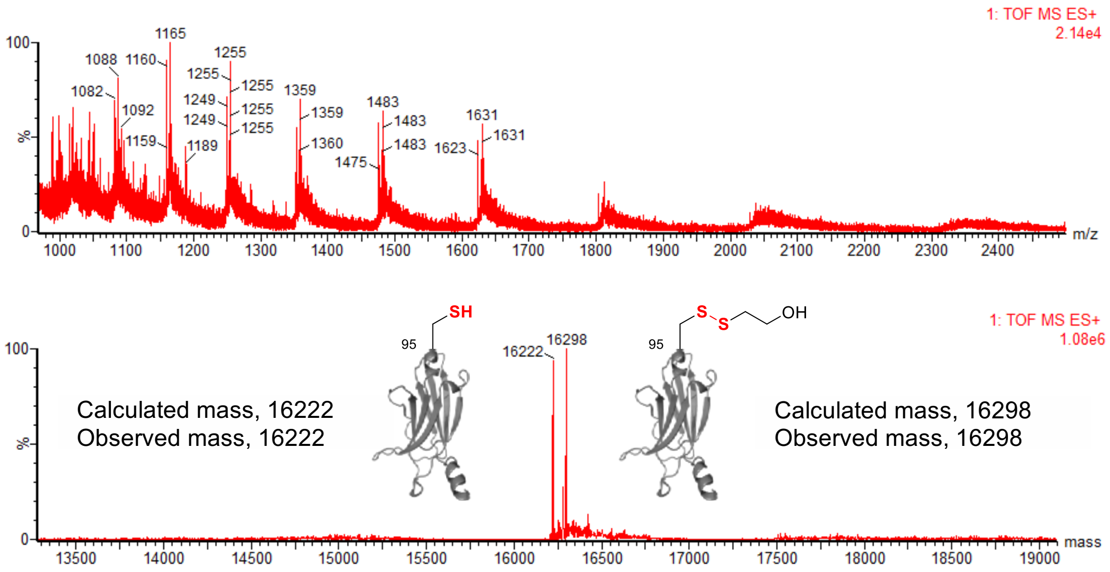

Figure S40. ESI–MS of the reaction of C2Am-SCH$_2$-Ox (**10**) with BME (pH 8)

C2Am-SCH$_2$-Ox (**10**) was prepared as a 0.25 mg/mL solution in 50 mM sodium phosphate buffer at pH 11 and 10 µL (0.153 nmol) were transferred to a 0.5 mL eppendorf tube. BME (0.11 µL, 1.53 µmol) was added at room temperature and the resulting mixture vortexed for 30 seconds. After 4 h of additional shaking at 37 ºC, a 2 µL aliquot was analyzed by LC–MS (2 µL aliquot diluted by 8 µL of 50 mM

sodium phosphate buffer at pH 11) complete conversion to the corresponding mixed BME disulfide (calculated mass, 16298; observed mass, 16298) was observed.

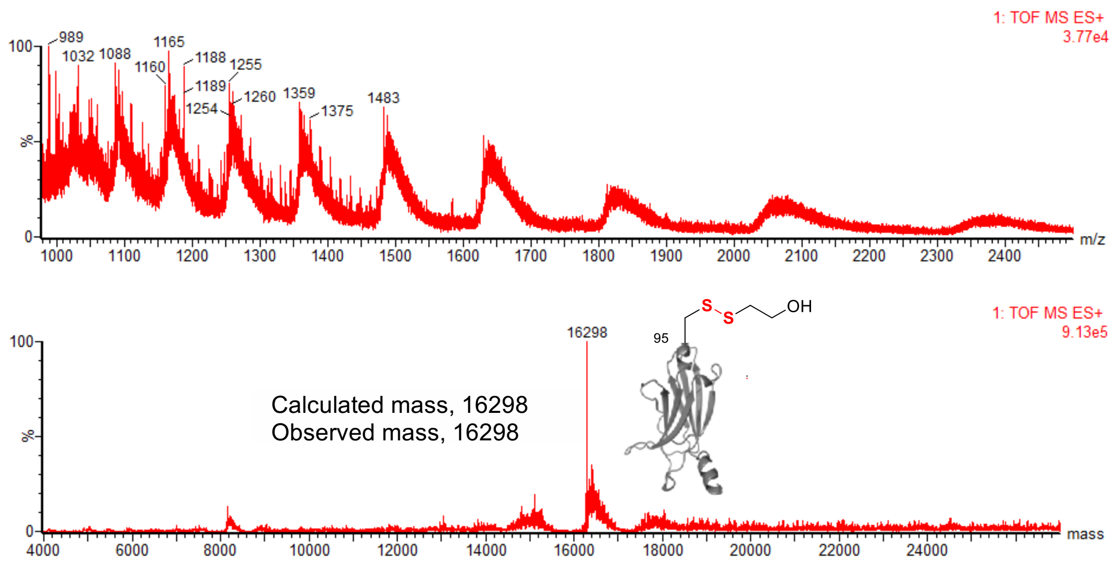

Figure S41. ESI–MS of the reaction of C2Am-SCH$_2$-Ox (**10**) with BME (pH 11)

Reaction of C2Am-SCH$_2$-Ox (10) with PhSH

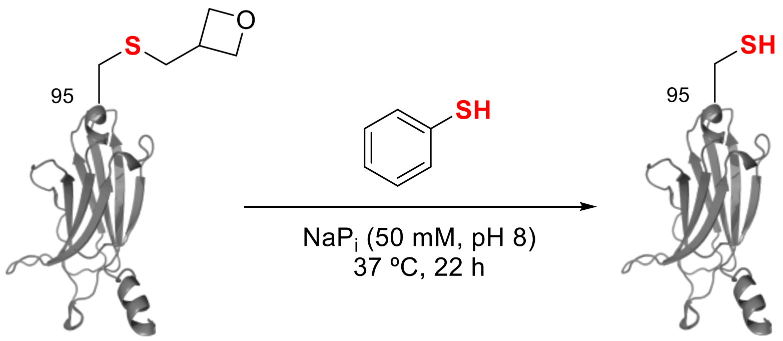

C2Am-SCH$_2$-Ox (**10**) was prepared as a 0.25 mg/mL solution in 50 mM sodium phosphate buffer at pH 8 and 10 µL (0.153 nmol) were transferred to a 0.5 mL eppendorf tube. PhSH (0.16 µL, 1.53 µmol) was added at room temperature and the resulting mixture vortexed for 30 seconds. After 22 h of additional shaking at 37 ºC, a 2 µL aliquot was analyzed by LC–MS (2 µL aliquot diluted by 8 µL of 50 mM sodium phosphate buffer at pH 8) and complete conversion to C2Am (calculated mass, 16222; observed mass, 16222) was observed.

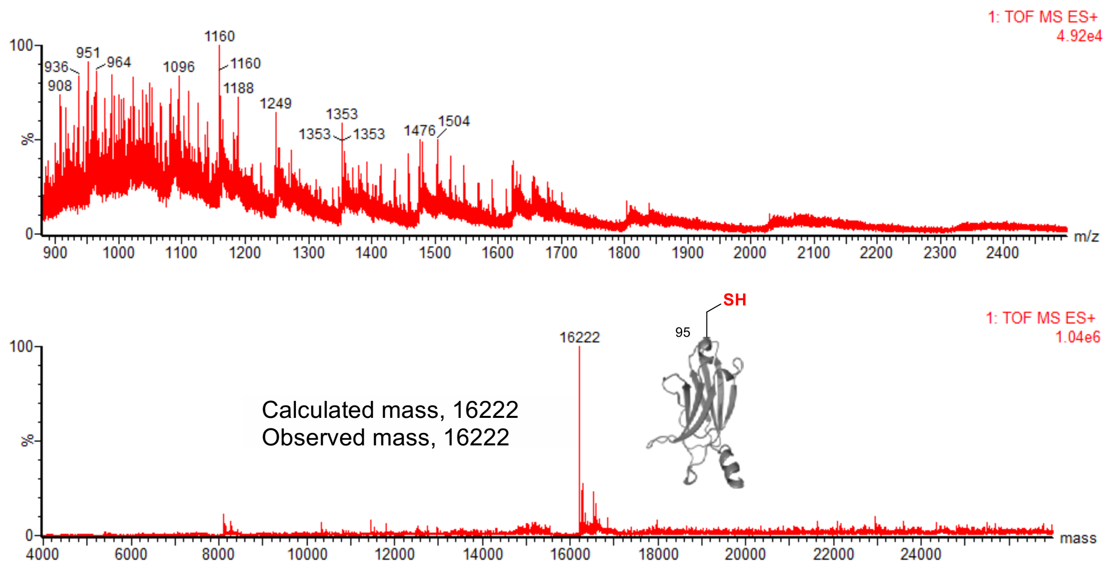

Figure S42. ESI–MS of the reaction of C2Am-SCH_2-Ox (**10**) with PhSH

Control (incubation with Ellman's reagent): to the above reaction mixture, Ellman's reagent (3 µL of a 20 mg/mL stock solution in H_2O, 151.35 nmol) was added at room temperature and the resulting mixture vortexed for 30 seconds. After 1 h of additional shaking at 37 ºC, a 2 µL aliquot was analyzed by LC–MS (2 µL aliquot diluted by 8 µL of 50 mM sodium phosphate buffer at pH 8) and complete conversion to the expected Ellman's product (calculated mass, 16419; observed mass, 16420) was observed.

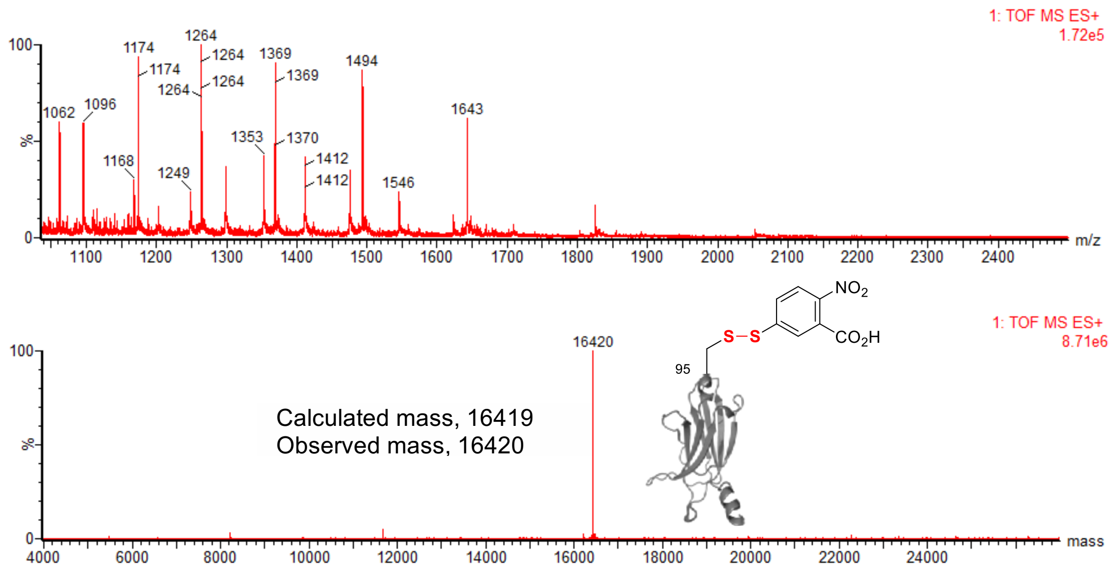

Figure S43. ESI–MS of the reaction of C2Am with Ellman's reagent

Reaction of C2Am-SCH$_2$-Ox (10) with βGalSNa

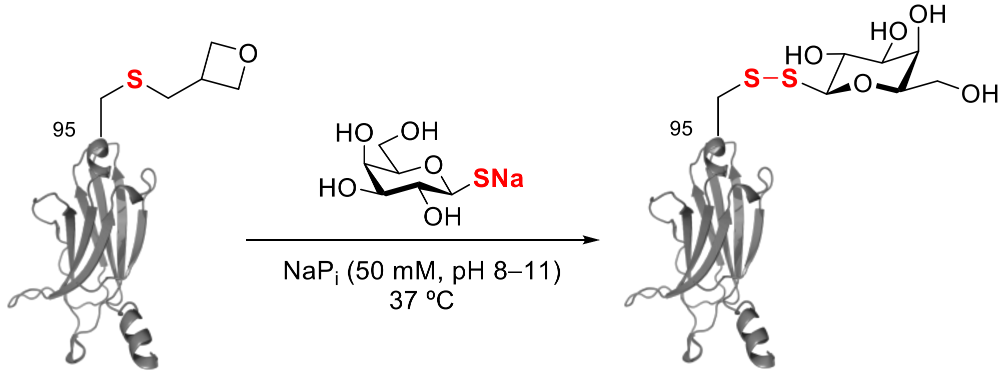

C2Am-SCH$_2$-Ox (**10**) was prepared as a 0.25 mg/mL solution in 50 mM sodium phosphate buffer at pH 8 and 10 μL (0.153 nmol) were transferred to a 0.5 mL eppendorf tube. βGalSNa (0.33 mg, 1.53 μmol) was added at room temperature and the resulting mixture vortexed for 30 seconds. After 5 h of additional shaking at 37 ºC, a 2 μL aliquot was analyzed by LC–MS (2 μL aliquot diluted by 8 μL of 50 mM sodium phosphate buffer at pH 8) and complete conversion to the corresponding mixed βGal disulfide (calculated mass, 16416; observed mass, 16417) was observed.

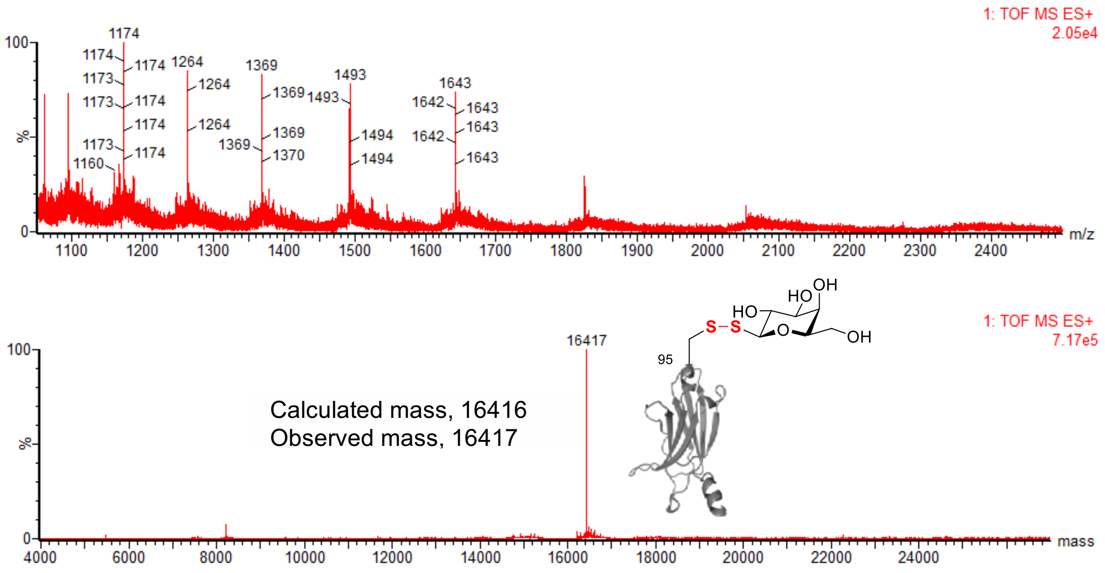

Figure S44. ESI–MS of the reaction of C2Am-SCH$_2$-Ox (**10**) with βGalSNa (pH 8)

C2Am-SCH$_2$-Ox (**10**) was prepared as a 0.25 mg/mL solution in 50 mM sodium phosphate buffer at pH 11 and 10 μL (0.153 nmol) were transferred to a 0.5 mL eppendorf tube. βGalSNa (0.33 mg, 1.53 μmol) was added at room temperature and the resulting mixture vortexed for 30 seconds. After 2 h of additional shaking at 37 ºC, a 2 μL aliquot was analyzed by LC–MS (2 μL aliquot diluted by 8 μL of 50 mM

sodium phosphate buffer at pH 11) and complete conversion to the corresponding mixed βGal disulfide (calculated mass, 16416; observed mass, 16417) was observed.

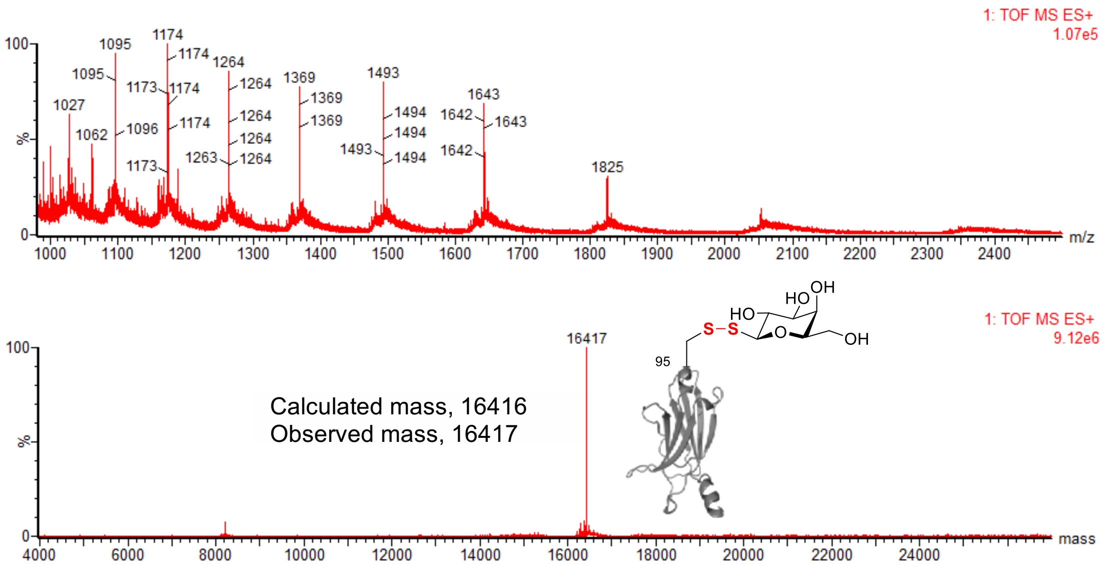

Figure S45. ESI–MS of the reaction of C2Am-SCH$_2$-Ox (**10**) with βGalSNa (pH 11)

Control (incubation with TCEP·HCl): to the above reaction mixture, TCEP·HCl (2 μL of an 11 mg/mL stock solution in H_2O, 678.0 nmol) was added at room temperature and the resulting mixture vortexed for 30 seconds. After 1 h of additional shaking at 37 ºC, a 2 μL aliquot was analyzed by LC–MS (2 μL aliquot diluted by 8 μL of 50 mM sodium phosphate buffer at pH 8) and complete conversion to C2Am (calculated mass, 16222; observed mass, 16223) was observed.

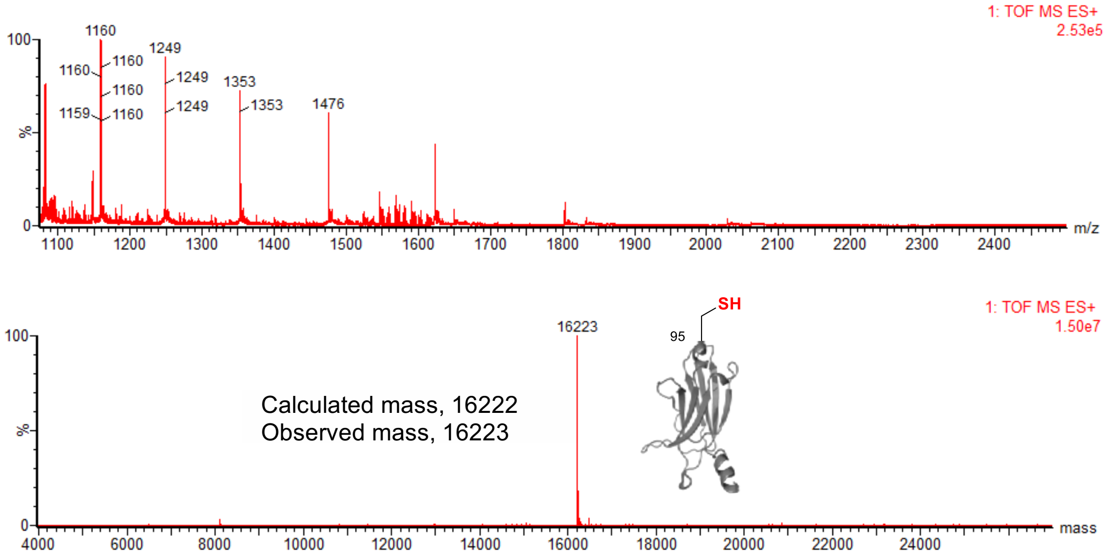

Figure S46. ESI–MS of the reaction of mixed βGal disulfide with TCEP·HCl

Reaction of C2Am-SCH$_2$-Ox (10) with TCEP·HCl

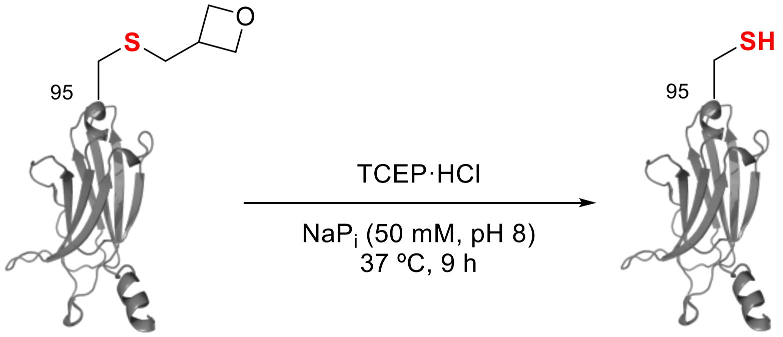

C2Am-SCH$_2$-Ox (**10**) was prepared as a 0.25 mg/mL solution in 50 mM sodium phosphate buffer at pH 8 and 10 μL (0.153 nmol) were transferred to a 0.5 mL eppendorf tube. TCEP·HCl (2 μL of an 11 mg/mL stock solution in H_2O, 678.0 nmol) was added at room temperature and the resulting mixture vortexed for 30 seconds. After 9 h of additional shaking at 37 ºC, a 2 μL aliquot was analyzed by LC–MS (2 μL aliquot diluted by 8 μL of 50 mM sodium phosphate buffer at pH 8) and complete conversion to C2Am (calculated mass, 16222; observed mass, 16223) was observed.

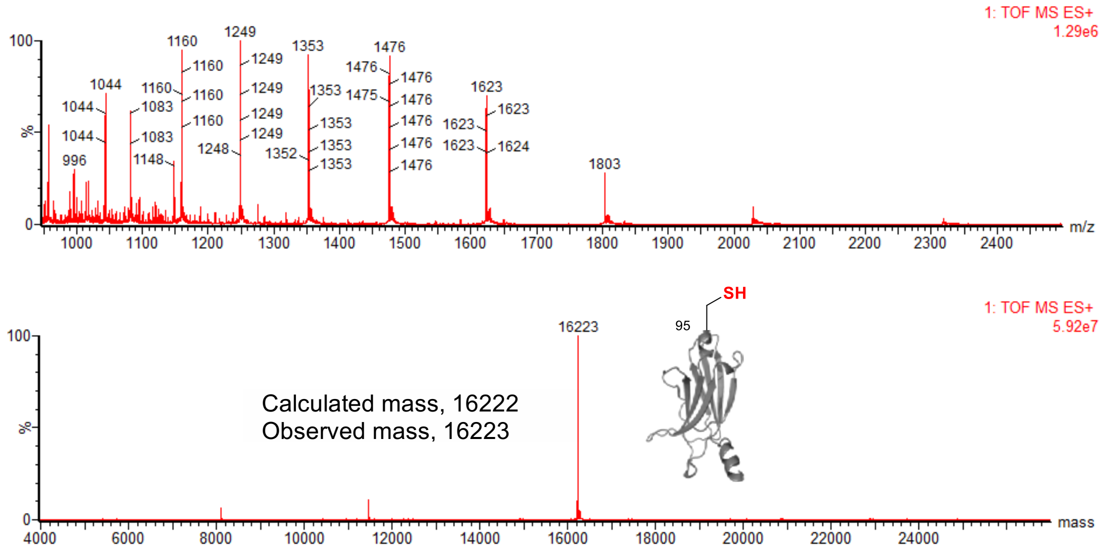

Figure S47. ESI–MS of the reaction of C2Am-SCH$_2$-Ox (**10**) with TCEP·HCl

Control: reaction of C2Am-Sac (S2) with BnNH$_2$

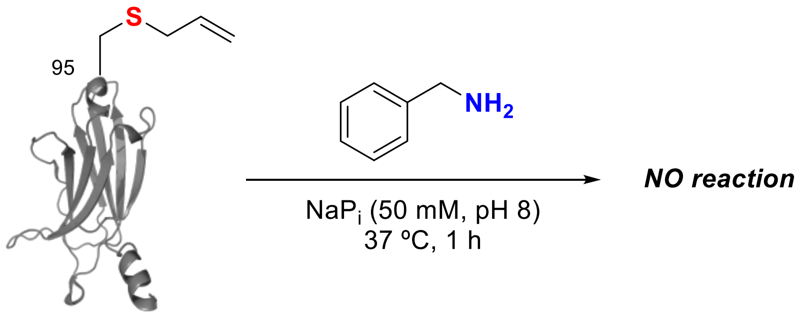

C2Am-Sac (**S2**)[11] was prepared as a 0.25 mg/mL solution in 50 mM sodium phosphate buffer at pH 8 and 10 µL (0.198 nmol) were transferred to a 0.5 mL eppendorf tube. BnNH$_2$ (2 µL of a 10.6 mg/mL stock solution in H_2O, 198.1 µmol) was added at room temperature and the resulting mixture vortexed for 30 seconds. After 1 h of additional shaking at 37 ºC, a 2 µL aliquot was analyzed by LC–MS (2 µL aliquot diluted by 8 µL of 50 mM sodium phosphate buffer at pH 8) and starting protein (**S2**) (calculated mass, 16262; observed mass, 16263) was detected unaltered.

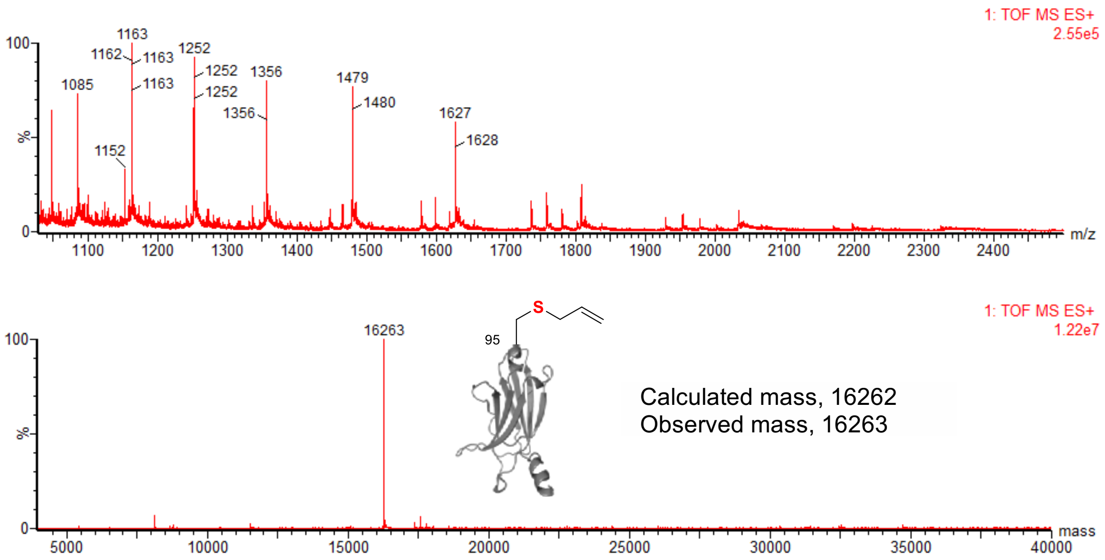

Figure S48. ESI–MS of the reaction of C2Am-Sac (**S2**) with BnNH$_2$

Control: reaction of C2Am-Sac (S2) with βGalSNa

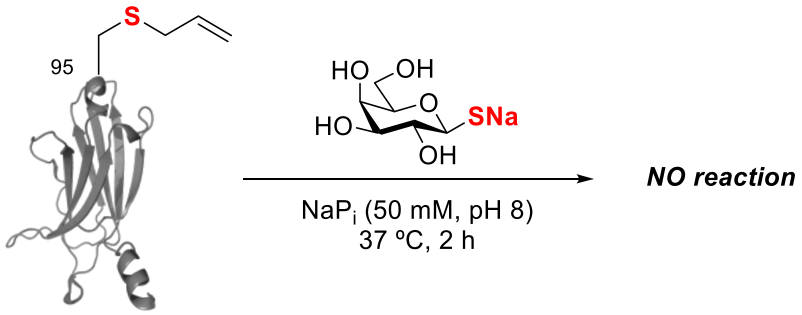

C2Am-Sac (**S2**)[11] was prepared as a 0.25 mg/mL solution in 50 mM sodium phosphate buffer at pH 8 and 10 μL (0.198 nmol) were transferred to a 0.5 mL eppendorf tube. βGalSNa (2 μL of a 0.6 mg stock solution in 277.8 μL H_2O, 198.1 μmol) was added at room temperature and the resulting mixture vortexed for 30 seconds. After 2 h of additional shaking at 37 ºC, a 2 μL aliquot was analyzed by LC–MS (2 μL aliquot diluted by 8 μL of 50 mM sodium phosphate buffer at pH 8) and starting protein (**S2**) (calculated mass, 16262; observed mass, 16262) was detected unaltered.

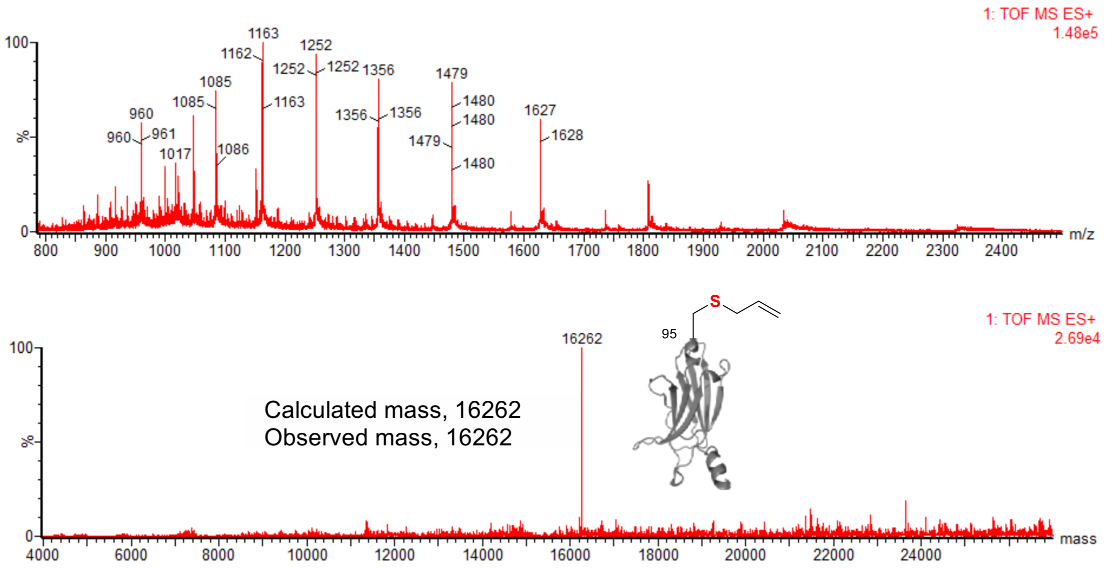

Figure S49. ESI–MS of the reaction of C2Am-Sac (**S2**) with βGalSNa

Annexin-V (AnxV)[12,13]

Sequence of AnxV C315 (modified residue highlighted)

AQVLRGTVTDFPGFDERADAETLRKAMKGLGTDEESILTLLTSRSNAQRQEISAAFKT
LFGRDLLDDLKSELTGKFEKLIVALMKPSRLYDAYELKHALKGAGTNEKVLTEIIASR
TPEELRAIKQVYEEEYGSSLEDDVVGDTSGYYQRMLVVLLQANRDPDAGIDEAQVE
QDAQALFQAGELKWGTDEEKFITIFGTRSVSHLRKVFDKYMTISGFQIEETIDRETSGN
LEQLLLAVVKSIRSIPAYLAETLYYAMKGAGTDDHTLIRVMVSRSEIDLFNIRKEFRK
NFATSLYSMIKGDTSGDYKKALLLLCGEDD

Calculated average isotopic mass = 35805.58 Da (*N*-terminal Met cleaved)

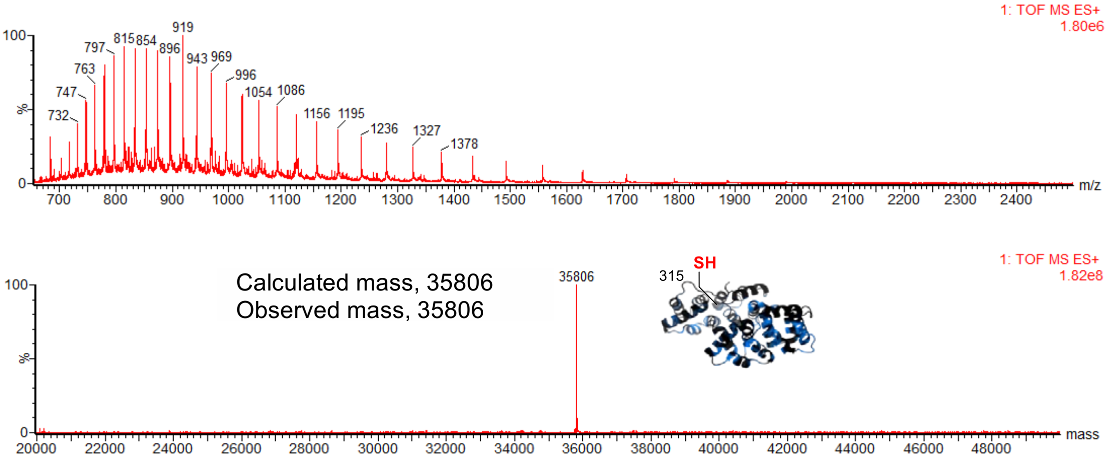

Figure S50. ESI–MS spectrum of AnxV

Control: reaction of AnxV with Ellman's reagent

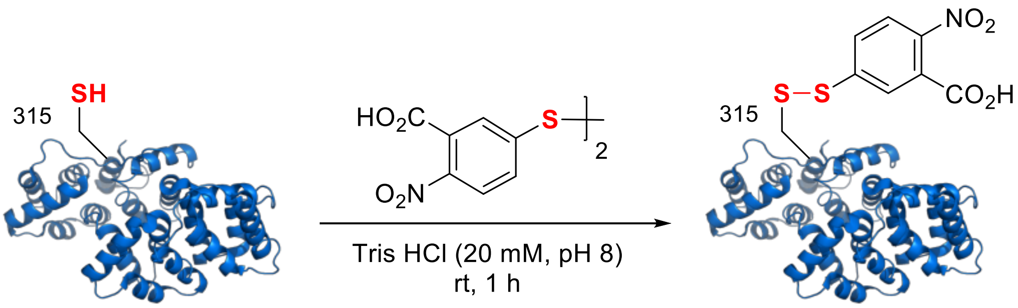

All manipulations were carried out at room temperature. A 10 µL aliquot of AnxV (1 mg/mL, 0.616 nmol) in 20 mM Tris HCl buffer at pH 8 was transferred to a 0.5 mL eppendorf tube. Ellman's reagent (2 µL of a 14 mg/mL stock solution in H_2O, 70.65 nmol) was added and the resulting mixture vortexed for 30 seconds. After 1 h of additional shaking, a 3 µL aliquot was analyzed by LC–MS (3 µL aliquot diluted by 7

µL of 20 mM Tris HCl buffer at pH 8) and complete conversion to the expected Ellman's product (calculated mass, 36003; observed mass, 36003) was observed.

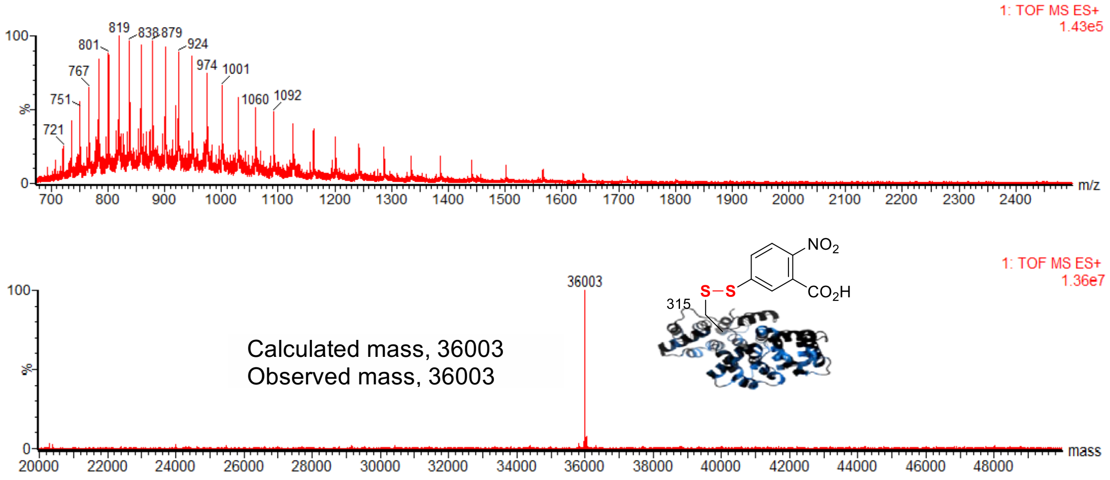

Figure S51. ESI–MS of the reaction of AnxV with Ellman's reagent

Reaction of AnxV with 3-bromooxetane (1)

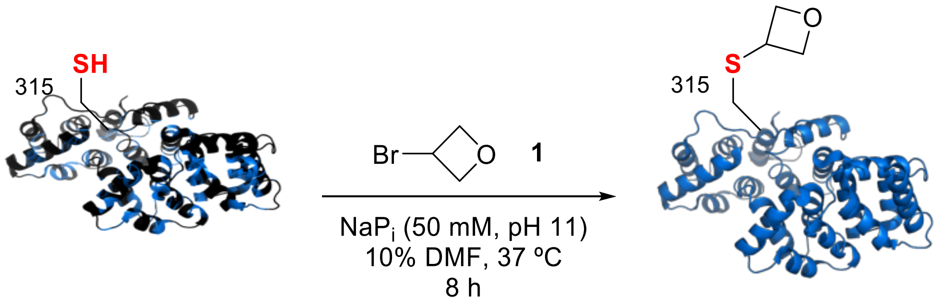

AnxV was prepared as a 0.25 mg/mL solution in 50 mM sodium phosphate buffer at pH 11 and 15 µL (0.105 nmol) were transferred to a 0.5 mL eppendorf tube. 3-Bromooxetane (**1**) (1.5 µL of a 0.8 mg stock solution in 158 µL DMF, 52.4 nmol) was added and the resulting mixture vortexed for 30 seconds. After 8 h of additional shaking, a 3 µL aliquot was analyzed by LC–MS (3 µL aliquot diluted by 7 µL of 50 mM sodium phosphate buffer at pH 11) and complete conversion to AnxV-3-*S*-Ox (**3**) (calculated mass, 35862; observed mass, 35859) was observed. Small molecules were removed from the reaction mixture by loading the sample onto a Zeba™ Spin desalting column (ThermoFisher Scientific) previously equilibrated with PBS buffer at pH 7.6. The sample was eluted by centrifugation (2 min, 1500xg). The protein sample (0.16 mg/mL by Bradford assay) was flash frozen with liquid nitrogen and stored at –20 ºC.

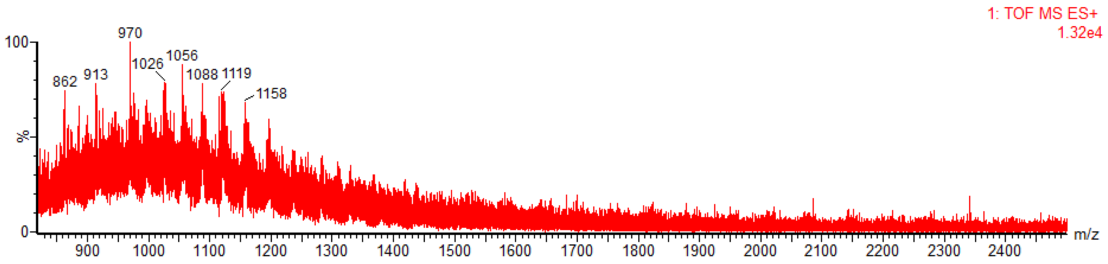

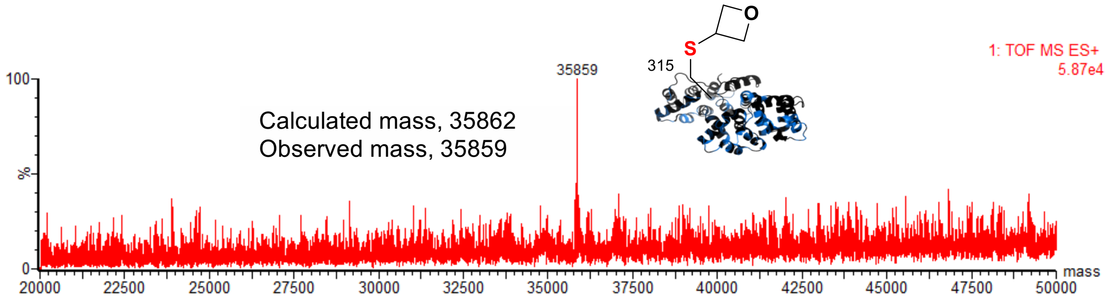

Figure S52. ESI–MS of the reaction of AnxV with 3-bromooxetane (**1**)

Control: incubation of AnxV-3-*S*-Ox (3) with Ellman's reagent

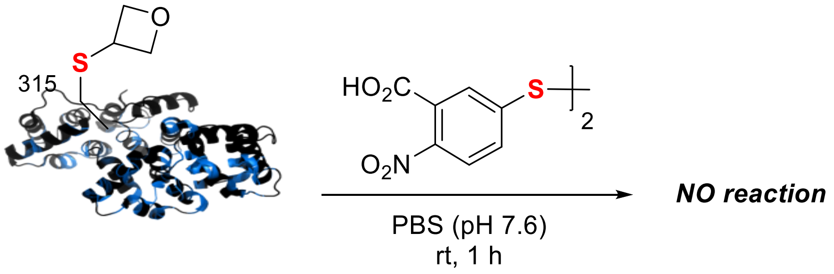

All manipulations were carried out at room temperature. A 10 µL aliquot of AnxV-3-*S*-Ox (**3**) (*ca.* 0.16 mg/mL, 0.045 nmol) in PBS buffer at pH 7.6 was transferred to a 0.5 mL eppendorf tube. Ellman's reagent (0.5 µL of a 14 mg/mL stock solution in H_2O, 17.66 nmol) was added and the resulting mixture vortexed for 30 seconds. After 1 h of additional shaking, a 5 µL aliquot was analyzed by LC–MS (5 µL aliquot diluted by 5 µL of PBS buffer at pH 7.6) and starting protein (**3**) (calculated mass, 35862; observed mass, 35859) was detected unaltered.

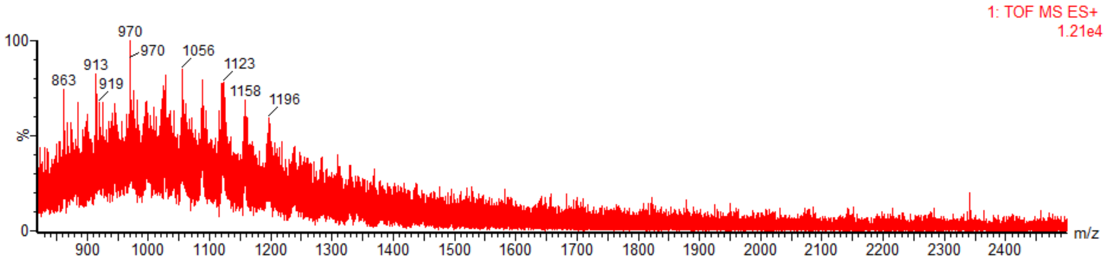

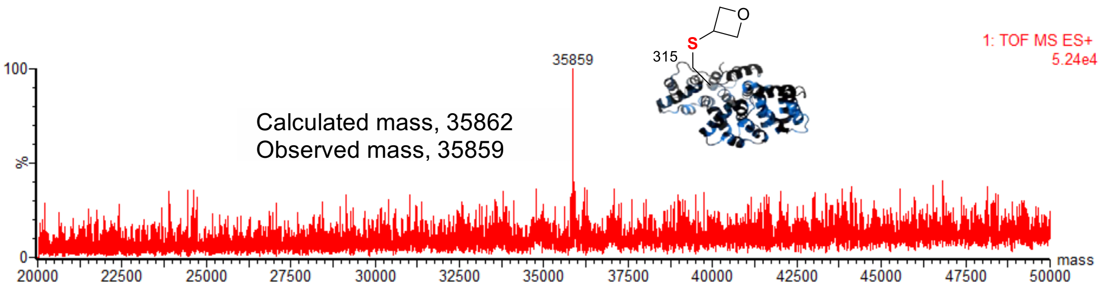

Figure S53. ESI–MS of the incubation of AnxV-3-*S*-Ox (**3**) with Ellman's reagent

Reaction of AnxV with 3,3-bis(bromomethyl)oxetane (4)

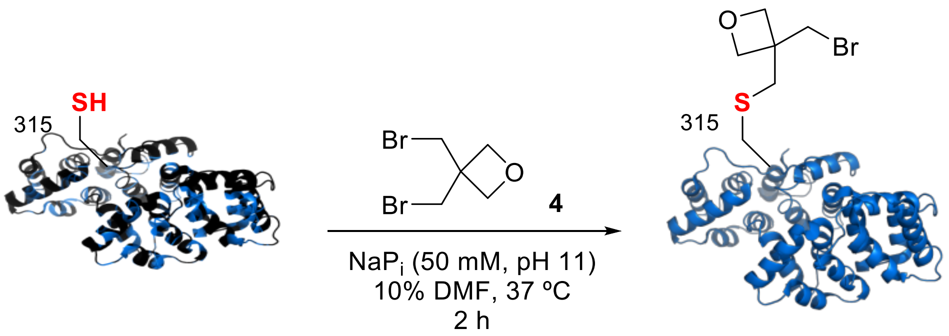

AnxV was prepared as a 0.25 mg/mL solution in 50 mM sodium phosphate buffer at pH 11 and 25 µL (0.175 nmol) were transferred to a 0.5 mL eppendorf tube. 3,3-Bis(bromomethyl)oxetane (**4**) (0.27 mg, 1047.3 nmol) in DMF (3 µL) was added and the resulting mixture vortexed for 30 seconds. After 2 h of additional shaking at 37 ºC, a 3 µL aliquot was analyzed by LC–MS (3 µL aliquot diluted by 7 µL of 50 mM sodium phosphate buffer at pH 11) and complete conversion to AnxV-BrCH$_2$-*S*-Ox (**6**) (calculated mass, 35969; observed mass, 35967) was observed. Small molecules were removed from the reaction mixture by loading the sample onto a Zeba™ Spin desalting column (ThermoFisher Scientific) previously equilibrated with PBS buffer at pH 7.6. The sample was eluted by centrifugation (2 min, 1500xg). The protein sample (0.24 mg/mL by Bradford assay) was flash frozen with liquid nitrogen and stored at –20 ºC.

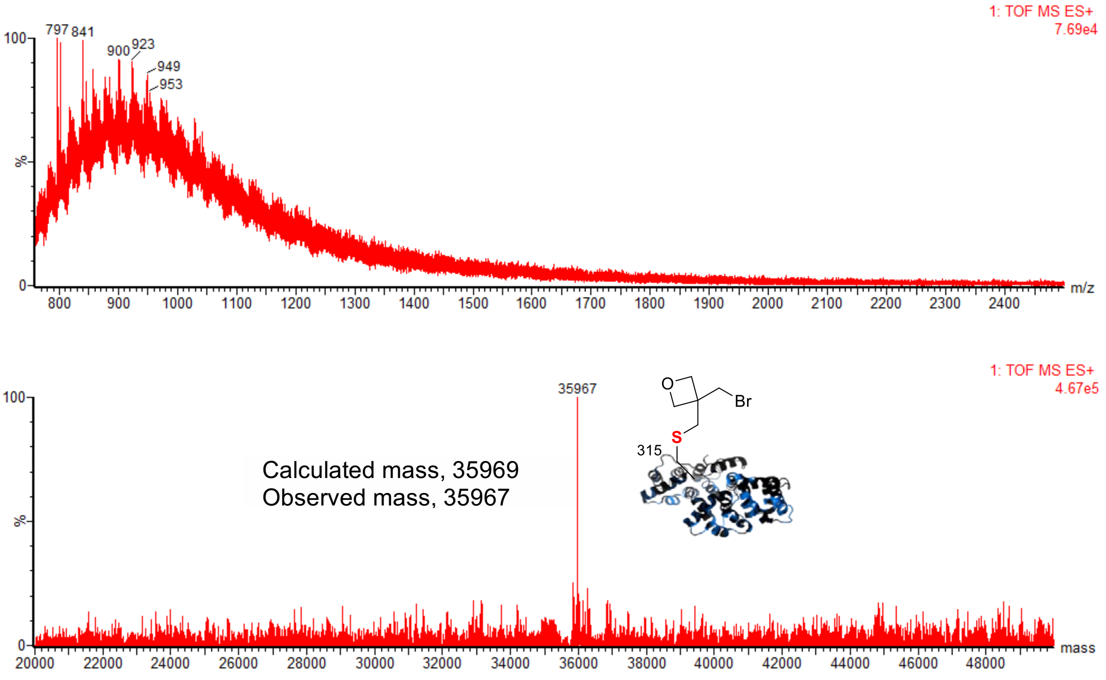

Figure S54. ESI–MS of the reaction of AnxV with 3,3-bis(bromomethyl)oxetane (**4**)

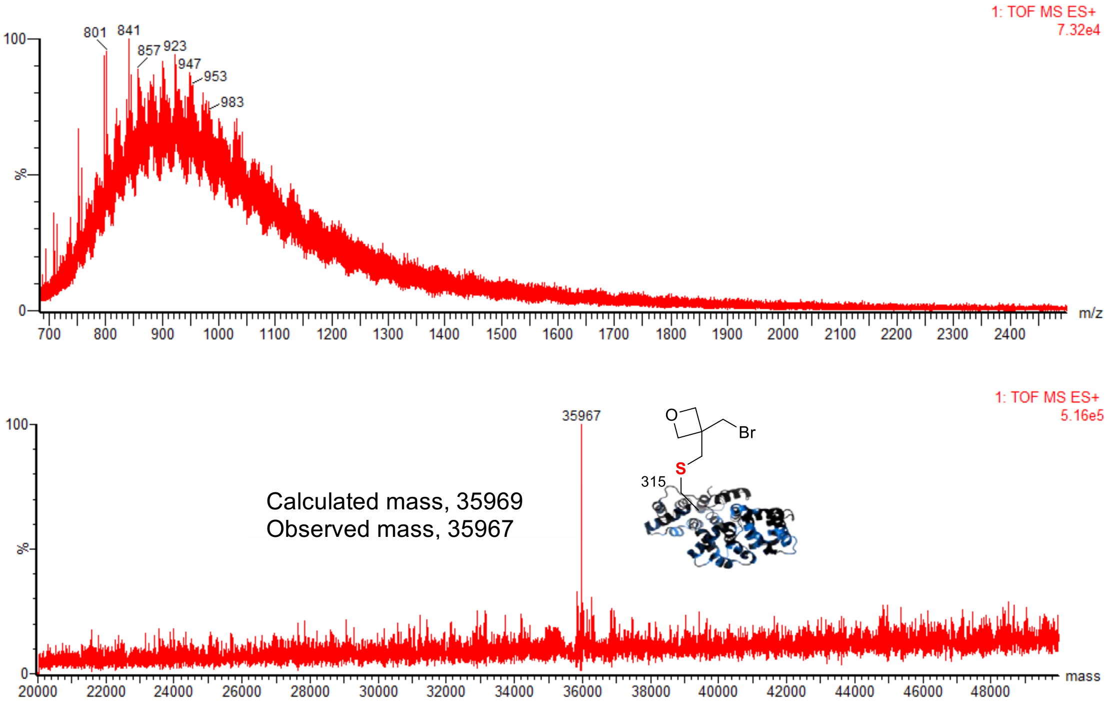

Figure S55. ESI–MS of AnxV-BrCH_2-*S*-Ox (**6**) after storage at –20 ºC for >9 months in PBS (pH 7.6)

Control: incubation of AnxV-BrCH$_2$-*S*-Ox (6) with Ellman's reagent

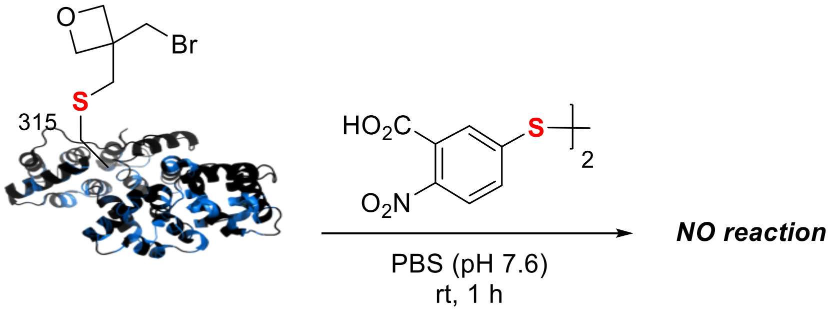

All manipulations were carried out at room temperature. A 10 µL aliquot of AnxV-BrCH$_2$-*S*-Ox (**6**) (*ca.* 0.24 mg/mL, 0.067 nmol) in PBS buffer at pH 7.6 was transferred to a 0.5 mL eppendorf tube. Ellman's reagent (1 µL of a 14 mg/mL stock solution in H_2O, 35.33 nmol) was added and the resulting mixture vortexed for 30 seconds. After 1 h of additional shaking, a 5 µL aliquot was analyzed by LC–MS (5 µL aliquot diluted by 5 µL of PBS buffer at pH 7.6) and starting protein (**6**) (calculated mass, 35969; observed mass, 35969) was detected unaltered.

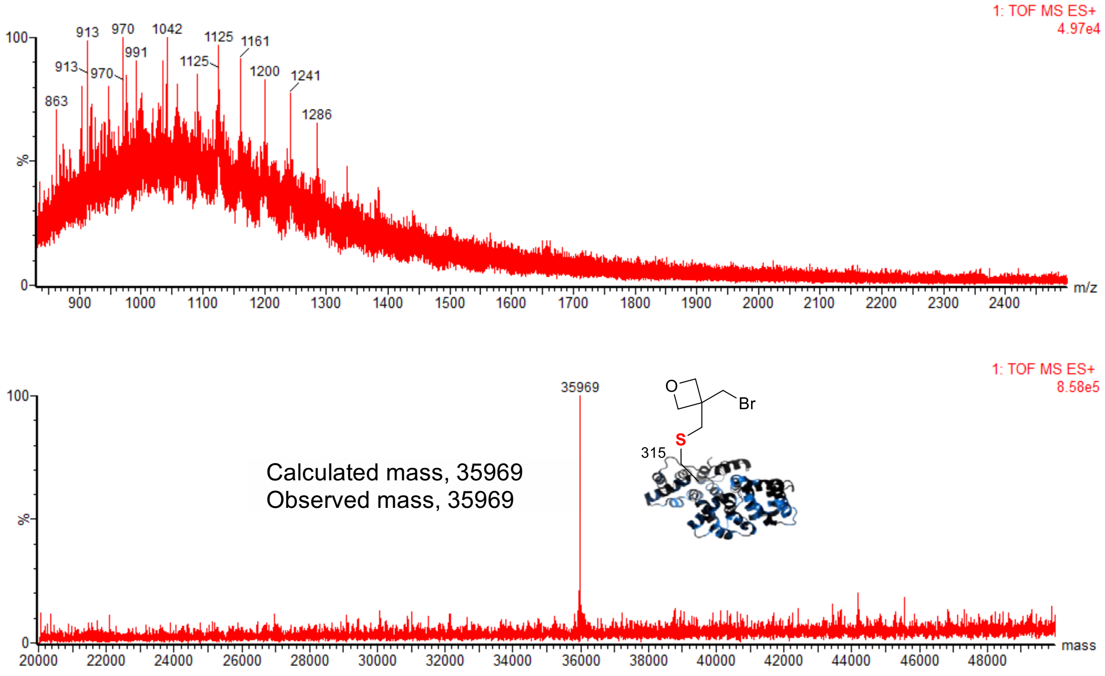

Figure S56. ESI–MS of the incubation of AnxV-BrCH$_2$-*S*-Ox (**6**) with Ellman's reagent

Reaction of AnxV with 3-(bromomethyl)oxetane (9)

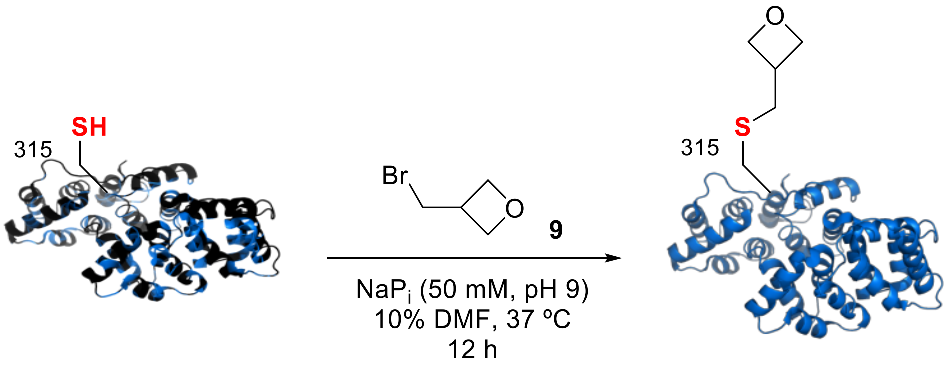

AnxV was prepared as a 0.25 mg/mL solution in 50 mM sodium phosphate buffer at pH 9 and 15 µL (0.105 nmol) were transferred to a 0.5 mL eppendorf tube. 3-(Bromomethyl)oxetane (**9**) (1.5 µL of a 0.4 mg stock solution in 723 µL DMF, 5.2 nmol) was added and the resulting mixture vortexed for 30 seconds. After 12 h of additional shaking at 37 ºC, a 3 µL aliquot was analyzed by LC–MS (3 µL aliquot diluted by 7 µL of 50 mM sodium phosphate buffer at pH 9) and complete conversion to AnxV-SCH$_2$-Ox (**11**) (calculated mass, 35876; observed mass, 35874) was observed. Small molecules were removed from the reaction mixture by loading the sample onto a Zeba™ Spin desalting column (ThermoFisher Scientific) previously equilibrated with PBS buffer at pH 7.6. The sample was eluted by centrifugation (2 min, 1500xg). The protein sample (0.24 mg/mL by Bradford assay) was flash frozen with liquid nitrogen and stored at –20 ºC.

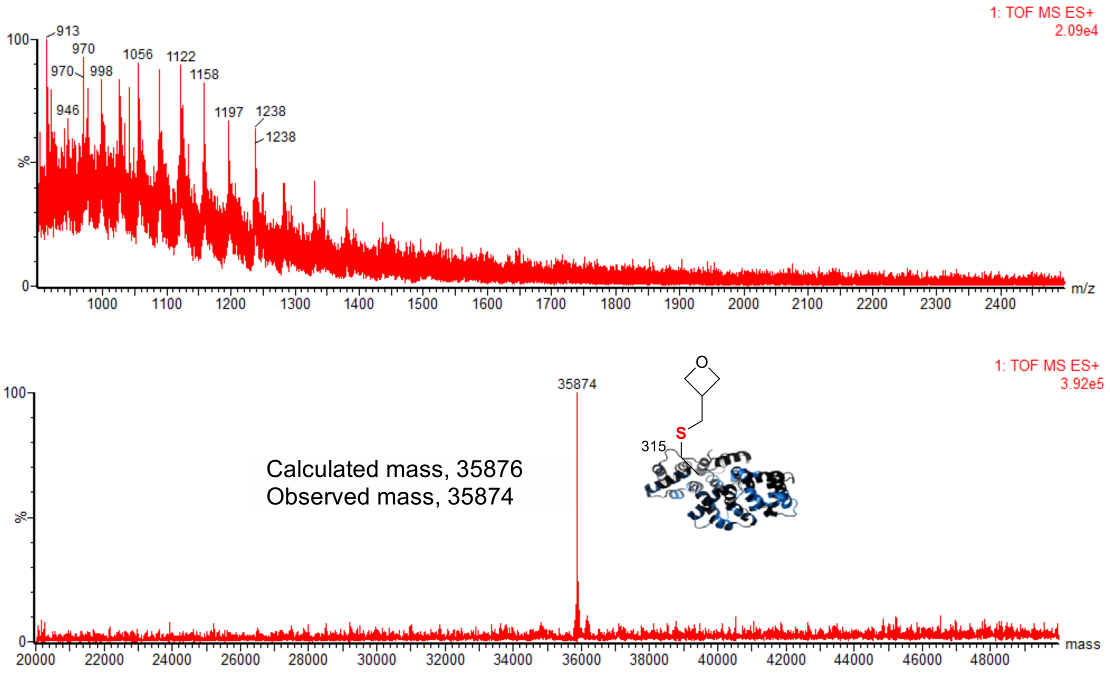

Figure S57. ESI–MS of the reaction of AnxV with 3-(bromomethyl)oxetane (**9**)

Control: incubation of AnxV-SCH$_2$-Ox (11) with Ellman's reagent

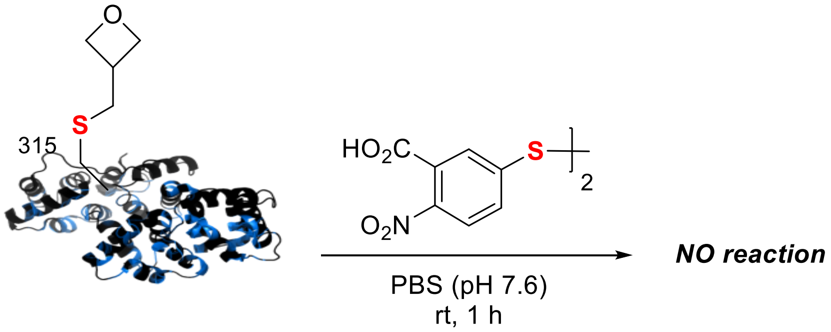

All manipulations were carried out at room temperature. A 10 µL aliquot of AnxV-SCH$_2$-Ox (**11**) (*ca.* 0.24 mg/mL, 0.067 nmol) in PBS buffer at pH 7.6 was transferred to a 0.5 mL eppendorf tube. Ellman's reagent (1 µL of a 14 mg/mL stock solution in H_2O, 35.33 nmol) was added and the resulting mixture vortexed for 30 seconds. After 1 h of additional shaking, a 5 µL aliquot was analyzed by LC–MS (5 µL aliquot diluted by 5 µL of PBS buffer at pH 7.6) and starting protein (**11**) (calculated mass, 35876; observed mass, 35874) was detected unaltered.

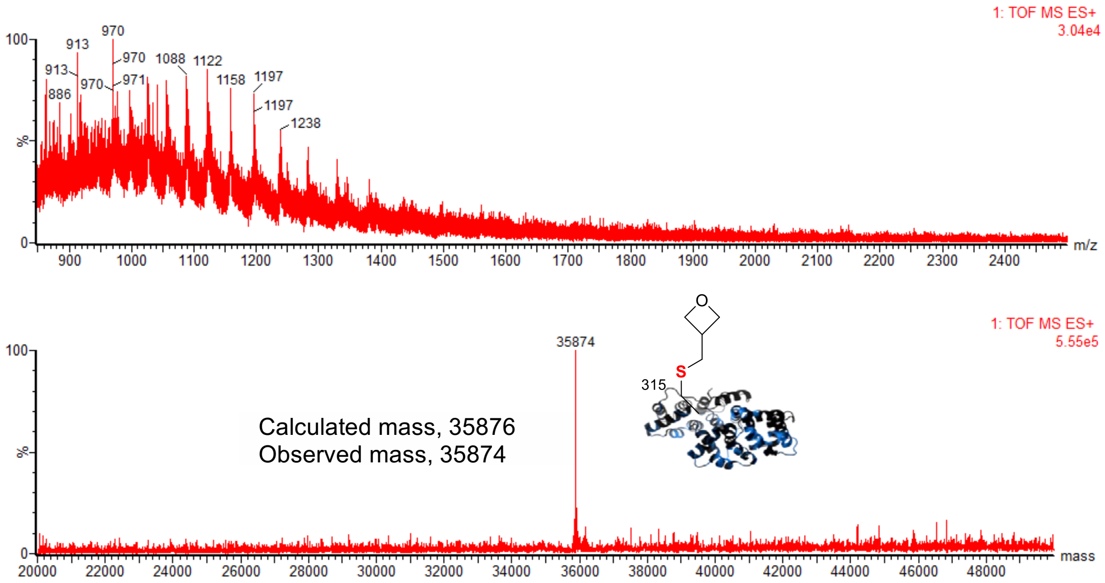

Figure S58. ESI–MS of the incubation of AnxV-SCH$_2$-Ox (**11**) with Ellman's reagent

Reaction of AnxV-SCH$_2$-Ox (11) with βGalSNa

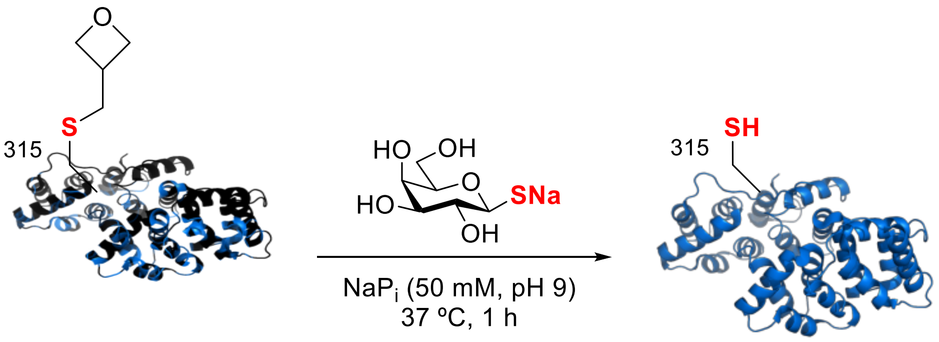

AnxV-SCH$_2$-Ox (**11**) was prepared as a 0.25 mg/mL solution in 50 mM sodium phosphate buffer at pH 9 and 10 μL (0.070 nmol) were transferred to a 0.5 mL eppendorf tube. βGalSNa (2 μL of a 0.5 mg stock solution in 655 μL H_2O, 7.0 nmol) was added at room temperature and the resulting mixture vortexed for 30 seconds. After 1 h of additional shaking at 37 ºC, a 3 μL aliquot was analyzed by LC–MS (3 μL aliquot diluted by 7 μL of 50 mM sodium phosphate buffer at pH 9) and complete conversion to AnxV (calculated mass, 35806; observed mass, 35806) was observed.

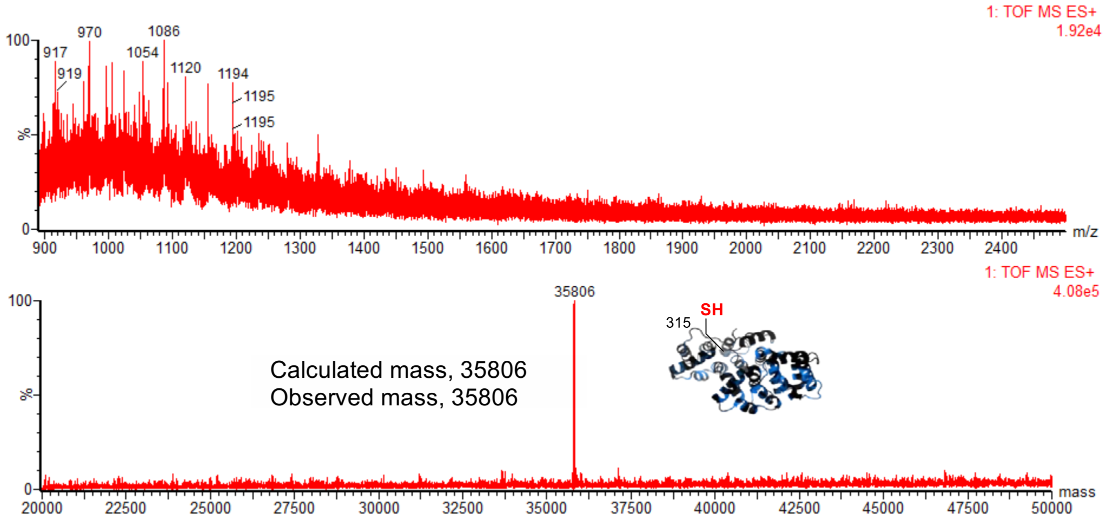

Figure S59. ESI–MS of the reaction of AnxV-SCH$_2$-Ox (**11**) with βGalSNa

Control (incubation with Ellman's reagent): to the above reaction mixture, Ellman's reagent (3 μL of a 20 mg/mL stock solution in H_2O, 50.45 nmol) was added at room temperature and the resulting mixture vortexed for 30 seconds. After 4 h of additional shaking at room temperature, a 3 μL aliquot was analyzed by LC–MS (3 μL aliquot diluted by 7 μL of 50 mM sodium phosphate buffer at pH 9) and complete conversion

to the expected Ellman's product (calculated mass, 36003; observed mass, 36003) was observed.

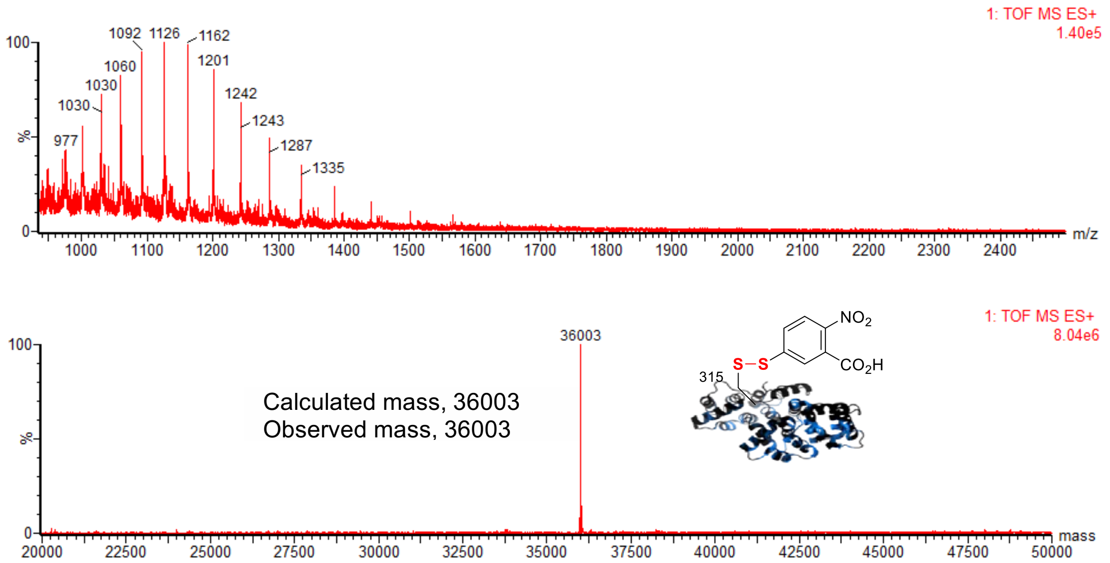

Figure S60. ESI–MS of the reaction of AnxV with Ellman's reagent

Reaction of AnxV-SCH$_2$-Ox (11) with TCEP·HCl

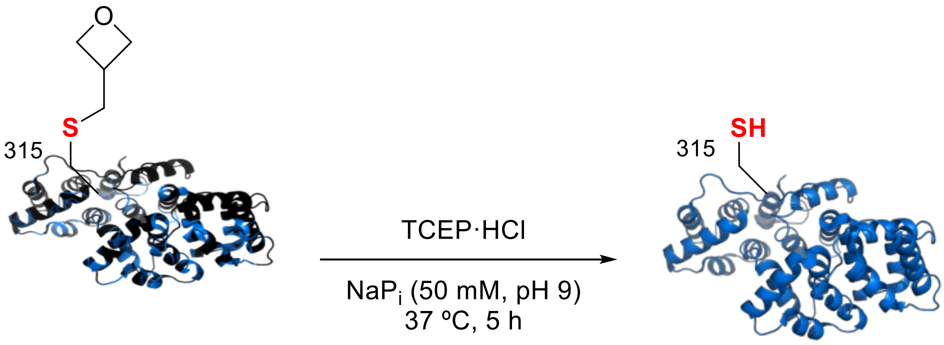

AnxV-SCH$_2$-Ox (**11**) was prepared as a 0.25 mg/mL solution in 50 mM sodium phosphate buffer at pH 9 and 10 µL (0.070 nmol) were transferred to a 0.5 mL eppendorf tube. TCEP·HCl (2 µL of an 11 mg/mL stock solution in H_2O, 678.0 nmol) was added at room temperature and the resulting mixture vortexed for 30 seconds. After 5 h of additional shaking at 37 ºC, a 3 µL aliquot was analyzed by LC–MS (3 µL aliquot diluted by 7 µL of 50 mM sodium phosphate buffer at pH 9) and complete conversion to AnxV (calculated mass, 35806; observed mass, 35806) was observed.

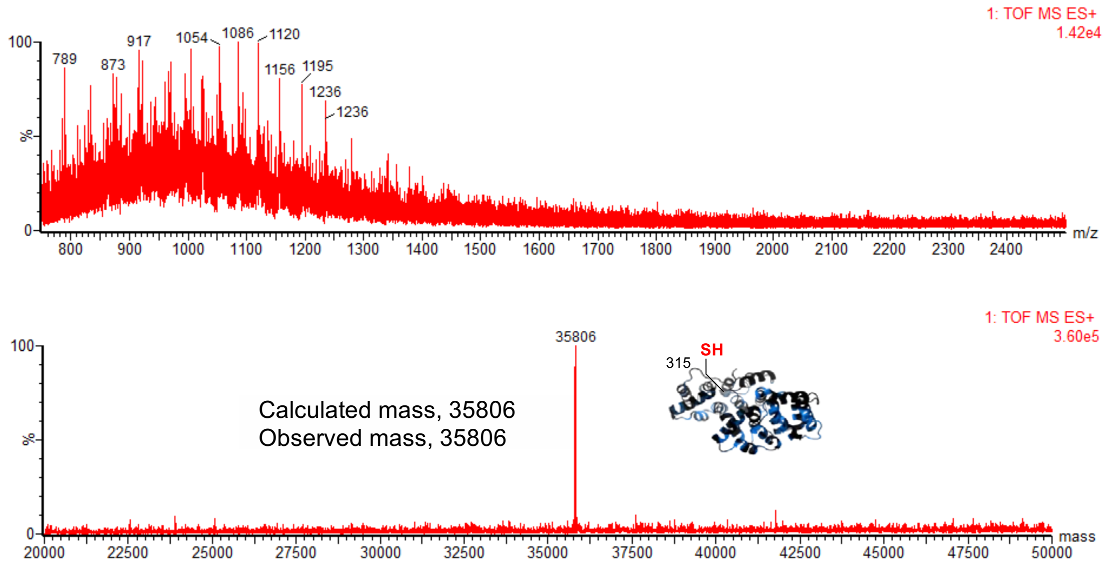

Figure S61. ESI–MS of the reaction of AnxV-SCH_2-Ox (**11**) with TCEP·HCl

Control: reaction of AnxV-Sac (S3) with $BnNH_2$

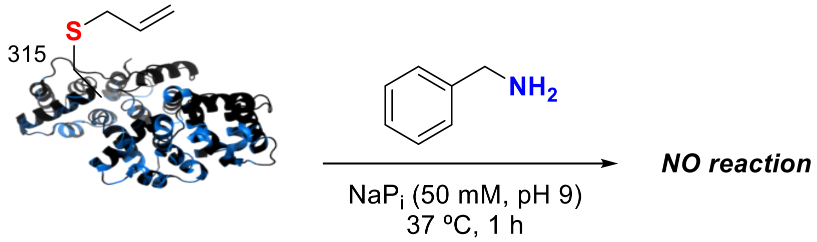

AnxV-Sac (**S3**)[11] was prepared as a 0.25 mg/mL solution in 50 mM sodium phosphate buffer at pH 9 and 20 µL (0.139 nmol) were transferred to a 0.5 mL eppendorf tube. $BnNH_2$ (2 µL of a 1 mg/mL stock solution in H_2O, 13.9 nmol) was added at room temperature and the resulting mixture vortexed for 30 seconds. After 1 h of additional shaking at 37 ºC, a 3 µL aliquot was analyzed by LC–MS (3 µL aliquot diluted by 7 µL of 50 mM sodium phosphate buffer at pH 9) and starting protein (**S3**) (calculated mass, 35846; observed mass, 35846) was detected unaltered.

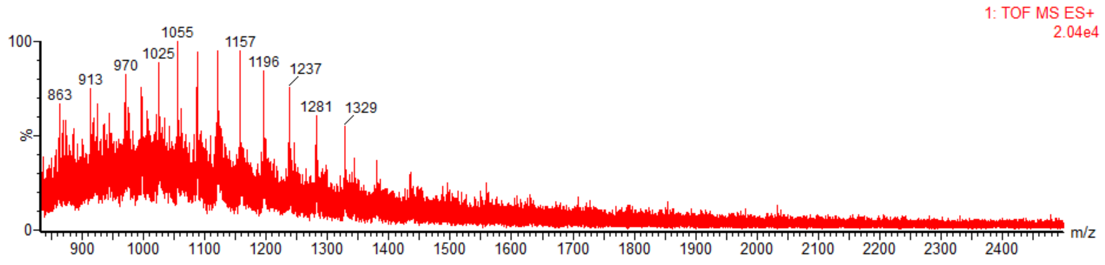

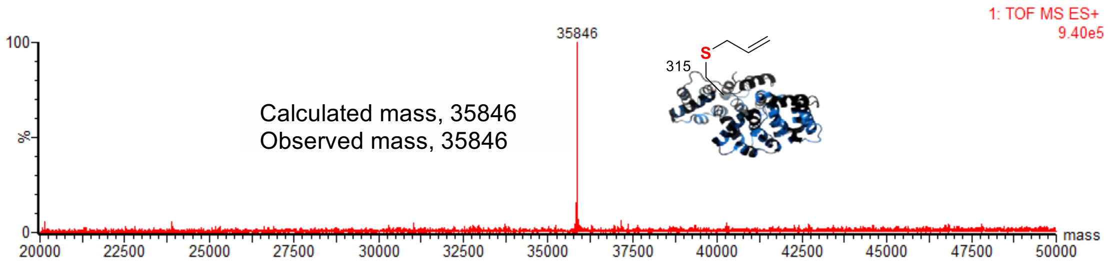

Figure S62. ESI–MS of the reaction of AnxV-Sac (**S3**) with $BnNH_2$

Control: reaction of AnxV-Sac (S3) with βGalSNa

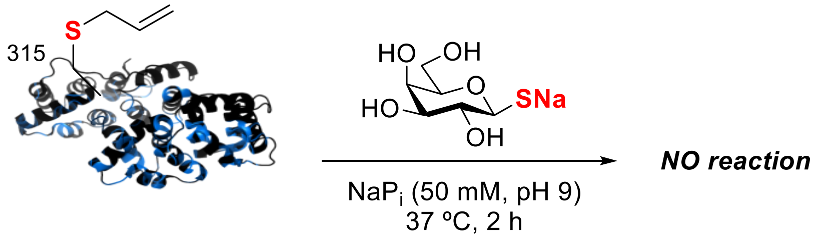

AnxV-Sac (**S3**)[11] was prepared as a 0.25 mg/mL solution in 50 mM sodium phosphate buffer at pH 9 and 20 μL (0.139 nmol) were transferred to a 0.5 mL eppendorf tube. βGalSNa (2 μL of a 0.5 mg stock solution in 330 μL H_2O, 13.9 nmol) was added at room temperature and the resulting mixture vortexed for 30 seconds. After 2 h of additional shaking at 37 ºC, a 3 μL aliquot was analyzed by LC–MS (3 μL aliquot diluted by 7 μL of 50 mM sodium phosphate buffer at pH 9) and starting protein (**S3**) (calculated mass, 35846; observed mass, 35846) was detected unaltered.

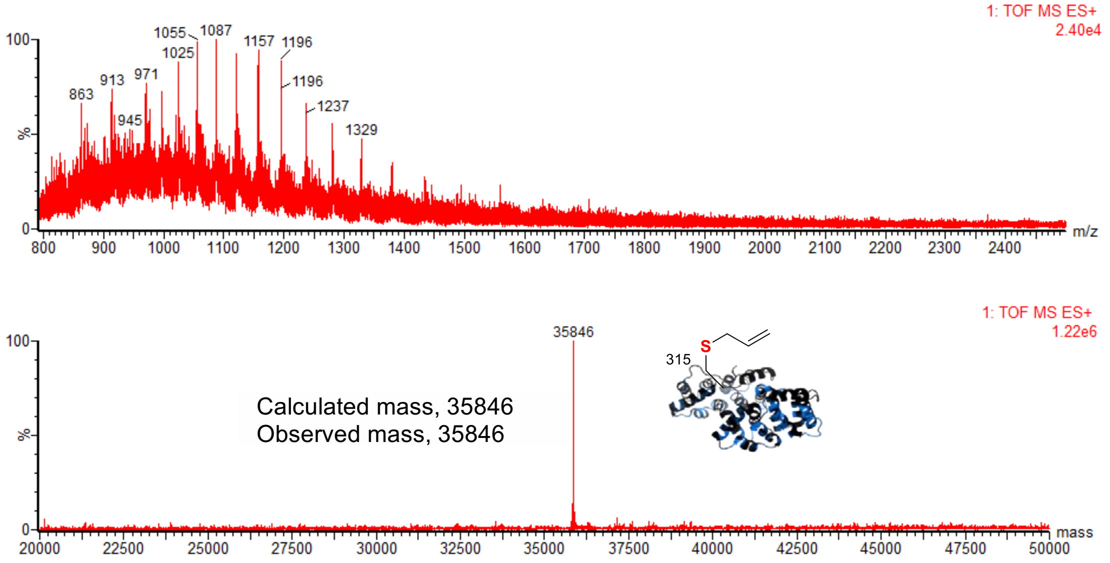

Figure S63. ESI–MS of the reaction of AnxV-Sac (**S3**) with βGalSNa

Recombinant human serum albumin (rHSA)

Sequence of rHSA C34 (modified residue highlighted)

DAHKSEVAHRFKDLGEENFKALVLIAFAQYLQQCPFEDHVKLVNEVTEFAKT
CVADESAENCDKSLHTLFGDKLCTVATLRETYGEMADCCAKQEPERNECFL
QHKDDNPNLPRLVRPEVDVMCTAFHDNEETFLKKYLYEIARRHPYFYAPELL
FFAKRYKAAFTECCQAADKAACLLPKLDELRDEGKASSAKQRLKCASLQKF
GERAFKAWAVARLSQRFPKAEFAEVSKLVTDLTKVHTECCHGDLLECADDR
ADLAKYICENQDSISSKLKECCEKPLLEKSHCIAEVENDEMPADLPSLAADFV
ESKDVCKNYAEAKDVFLGMFLYEYARRHPDYSVVLLLRLAKTYETTLEKCCA
AADPHECYAKVFDEFKPLVEEPQNLIKQNCELFEQLGEYKFQNALLVRYTKK
VPQVSTPTLVEVSRNLGKVGSKCCKHPEAKRMPCAEDYLSVVLNQLCVLHE
KTPVSDRVTKCCTESLVNRRPCFSALEVDETYVPKEFNAETFTFHADICTLS
EKERQIKKQTALVELVKHKPKATKEQLKAVMDDFAAFVEKCCKADDKETCF
AEEGKKLVAASQAALGL

Calculated average isotopic mass = 66438.21 Da (17 internal disulfides)

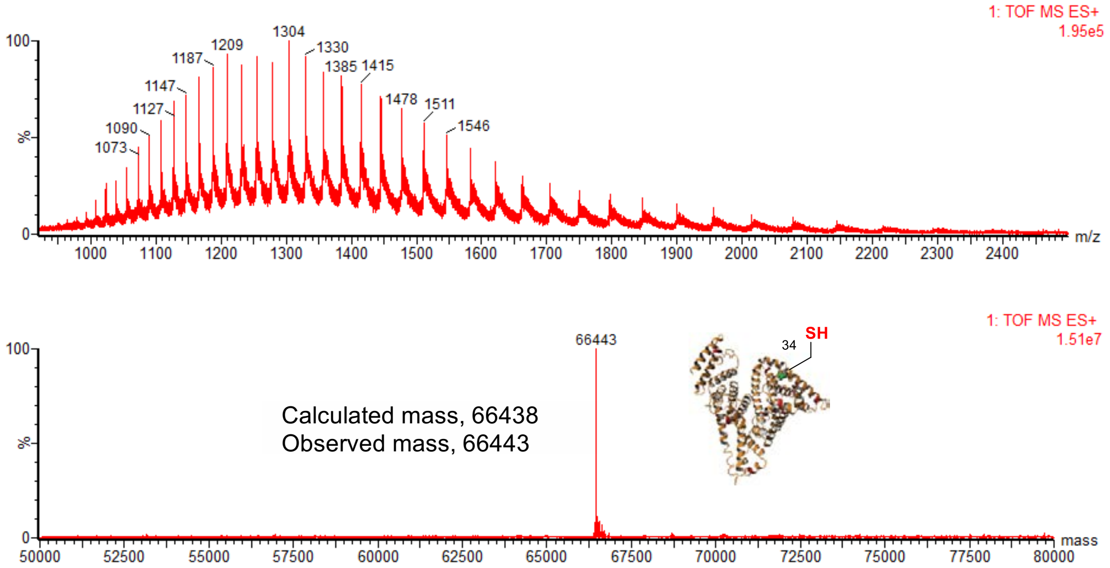

Figure S64. ESI–MS spectrum of rHSA

Control: reaction of rHSA with Ellman's reagent

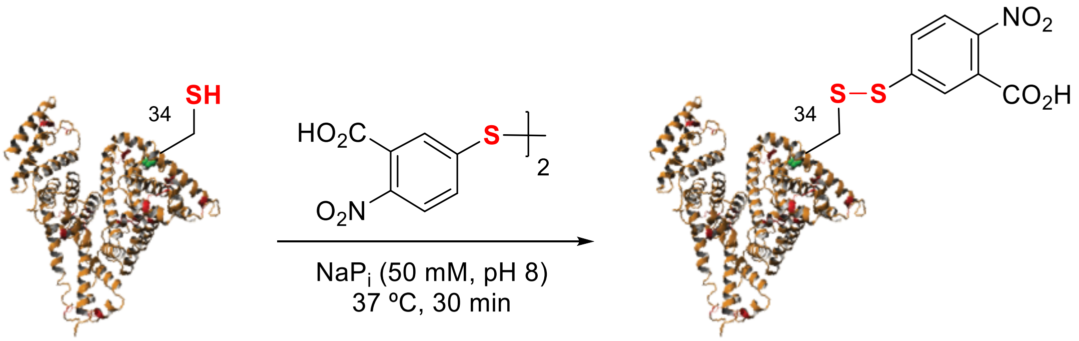

A 10 µL aliquot of rHSA (1 mg/mL, 0.151 nmol) in 50 mM sodium phosphate buffer at pH 8 was transferred to a 0.5 mL eppendorf tube. Ellman's reagent (3 µL of a 14 mg/mL stock solution in H_2O, 105.98 nmol) was added at room temperature and the resulting mixture vortexed for 30 seconds. After 30 min of additional shaking at 37 ºC, a 3 µL aliquot was analyzed by LC–MS (3 µL aliquot diluted by 7 µL of 50 mM sodium phosphate buffer at pH 8) and complete conversion to the expected Ellman's product (calculated mass, 66636; observed mass, 66639) was observed.

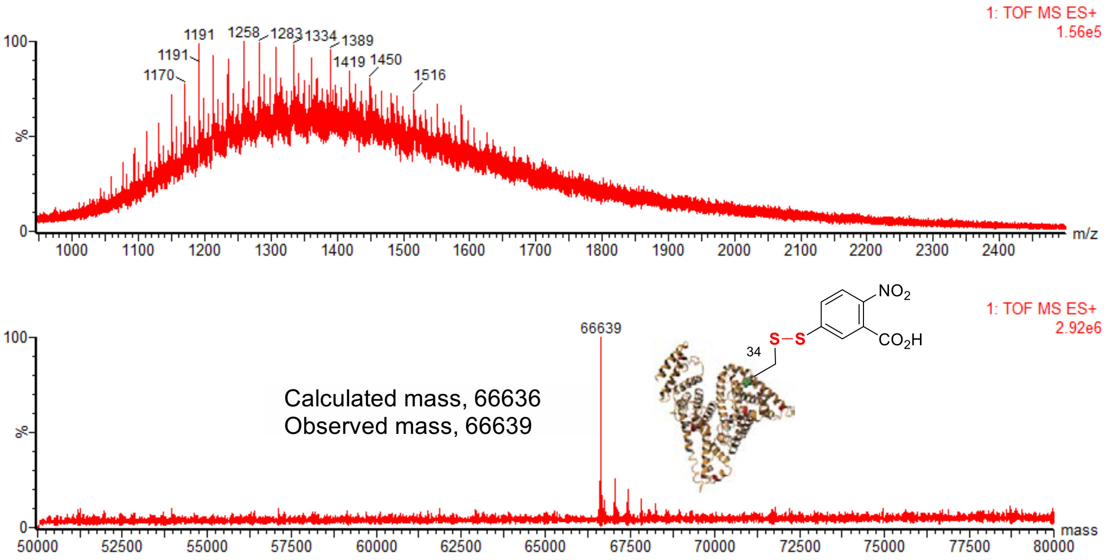

Figure S65. ESI–MS of the reaction of rHSA with Ellman's reagent

Reaction of rHSA with 3-(bromomethyl)oxetane (9)

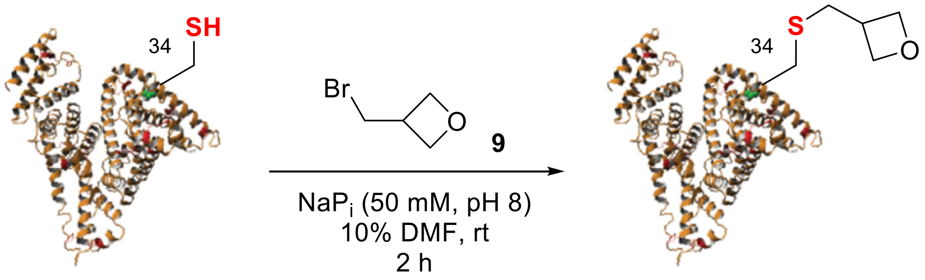

All manipulations were carried out at room temperature. A 100 µL aliquot of rHSA (1 mg/mL, 1.51 nmol) in 50 mM sodium phosphate buffer at pH 8 was transferred to a 0.5 mL eppendorf tube. 3-(Bromomethyl)oxetane (**9**) (11 µL of a 0.12 mg stock solution in 110 µL DMF, 376.3 nmol) was added and the resulting mixture vortexed for 30 seconds. After 2 h of additional shaking, a 3 µL aliquot was analyzed by LC–MS (3 µL aliquot diluted by 7 µL of 50 mM sodium phosphate buffer at pH 8) and 25% conversion to rHSA-SCH$_2$-Ox (**12**) (calculated mass, 66508; observed mass, 66513) was observed.

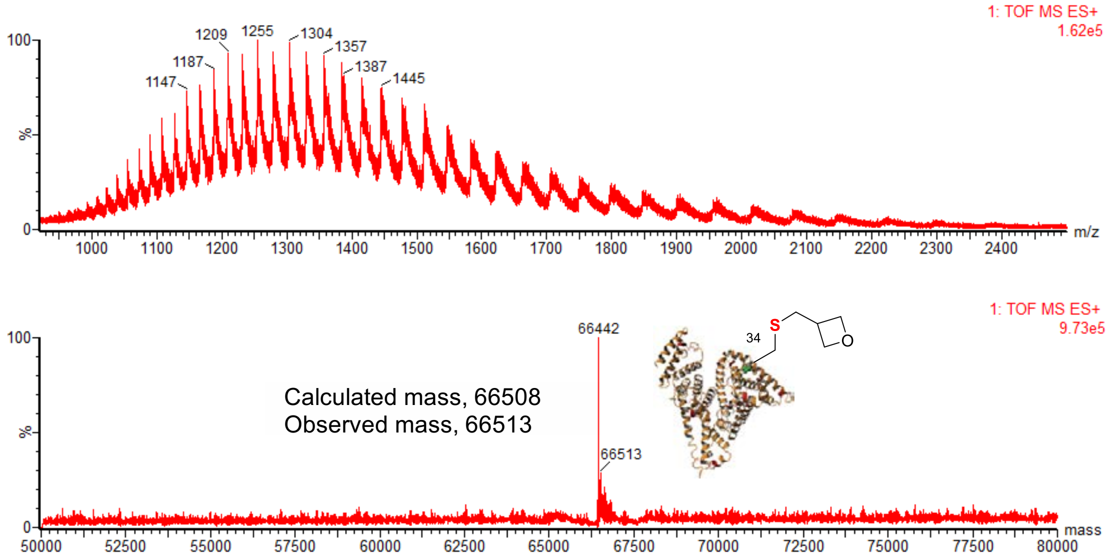

Figure S66. ESI–MS of the reaction of rHSA with 3-(bromomethyl)oxetane (**9**)

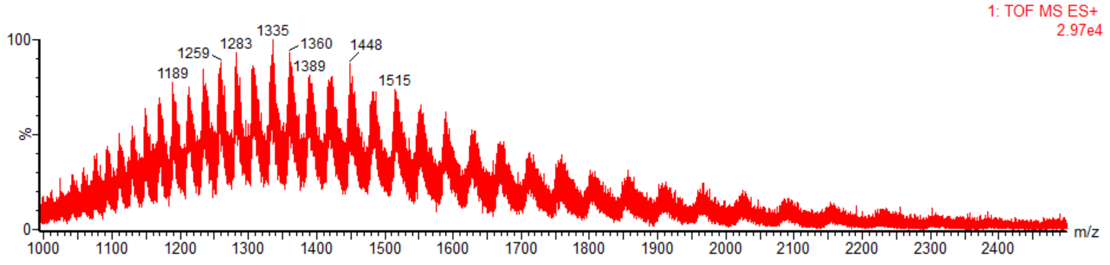

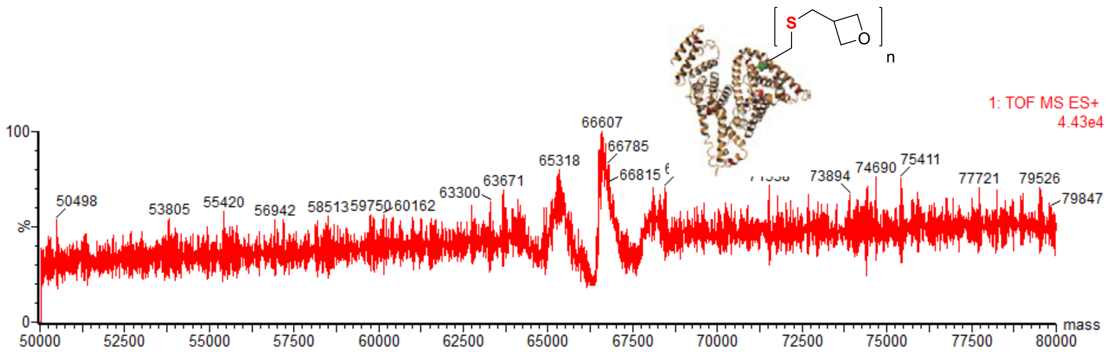

Figure S67. ESI–MS of the reaction of rHSA with 3-(bromomethyl)oxetane (**9**) (>50 equiv. and 14 h at 37 ºC)

Trastuzumab (Thiomab™ 4D5 LC-V205C)

Trastuzumab was reduced, purified, and re-oxidized as described.[14]

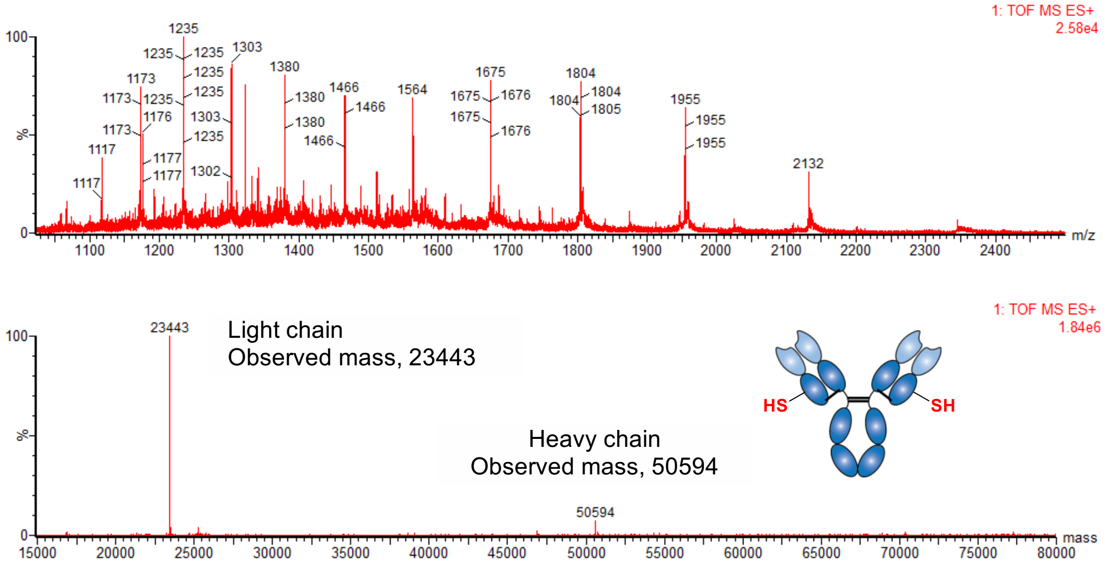

Figure S68. ESI–MS spectrum of reduced Trastuzumab (treated with TCEP·HCl for analysis)

Reaction of Trastuzumab with 3-(bromomethyl)oxetane (9)

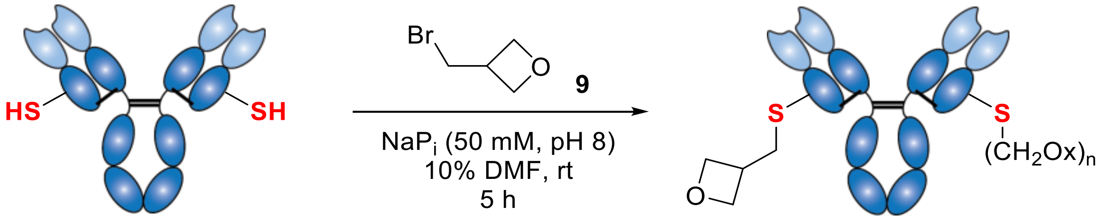

All manipulations were carried out at room temperature. A 50 µL aliquot of Trastuzumab (1 mg/mL, 0.355 nmol) in 50 mM sodium phosphate buffer at pH 8 was transferred to a 0.5 mL eppendorf tube. 3-(Bromomethyl)oxetane (**9**) (5.5 µL of a 1.3

mg stock solution in 2.5 mL DMF, 17.8 nmol) was added and the resulting mixture vortexed for 30 seconds. After 5 h of additional shaking, a 3 μL aliquot was analyzed by LC–MS (3 μL aliquot diluted by 6 μL of 50 mM sodium phosphate buffer at pH 8 and 1 μL of TCEP·HCl, 1 mg/mL stock solution in H_2O) and ~50% conversion to Trastuzumab-(SCH_2-Ox)$_n$ (**13**) (Light chain; observed masses, 23563 and 23750) was observed. Small molecules were removed from the reaction mixture by loading the sample onto a Zeba™ Spin desalting column (ThermoFisher Scientific) previously equilibrated with PBS buffer at pH 7.6. The sample was eluted by centrifugation (2 min, 1500xg) and concentrated on a Vivaspin™ membrane concentrator (30 kDa molecular weight cut off). The protein sample (2.81 mg/mL by Bradford assay) was flash frozen with liquid nitrogen and stored at –20 ºC.

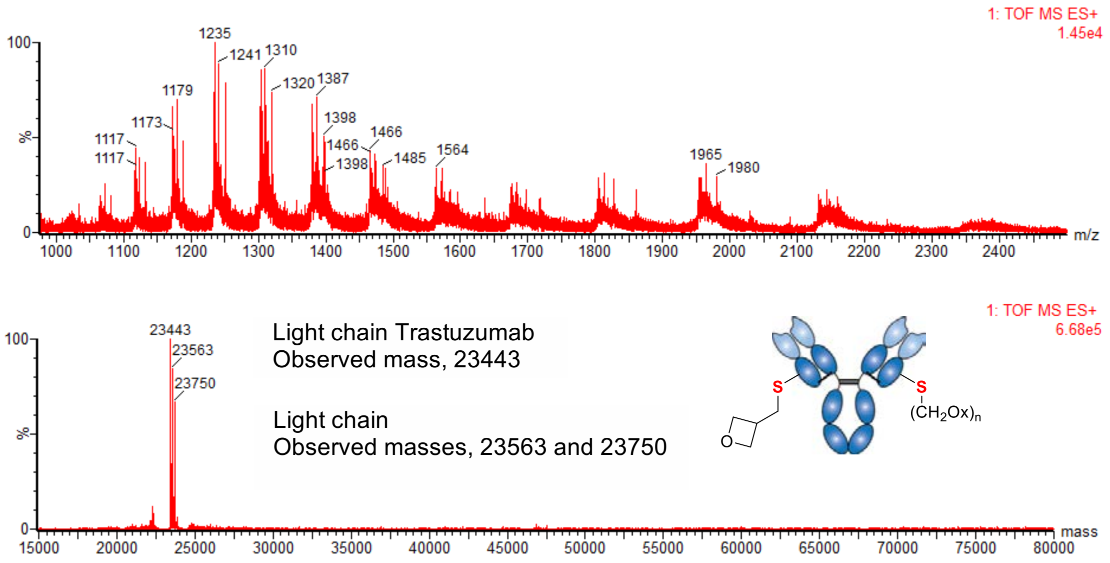

Figure S69. ESI–MS of the reaction of Trastuzumab with 3-(bromomethyl)oxetane (**9**) (treated with TCEP·HCl for analysis)

Reaction of Trastuzumab-(SCH_2-Ox)$_n$ (13) with TCEP·HCl

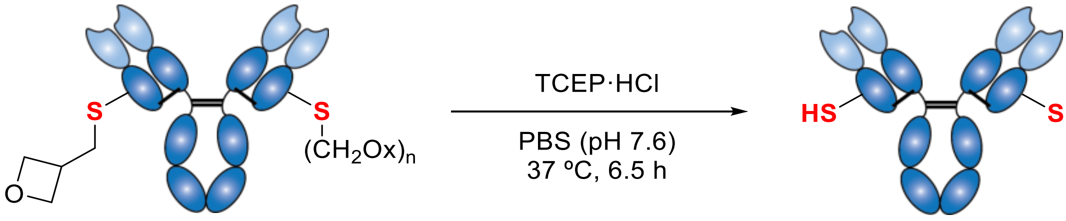

Trastuzumab-(SCH_2-Ox)$_n$ (**13**) was prepared as a *ca.* 1 mg/mL solution in PBS buffer at pH 7.6 and 13 μL (*ca.* 0.924 nmol) were transferred to a 0.5 mL eppendorf tube. TCEP·HCl (2 μL of a 13.2 mg/mL stock solution in H_2O, 92. 4 nmol) was added and

the resulting mixture vortexed for 30 seconds. After 6.5 h of additional shaking at 37 ºC, a 3 µL aliquot was analyzed by LC–MS (3 µL aliquot diluted by 7 µL of PBS buffer at pH 7.6) and complete conversion to Trastuzumab (Light chain; observed mass, 23447. Heavy chain; observed mass, 50601) was observed. Small molecules were removed from the reaction mixture by loading the sample onto a Zeba™ Spin desalting column (ThermoFisher Scientific) previously equilibrated with PBS buffer at pH 7.6. The sample was eluted by centrifugation (2 min, 1500xg) and concentrated on a Vivaspin™ membrane concentrator (30 kDa molecular weight cut off). The protein sample (2.85 mg/mL by Bradford assay) was flash frozen with liquid nitrogen and stored at –20 ºC.

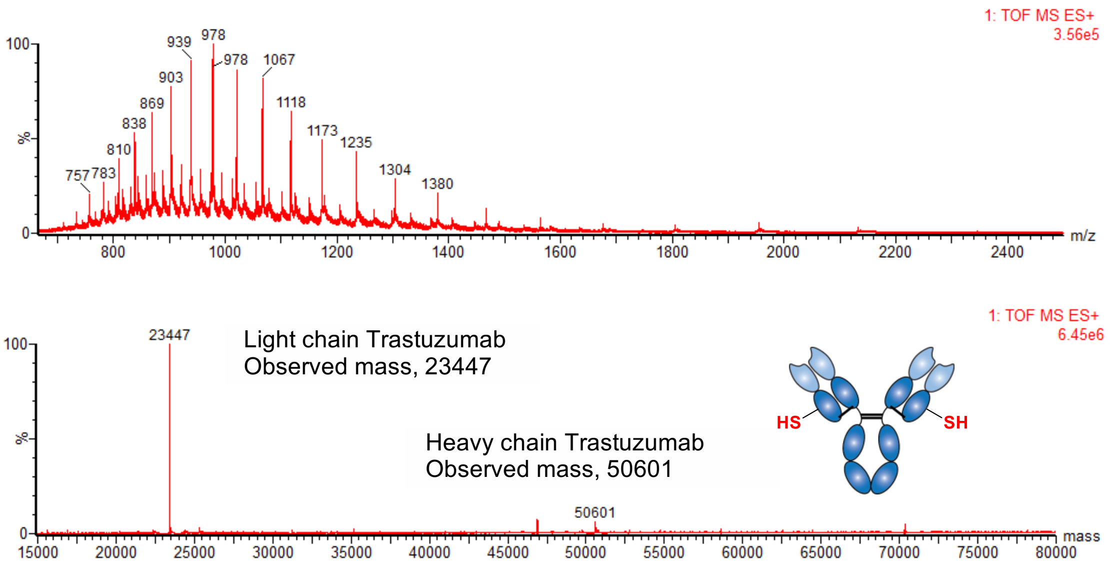

Figure S70. ESI–MS of the reaction of Trastuzumab-(SCH_2-Ox)$_n$ (**13**) with TCEP·HCl

Reaction of Trastuzumab-(SCH_2-Ox)$_n$ (13) with $BnNH_2$

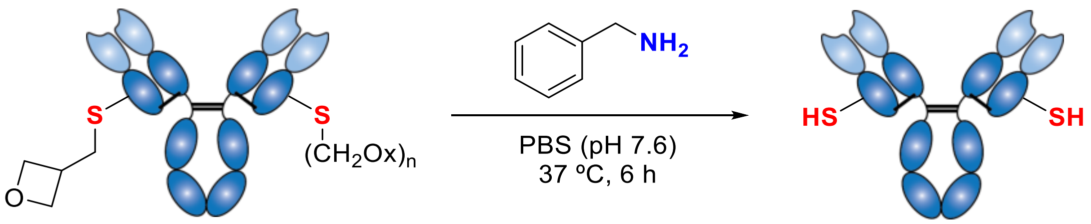

Trastuzumab-(SCH_2-Ox)$_n$ (**13**) was prepared as a *ca.* 1 mg/mL solution in PBS buffer at pH 7.6 and 13 µL (*ca.* 0.924 nmol) were transferred to a 0.5 mL eppendorf tube. $BnNH_2$ (2 µL of a 0.5 µL stock solution in 100 µL H_2O, 92. 4 nmol) was added and the resulting mixture vortexed for 30 seconds. After 6 h of additional shaking at 37

ºC, a 3 μL aliquot was analyzed by LC–MS (3 μL aliquot diluted by 6 μL of PBS buffer at pH 7.6 and 1 μL of TCEP·HCl, 1 mg/mL stock solution in H_2O) and complete conversion to Trastuzumab (Light chain; observed mass, 23442) was observed.

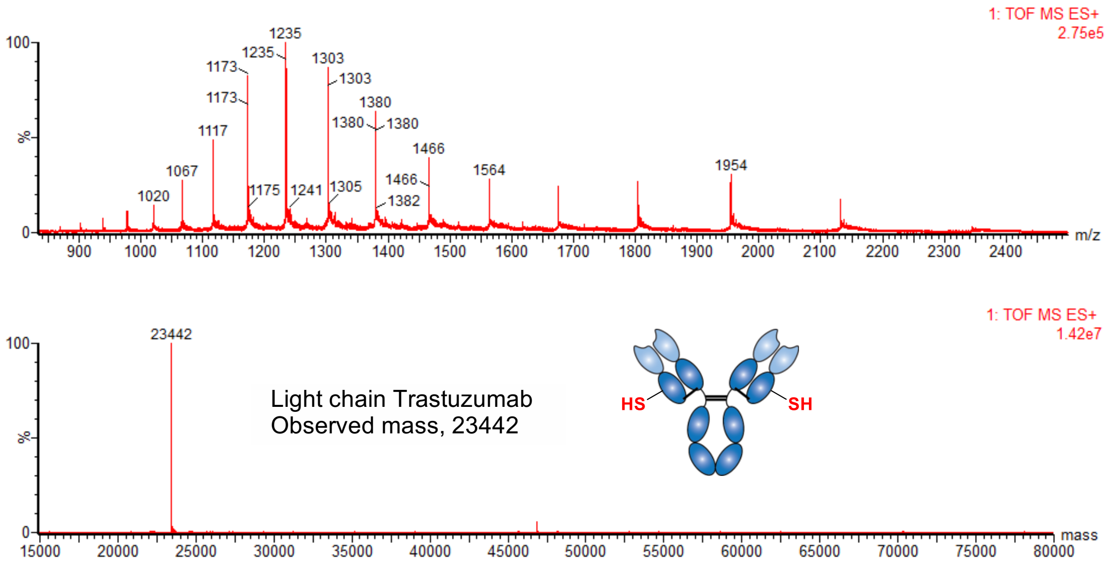

Figure S71. ESI–MS of the reaction of Trastuzumab-(SCH_2-Ox)$_n$ (**13**) with $BnNH_2$ (treated with TCEP·HCl for analysis)

Reaction of Trastuzumab-(SCH_2-Ox)$_n$ (13) with BME

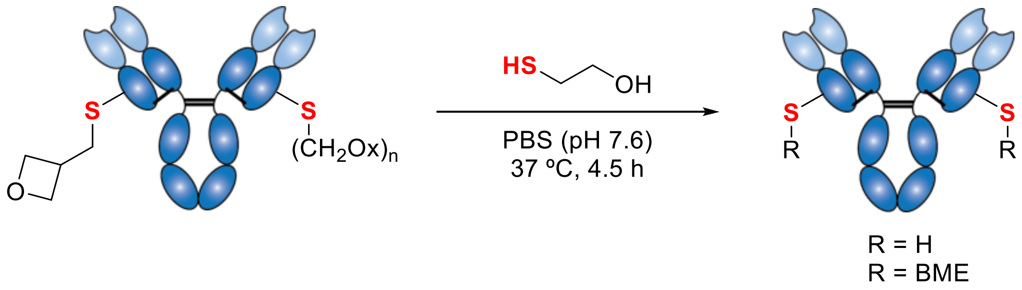

Trastuzumab-(SCH_2-Ox)$_n$ (**13**) was prepared as a *ca.* 1 mg/mL solution in PBS buffer at pH 7.6 and 13 μL (*ca.* 0.924 nmol) were transferred to a 0.5 mL eppendorf tube. BME (2 μL of a 0.5 μL stock solution in 154 μL H_2O, 92. 4 nmol) was added and the resulting mixture vortexed for 30 seconds. After 4.5 h of additional shaking at 37 ºC, a 3 μL aliquot was analyzed by LC–MS (3 μL aliquot diluted by 7 μL of PBS buffer at pH 7.6) and 40% conversion to Trastuzumab (Light chain; observed mass, 23443) together with 60% conversion to the corresponding mixed BME disulfide (Light chain; observed mass, 23519) was observed. When the same aliquot was treated with

1 μL of TCEP·HCl (1 mg/mL stock solution in H_2O) for analysis, complete conversion to Trastuzumab (Light chain; observed mass, 23443) was observed.

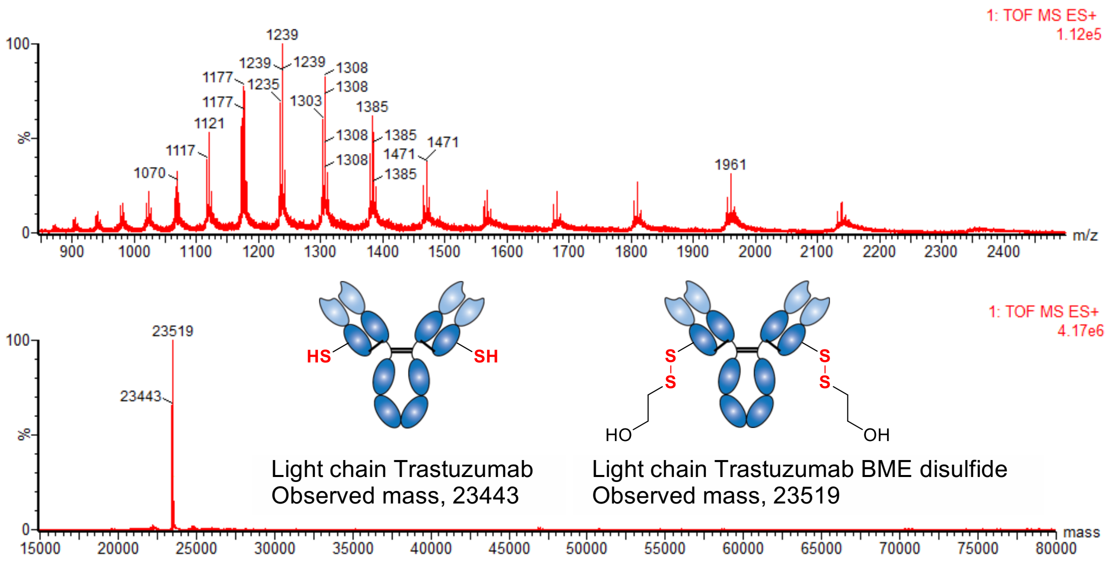

Figure S72. ESI–MS of the reaction of Trastuzumab-(SCH_2-Ox)$_n$ (**13**) with BME

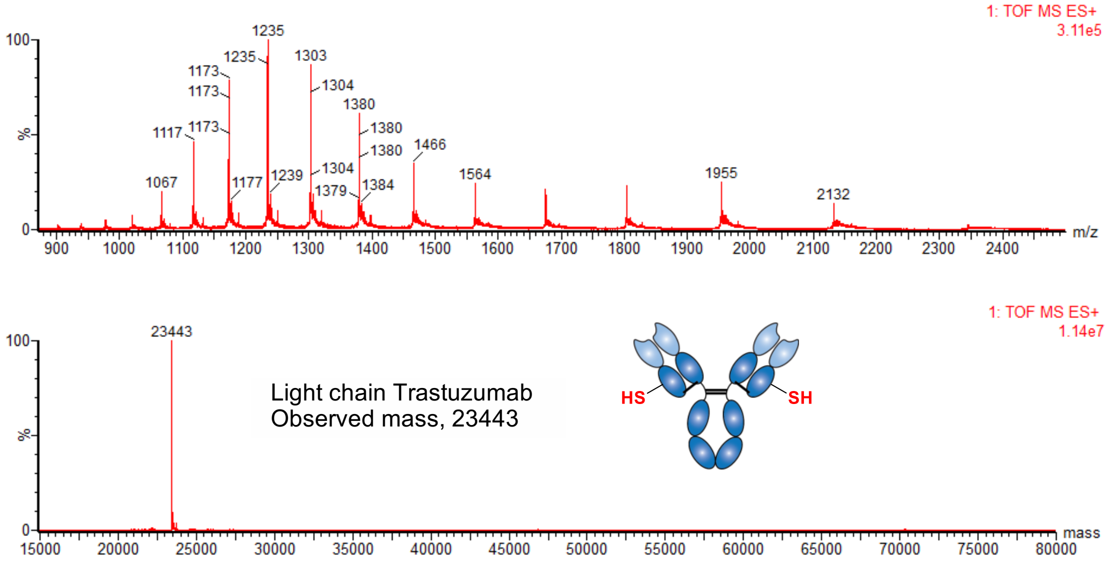

Figure S73. ESI–MS of the reaction of Trastuzumab-(SCH_2-Ox)$_n$ (**13**) with BME (treated with TCEP·HCl for analysis)

2.2. LC–MS/MS Analysis

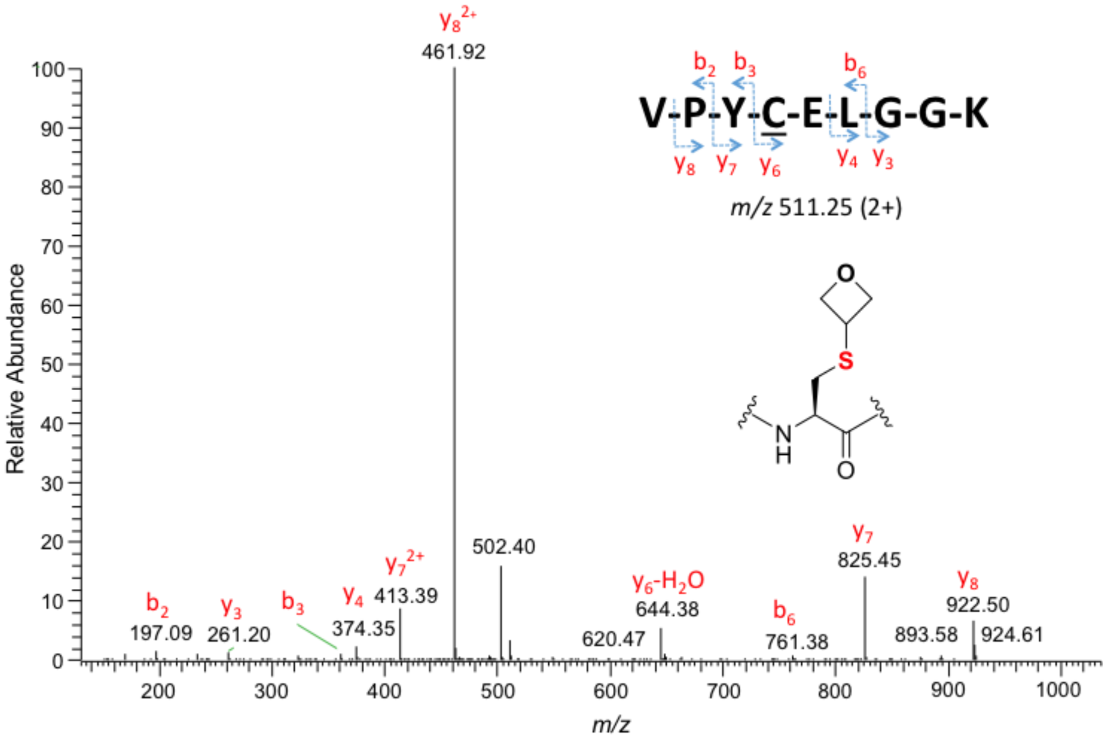

Figure S74. MS/MS spectrum of the *m/z* 511.25 (2+) ion of the tryptic peptide VPYCELGGK from C2Am 3-*S*-Ox (**2**), containing the 3-*S*-Oxetane modification at the original cysteine residue. The generated fragment ions are consistent with the mass of the modification

2.3. SDS Polyacrylamide Gel Electrophoresis (SDS-PAGE)

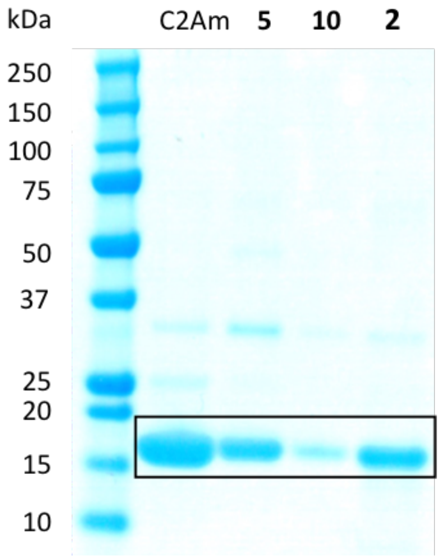

Figure S75. SDS-PAGE (MES SDS running buffer) analysis of C2Am and modified proteins C2Am 3-*S*-Ox (**2**), C2Am $BrCH_2$-*S*-Ox (**5**), and C2Am SCH_2-Ox (**10**)

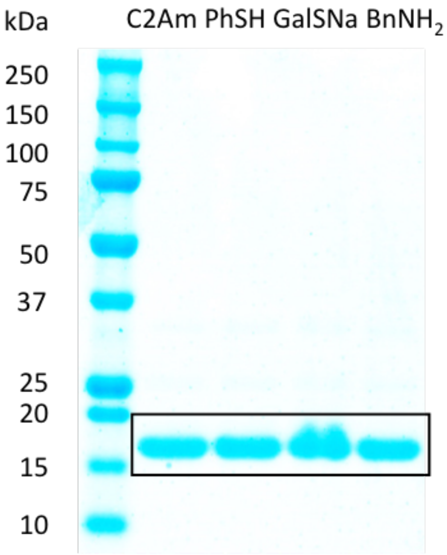

Figure S76. SDS-PAGE (MES SDS running buffer) analysis of starting and recovered C2Am after reaction of C2Am SCH_2-Ox (**10**) with PhSH, GalSNa, and $BnNH_2$

2.4. ^{1}H-NMR Experiments

Evaluation of lysine (Lys) cross-reactivity with 3-bromooxetane (1)

To a solution of Lys-peptide (**S1**) (1 mg, 2.4 µM) in deuterated NaP_i buffer (50 mM, pH 11, 600 µL) contained in an NMR tube, 3-bromooxetane (**1**) (1.5 equiv.) in 10% DMF-d_7 was added. After 36 h at 37 ºC, starting Lys-peptide (**S1**) and 3-bromooxetane (**1**) were detected unaltered.

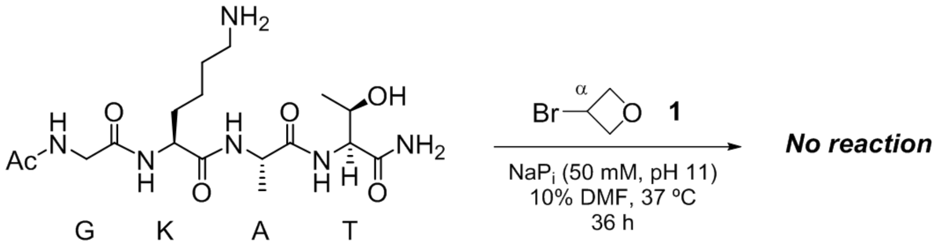

S1

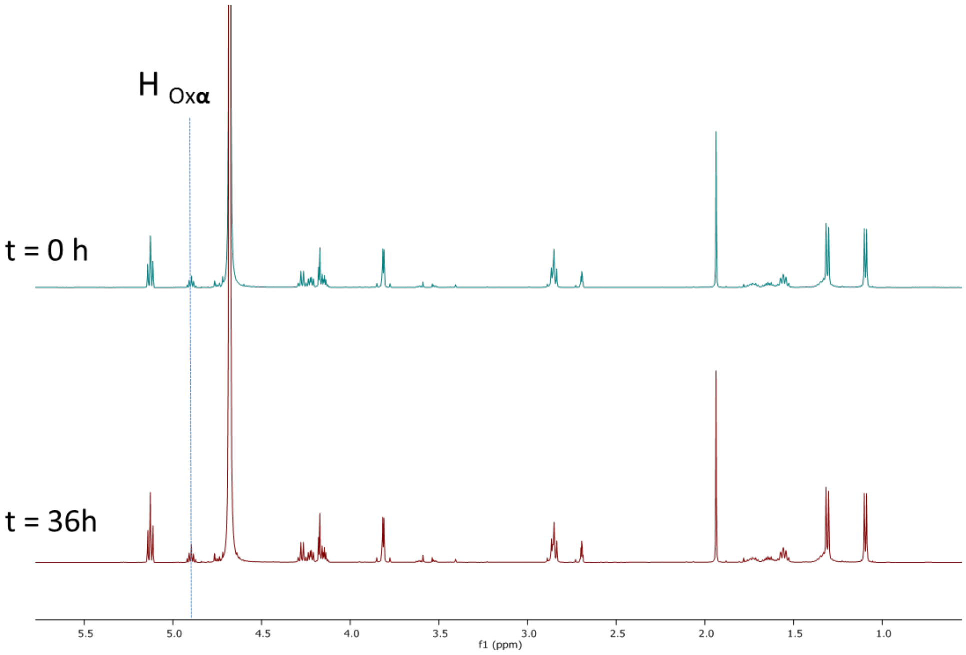

Figure S77. ^{1}H-NMR spectra superposition of the reaction of Lys-peptide (**S1**) with 3-bromooxetane (**1**).

Evaluation of Lys cross-reactivity with 3,3-bis(bromomethyl)oxetane (4)

To a solution of Lys-peptide (**S1**) (1 mg, 2.4 µM) in deuterated NaP_i buffer (50 mM, pH 11, 600 µL) contained in an NMR tube, 3,3-bis(bromomethyl)oxetane (**4**) (1.5 equiv.) in 10% DMF-d_7 was added. After 36 h at 37 ºC, starting Lys-peptide (**S1**) and 3,3-bis(bromomethyl)oxetane (**4**) were detected unaltered.

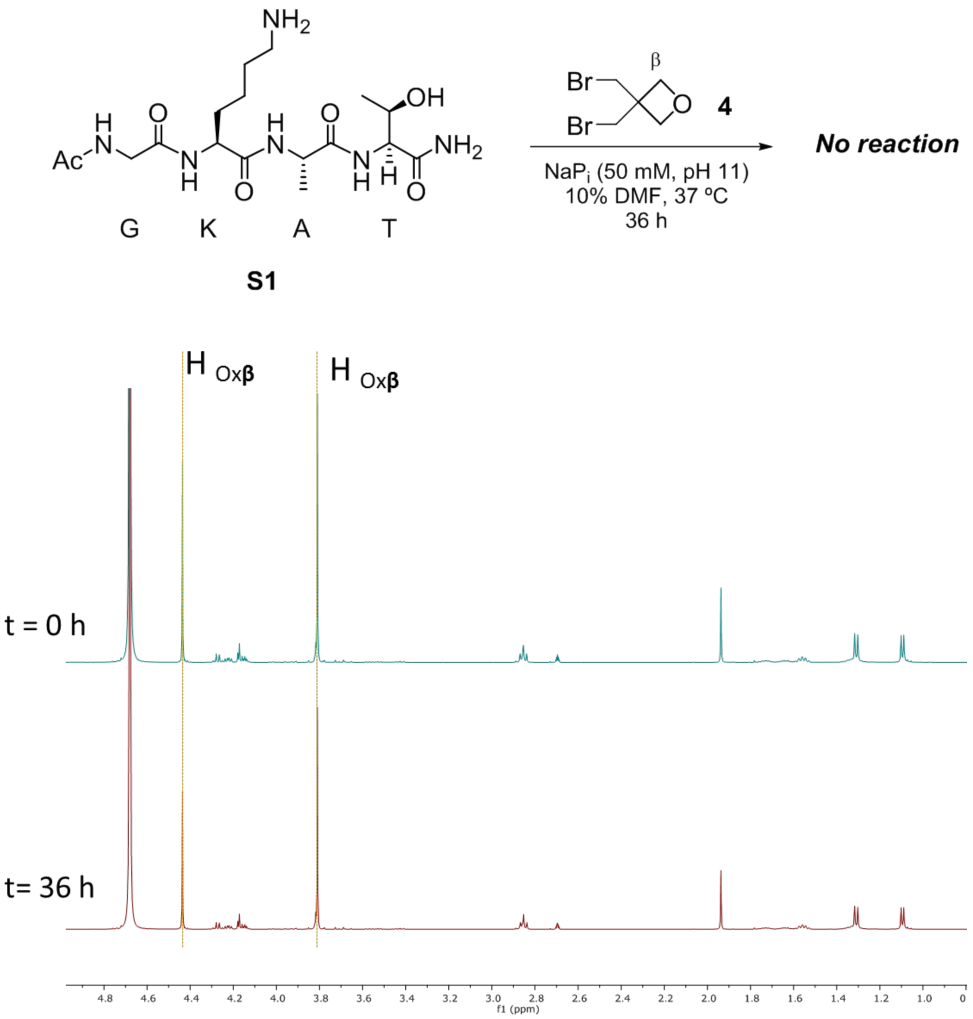

Figure S78. ^{1}H-NMR spectra superposition of the reaction of Lys-peptide (**S1**) with 3,3-bis(bromomethyl)oxetane (**4**).

Lysine (Lys) *vs.* cysteine (Cys) reactivity with 3-(bromomethyl)oxetane (9)

To a solution of Lys-peptide (**S1**) (1 mg, 2.4 µM) in deuterated NaP$_i$ buffer (50 mM, pH 8, 600 µL) contained in an NMR tube, 3-(bromomethyl)oxetane (**9**) (1.5 equiv.) in 10% DMF-d_7 was added. After 36 h at 37 ºC, starting Lys-peptide (**S1**) and 3-(bromomethyl)oxetane (**9**) were detected unaltered. Similarly, to a solution of Cys-peptide (**S4**) (1 mg, 2.5 µM) in deuterated NaP$_i$ buffer (50 mM, pH 8, 600 µL) contained in an NMR tube, 3-(bromomethyl)oxetane (**9**) (1.5 equiv.) in 10% DMF-d_7 was added. After 36 h at 37 ºC, changes in the chemical shift of the Hα of 3-(bromomethyl)oxetane (**9**) demonstrated the alkylation of Cys-peptide (**S4**) to peptide-SCH$_2$-Ox (**S5**) in >90% conversion.

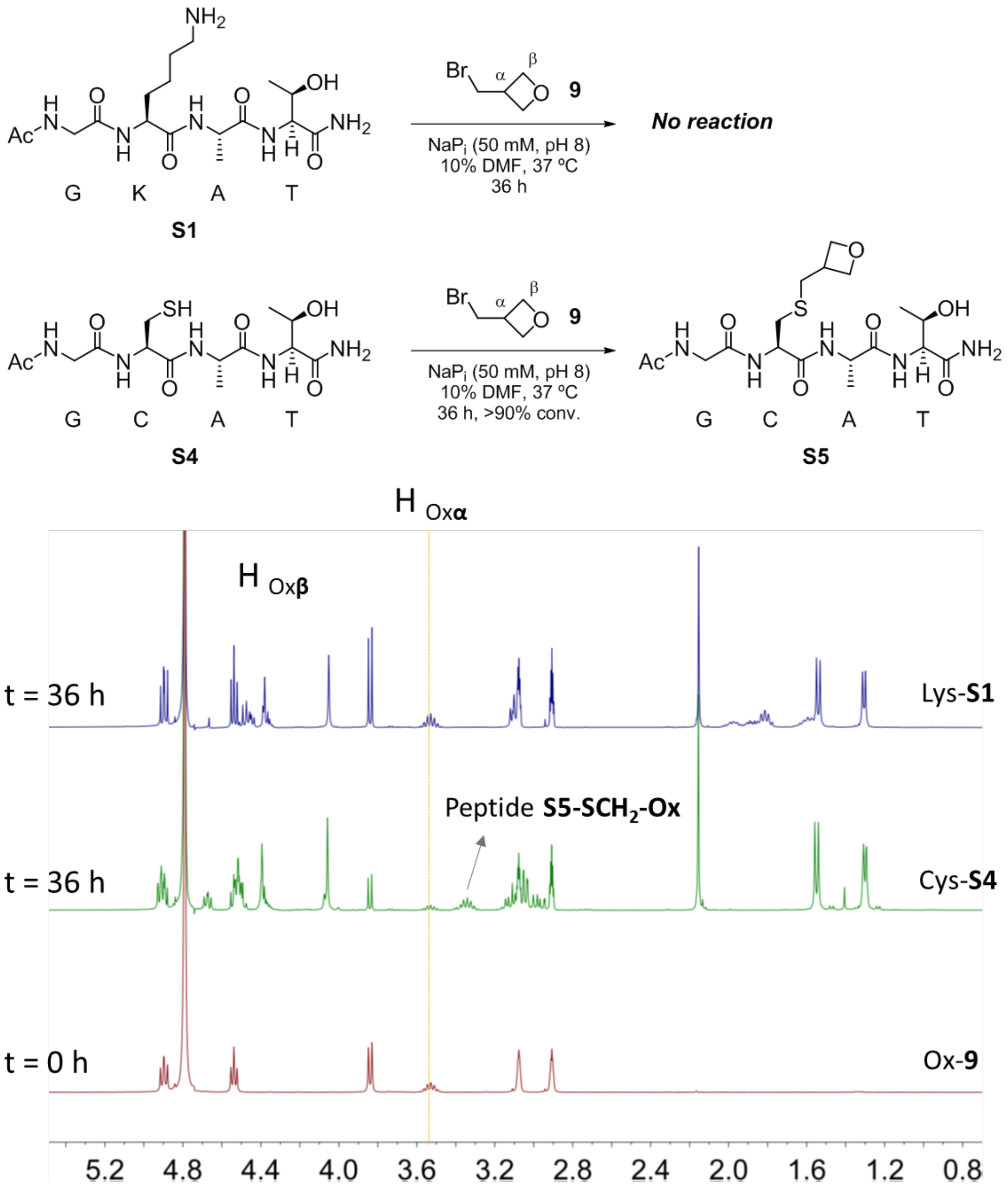

Figure S79. ^{1}H-NMR spectra superposition of the reaction of Lys-peptide (**S1**) and Cys-peptide (**S4**) with 3-(bromomethyl)oxetane (**9**).

2.5. Circular Dichroism (CD)

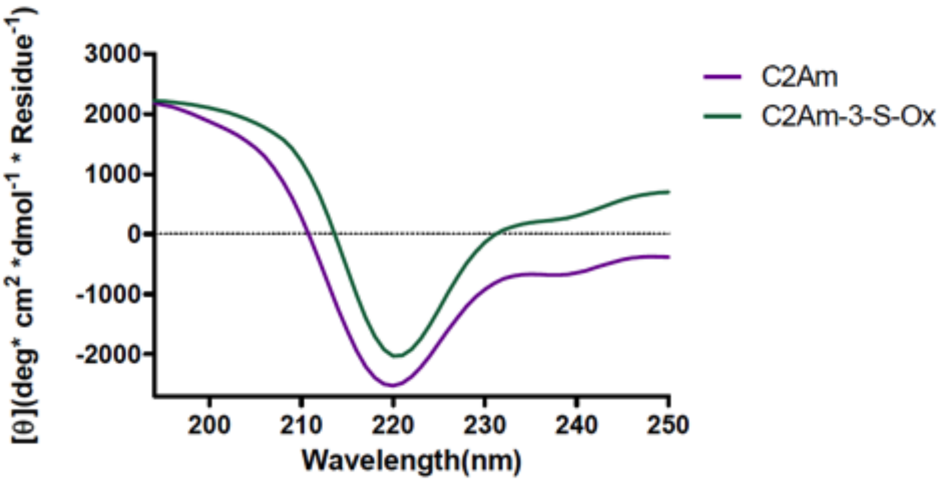

Figure S80. CD spectra of C2Am and C2Am 3-*S*-Ox (**2**) in sodium phosphate buffer (50 mM, pH 8). The final concentration of protein samples was 12 μM.

2.6. Molecular Dynamics (MD) Simulations

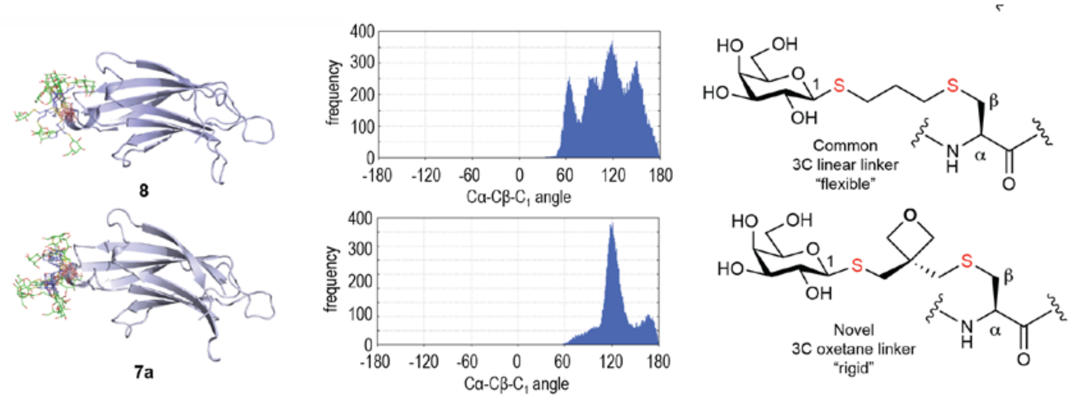

Figure S81. Analysis of the flexibility of *S*-to-*S*/*N* oxetane linker in C2Am-βGalSCH$_2$-*S*-Ox (**7a**) *vs.* a standard 3C-linear linker in C2Am-βGalS-(CH$_2$)$_3$ (**8**) obtained from 100 ns MD simulations. The data presented in this figure corresponds to the average structure of both linkers throughout the simulations.

2.7. Surface Plasmon Resonance (SPR)

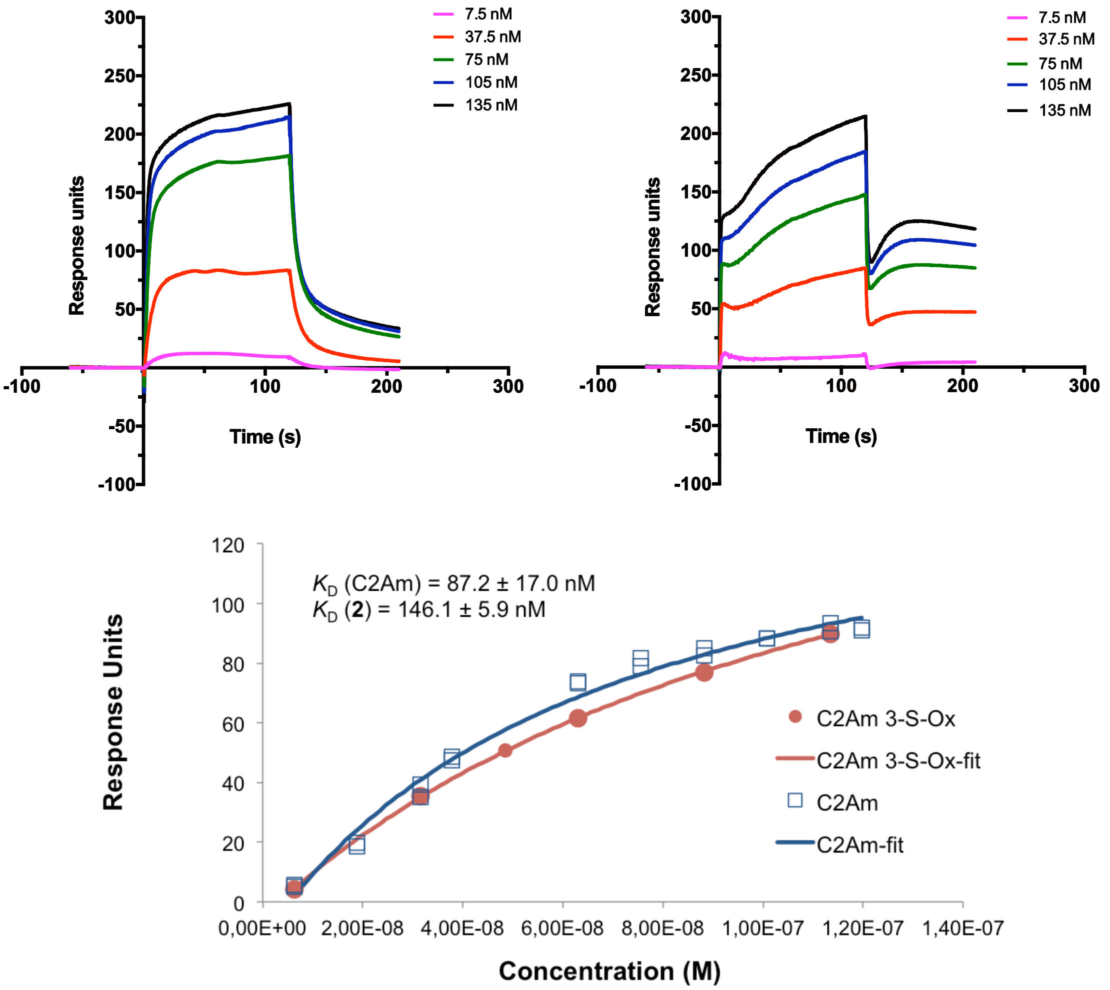

Figure S82. Biacore SPR curves obtained for C2Am (left) and C2Am 3-*S*-Ox (**2**) (right) against phosphatidylserine (PS), together with the K_D constants derived from SPR thermal equilibrium analysis.

2.8. Bio-Layer Interferometry (BLI)

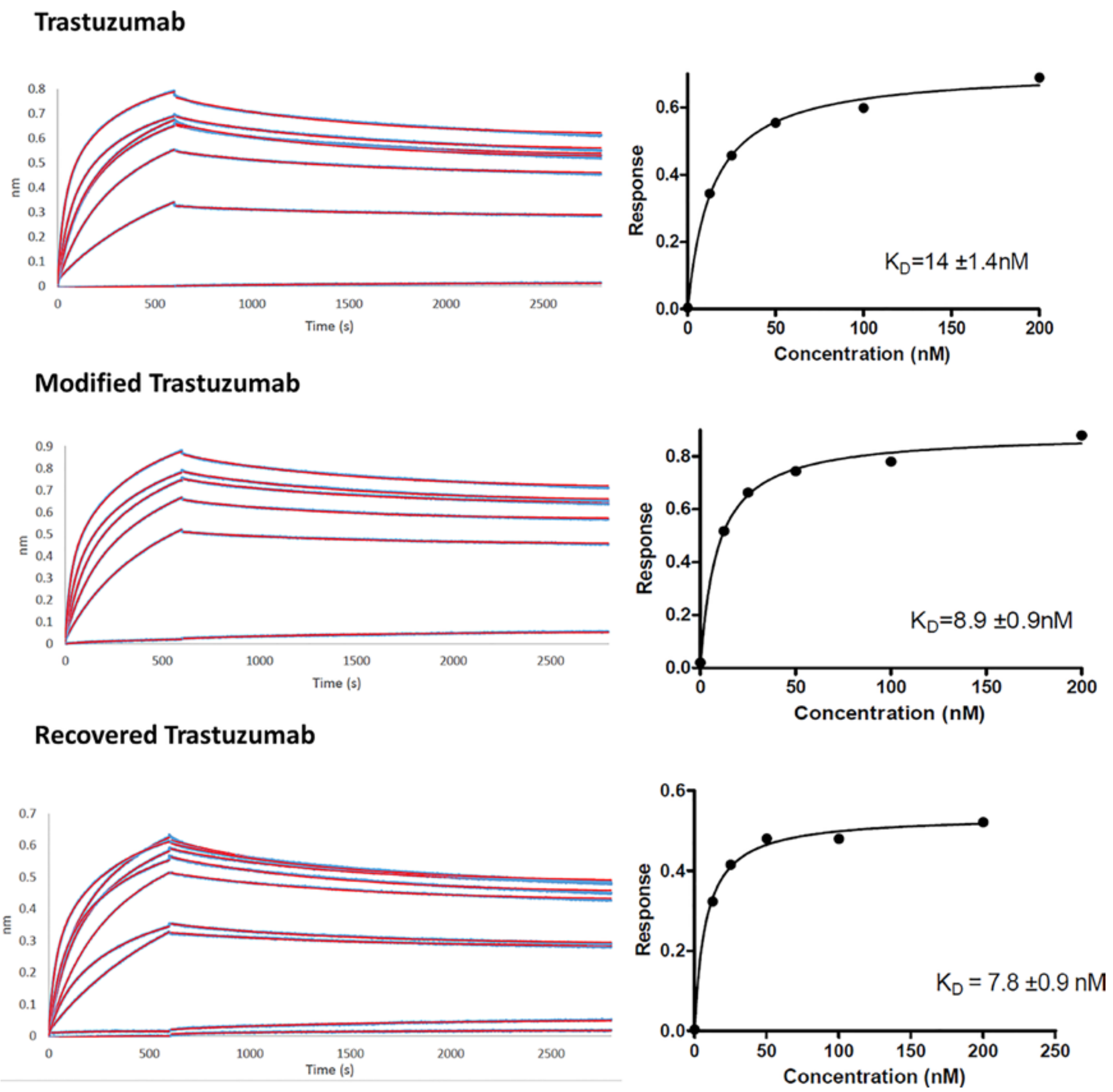

Figure S83. BLI curves (in blue) and fitting curves (in red) obtained for starting, modified **13**, and recovered Trastuzumab against HER2 receptor, together with the K_D constants derived from BLI experiments.

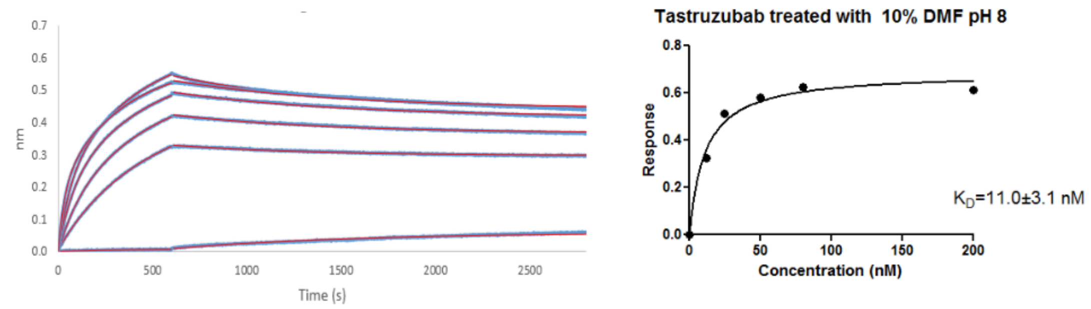

Figure S84. BLI curves (in blue) and fitting curves (in red) obtained for Trastuzumab treated under the same conditions (without the oxetane reagent) against HER2 receptor, together with the K_D constants derived from BLI experiments.

3. References

[1] Case, D. A.; Darden, T. A.; Cheatham, T. E.; Simmerling, C. L.; Wang, J.; Duke, R. E.; Luo, R.; Walker, R. C.; Zhang, W.; Merz, K. M.; Roberts, B.; Hayik, S.; Roitberg, A.; Seabra, G.; Swails, J.; Goetz, A. W.; Kolossváry, I.; Wong, K. F.; Paesani, F.; Vanicek, J.; Wolf, R. M.; Liu, J.; Wu, X.; Brozell, S. R.; Steinbrecher, T.; Gohlke, H.; Cai, Q.; Ye, X.; Wang, J.; Hsieh, M. J.; Cui, G.; Roe, D. R.; Mathews, D. H.; Seetin, M. G.; Salomon-Ferrer, R.; Sagui, C.; Babin, V.; Luchko, T.; Gusarov, S.; Kovalenko, A.; Kollman, P. A. AMBER 12 (2012).

[2] Hornak, V.; Abel, R.; Okur, A.; Strockbine, B.; Roitberg, A.; Simmerling, C. *Proteins* **2006**, *65*, 712–725.

[3] K. N. Kirschner, A. B. Yongye, S. M. Tschampel, J. González-Outeiriño, C. R. Daniels, B. L. Foley, R. J. Woods, *J. Comput. Chem.* **2008**, *29*, 622-655.

[4] J. Wang, R. M. Wolf, J. W. Caldwell, P. A. Kollman, D. A. J. Case *Comput. Chem.* **2004**, *25*, 1157−1174.

[5] Bayly, C. I.; Cieplak, P.; Cornell, W.; Kollman, P. A. *J. Phys. Chem.* **2002**, *97*, 10269–10280.

[6] Frisch, M. J.; Trucks, G. W.; Schlegel, H. B.; Scuseria, G. E.; Robb, M. A.; Cheeseman, J. R.; Scalmani, G.; Barone, V.; Mennucci, B.; Petersson, G. A.; Nakatsuji, H.; Caricato, M.; Li, X.; Hratchian, H. P.; Izmaylov, A. F.; Bloino, J.; Zheng, G.; Sonnenberg, J. L.; Hada, M.; Ehara, M.; Toyota, K.; Fukuda, R.; Hasegawa, J.; Ishida, M.; Nakajima, T.; Honda, Y.; Kitao, O.; Nakai, H.; Vreven, T.; Montgomery, J. A.; Peralta, J. E.; Ogliaro, F.; Bearpark, M.; Heyd, J. J.; Brothers, E.; Kudin, K. N.; Staroverov, V. N.; Kobayashi, R.; Normand, J.; Raghavachari, K.; Rendell, A.; Burant, J. C.; Iyengar, S. S.; Tomasi, J.; Cossi, M.; Rega, N.; Millam, J. M.; Klene, M.; Knox, J. E.; Cross, J. B.; Bakken, V.; Adamo, C.; Jaramillo, J.; Gomperts, R.; Stratmann, R. E.; Yazyev, O.; Austin, A. J.; Cammi, R.; Pomelli, C.; Ochterski, J. W.; Martin, R. L.; Morokuma, K.; Zakrzewski, V. G.; Voth, G. A.; Salvador, P.; Dannenberg, J. J.; Dapprich, S.; Daniels, A. D.; Farkas; Foresman, J. B.; Ortiz, J. V.; Cioslowski, J.; Fox, D. J. Gaussian 09, Revision B.01, Gaussian, Inc., Wallingford CT 2009.

[7] Jorgensen, W. L.; Chandrasekhar, J.; Madura, J. D.; Impey, R. W.; Klein, M. L. *J. Phys. Chem.* **1983**, *79*, 926–935.

[8] Andrea, T. A.; Swope, W. C.; Andersen, H. C. *J. Phys. Chem.* **1983**, *79*, 4576–4584.

[9] Darden, T.; York, D.; Pedersen, L. *J. Phys. Chem.* **1993**, *98*, 10089–10085.

[10] Alam, I. S.; Neves, A. A.; Witney, T. H.; Boren, J.; Brindle, K. M. *Bioconjugate Chem.* **2010**, *21*, 884–891.

[11] Oliveira, B. L.; Guo, Z.; Boutureira, O.; Guerreiro, A.; Jiménez-Osés, G.; Bernardes, G. J. L. *Angew. Chem. Int. Ed.* **2016**, *55*, 14683 –14687.

[12] Jin, M.; Smith, C.; Hsieh, H.-Y.; Gibson, D. F.; Tait, J. F. *J. Biol. Chem.* **2004**, *279*, 40351–40357.

[13] Coxon, K.; Duggan, J.; Cordeiro, M. F.; Moss, S. In *Cancer Cell Culture*; Cree, I. A., Ed.; Humana Press, 2011; Vol. 731; 293–308.

[14] Junutula, J. R.; Raab, H.; Clark, S.; Bhakta, S.; Leipold, D. D.; Weir, S.; Chen, Y.; Simpson, M.; Tsai, S. P.; Dennis, M. S.; Lu, Y.; Meng, Y. G.; Ng, C.; Yang, J.; Lee, C. C.; Duenas, E.; Gorrell, J.; Katta, V.; Kim, A.; McDorman, K.; Flagella, K.; Venook, R.; Ross, S.; Spencer, S. D.; Lee Wong, W.; Lowman, H. B.; Vandlen, R.; Sliwkowski, M. X.; Scheller, R. H.; Polakis, P.; Mallet, W. *Nat. Biotech.* **2008**, *26*, 925–932.
